# Expanding the scope of Metal-Free enantioselective allylic substitutions: Anthrones

**DOI:** 10.1038/srep16886

**Published:** 2015-11-23

**Authors:** Victor Ceban, Jiří Tauchman, Marta Meazza, Greg Gallagher, Mark E. Light, Ivana Gergelitsová, Jan Veselý, Ramon Rios

**Affiliations:** 1 School of Chemistry, University of Southampton, Highfield Campus SO17 1BJ, UK; 2 Department of Organic Chemistry, Charles University in Prague, 128 43 Praha 2, Czech Republic

## Abstract

The highly enantioselective asymmetric allylic alkylation of Morita–Baylis–Hillman carbonates with anthrones is presented. The reaction is simply catalyzed by cinchona alkaloid derivatives affording the final alkylated products in good yields and excellent enantioselectivities.

Asymmetric allylic substitution has been one of the most important tools in organic chemistry since its discovery by Tsuji and Trost in the early 70 s^1^,^2^. The versatility, chemical scope, functional group tolerance, predictability and often high stereoselectivities associated with this process make it an indispensable tool in organic synthesis. However, recently Green Chemistry has become one of the major focus in order to develop new reactions. Concepts like atom economy or sustainability have become new paradigms for a new generation of chemists. For this reason, one of the common approaches to Green Chemistry is to avoid the use of transition metals such as Pd, Rh, etc. Organocatalysis has blossomed since 2000 for this reason, hundreds of new reactions have been tailored under the auspices of Green Chemistry. One of the most intriguing approaches has been the development of a metal-free allylic substitution. The use of Morita-Baylis Hillman acetates or carbonates has been extensively studied as an alternative to the Tsuji-Trost reaction. In the present work, we expand the scope of the organocatalytic allylic substitution by investigating the use of anthrones.

Anthrones, important scaffolds in natural products and medicinal chemistry, are isolated either in free form or as *O*- or *C*-glycosides from a wide variety of natural sources (e.g., plants, shrubs) like rhubarb, cassia, cascara sagrada bark, etc^3^,^4^,^5^,^6^.

From a chemical standpoint, these compounds (or their enol forms) play a central role in anthracene chemistry due to the oxidation of the central ring that affords 9,10-anthraquinones, which are valuable scaffolds of pigments or anticancer agents^7^,^8^. Reduction of anthrones gives anthracenes, which are very useful compounds for the synthesis of dyes and optoelectronic materials^9^,^10^.

The dimerization of anthrones led to the synthesis of perylenequinone building blocks of hypericin, present in the herbal remedy St. John’s wort^11^,^12^,^13^,^14^. Finally, 9-oxoanthracene has been used in the determination of sugars. Moreover, many members of the anthrone family have interesting biological properties (e.g., antimicrobials, laxatives, antipsoriatics, telomerase inhibitors or selective antitumor activity)^15^,^16^,^17^,^18^,^19^.

Anthrone scaffolds can be synthesized by two basic strategies: Friedel-Crafts cyclization of *o*-benzylbenzoic acids or partial reduction of 9,10-anthraquinones. Several groups (Tamura, Cameron, Baldwin, Rodrigo, Snieckus) made fundamental contributions by using anthrones primarily in the synthesis of anthracyclinones, aglycones of the clinically important compound class anthracyclines^20^,^21^. Functionalization at C-10 commonly requires deprotonation with a base, which affords racemic anthrones^22^.

Despite the importance of anthrone moieties in medicinal chemistry, their asymmetric synthesis has not been extensively studied. Only few examples of asymmetric reactions have been reported. For example, anthrones usually behave as reactive dienes, which could react with a variety of dienophiles in the presence of a chiral base in aprotic solvents, reported for the first time by Rickborn^23^,^24^.

In previous years, several research groups have focused their attention on the development of asymmetric Diels-Alder reactions with anthrones by using several catalytic systems. Building upon Rickborn’s results, Riant and Kagan investigated the effect of chiral bases in the Diels-Alder reaction between anthrones and maleimides finding that quinine or prolinol were able to catalyze the reaction with moderate enantioselectivities^25^,^26^. Later on, Yamamoto^27^,^28^ reported the use of C2-chiral 2,5-bis(hydroxymethyl)pyrrolidines as catalysts and Tan and coworkers reported an extremely nice and efficient Diels-Alder reaction between anthrones and maleimides catalyzed by chiral bicyclic guanidines in both examples with excellent results^29^.

Less explored chemistry is the use of anthrones as nucleophiles in Michael addition. Shi and coworkers^30^ and our research group have reported the organocatalytic addition of anthrones to nitrostyrenes and to enals, respectively, with good results (Fig. 1)^31^,^32^,^33^.

Following our quest for the development of new methodologies for the synthesis of new three-dimensional scaffolds with possible applications in the pharmaceutical or agrochemical industries, and spurred on by our previous results, we envisioned that anthrones are an often missed privileged structure that could lead to very interesting structures.

Therefore, the development of efficient methods for the asymmetric synthesis of anthrones appears to be a worthy objective. As stated before, only a few examples of asymmetric reactions of anthrones or its derivatives have been reported, and most of them deal with base-catalyzed Diels-Alder reactions or Michael additions to electron-poor allenes.

In the light of these precedents and given our interest in the development of new methodologies for asymmetric organocatalysis based on our previous experience in MBH carbonates^34^,^35^,^36^, we decided to explore the reactivity of anthrones in organocatalyzed allylic substitutions. The reaction undergoes an S_N_2’-S_N_2’ Lewis-base-catalyzed pathway to render highly functionalized compounds, that are interesting scaffolds for medicinal and agrochemical industries.

## Results

Initial experiments were performed for the quinine-catalyzed reactions of anthrone with MBH carbonate **2a**. To our delight, the reaction rendered the desired compound in good conversions and with reasonable stereoselectivities after 1 week. Spurred by this result, we screened the best conditions for the reaction. We tested several Lewis bases, such as quinine, cinchonidine, β-ICD, or Sharpless ligands, at 50 °C or r.t. by using toluene as a solvent. The best catalyst was (DHQD)_2_AQN, which rendered the desired compound in 84% conversion and 82% *ee* (Table 1, entry 6). Further optimization of solvent and temperature was carried out. The best conditions were CH_2_Cl_2_ as the solvent at 0 °C, which furnished the final compound in full conversion and 88% *ee* after 5 d (Table 1, entry 15).

**Figure 2:**
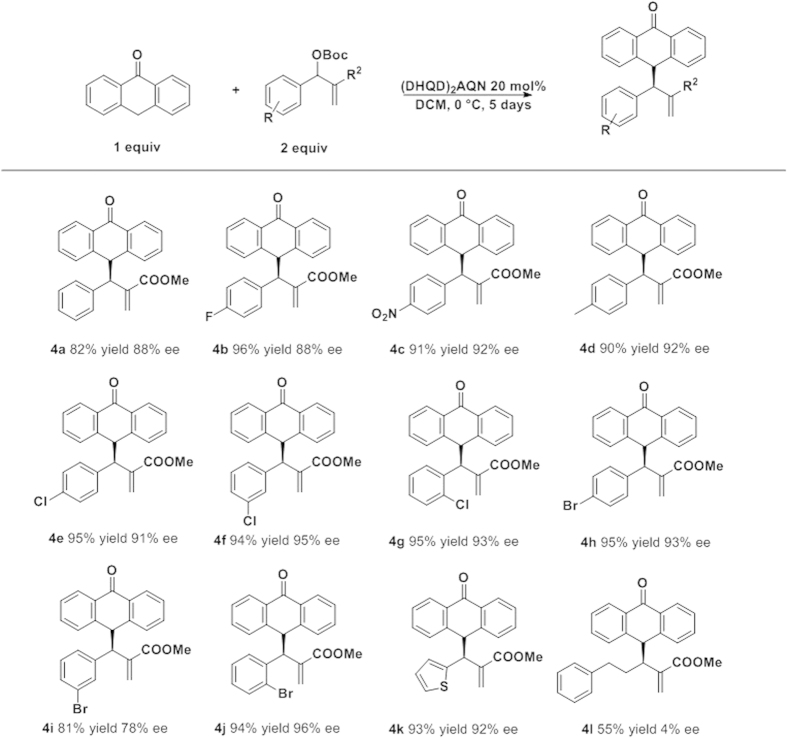
MBH aryl substituent scope.

With the best conditions in hand, we proceed to study the scope of the reaction in terms of the MBH carbonate. The reaction works fine with aromatic or heteroaromatic MBH carbonates in excellent yields and enantioselectivities (Fig. 2). The reaction tolerates several substituents on the aromatic ring, for example 4-methyl derivative afforded the final addition product **4d** in excellent yield and very good enantioselectivity (90% yield; 92% *ee*). Halide-substituted MBH carbonates rendered excellent results: 2-Cl, 3-Cl, and 4-Cl substrates gave the addition products **4e**–**g**, respectively, in excellent yields 94–95% and enantioselectivities (91–95%). In case of Br derivatives, the results are very similar (**4h**, **4j**), except for the 3-Br derivative (**4i**), which gave the worst enantioselectivity (81% yield, 78% *ee*). We also tested the reaction with a heteroaromatic derivative, which afforded the final compound **4k** with excellent yield and enantioselectivity. The only limitation is that the use of aliphatic MBH carbonates renders the final products in good yield but in an almost racemic form (**4l**).

**Figure 3:**
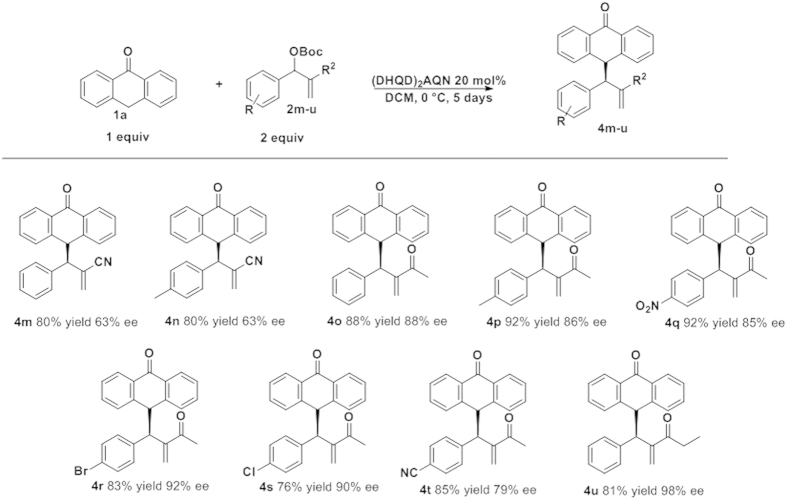
MBH electrowithdrawing substituent scope.

Next, we decided to study the scope of the reaction using MBH carbonates with different electron-withdrawing groups (Fig. 3). First, we tested the reaction with CN as an electron-withdrawing group. However, the results were disappointing: the final compounds were obtained with good yields and moderate enantioselectivities (**4m**, **4n**). Then we turned our attention to ketone derivatives. In general the results obtained were quite good. For example, compound **4o** was obtained in very good yield (88%) and *ee* (88%). The reaction with enones tolerates a wide range of substituents such as halides (**4r**, **4s**), electron-withdrawing (**4q**, **4t**) or electron-donating groups (**4p**) rendering, in all the examples, the final compounds in good yields (76–92% yield) and enantioselectivities 79–92% *ee*). Remarkably, when we used the ethylketone derivative (**4u**) we obtained the product with the highest *ee* (98% *ee*) and good yield (81%).

**Figure 4:**
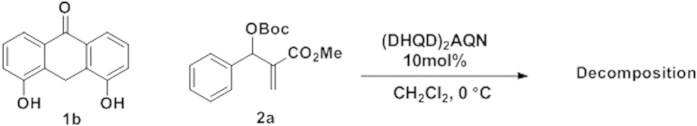
Dithranol reaction with MBH carbonates.

After we studied the scope of the reaction regarding the nature of the MBH carbonate, we proceed to study if we could expand the reaction to dithranol. Unfortunately when dithranol was used as a nucleophile the reaction gave complex mixtures (Fig. 4).

**Figure 5:**
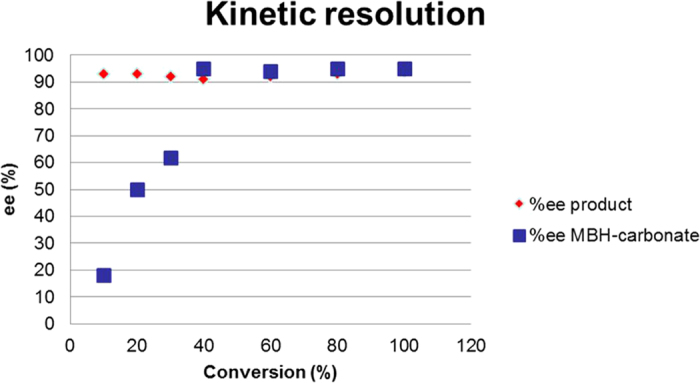
Kinetic studies of the anthrone addition to MBH carbonates.

To determine the mechanism of the reaction, we performed several experiments to study the behavior of the starting materials and products during the course of the reaction. We checked the enantioselectivity of the starting material and the final product at different stages to derive a plausible mechanism pathway. As shown in Fig. 5, the enantioselectivity of the final compound is independent from the extent of conversion. This data indicates a common diastereopure intermediate in the reaction. However, the starting material increased in enantiopurity with conversion. This behavior indicates a kinetic resolution of the MBH carbonate.

**Figure 6:**
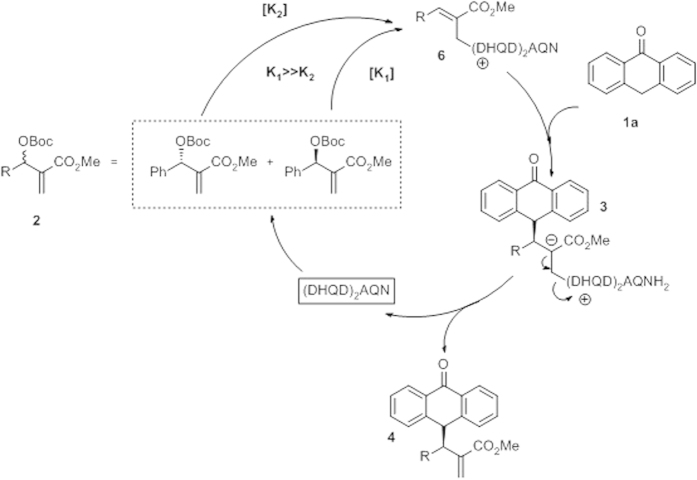
Proposed mechanism.

With this data in hand, we propose the following mechanism, which is in accordance with the accepted mechanism in similar reactions (Fig. 6).

First, substrate **2a** undergoes a conjugate addition, then elimination of the OBoc group to form CO_2_ and a *tert*-butoxide anion provides the Michael acceptor **6**. This step is responsible for the observed kinetic resolution of the MBH carbonates. Next, the nucleophile **1a** attacks the intermediate **6** to afford **3**, and the elimination of the organocatalyst gives the final product **4**.

**Figure 7:**
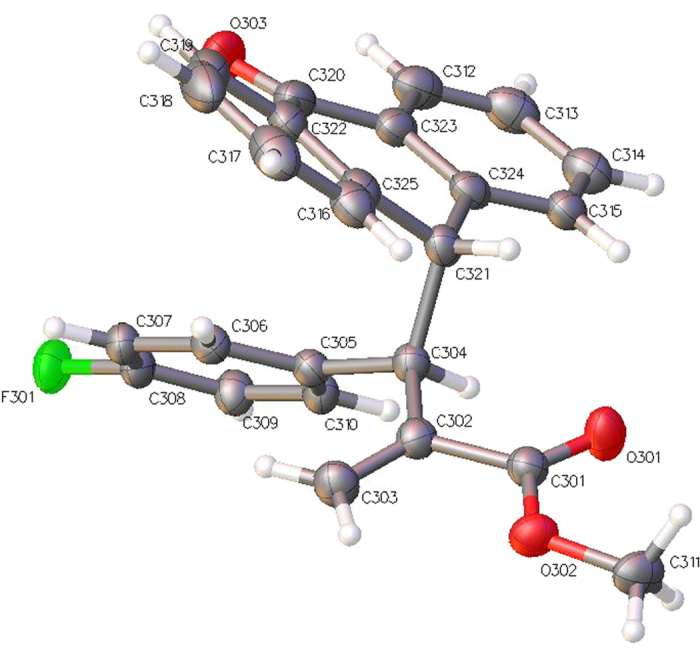
X-ray structure of compound 4b. The displacement ellipsoids are drawn at the 50% probability level.

The absolute configuration of compound **4b** was ascertained by single-crystal X-ray analysis (Fig. 7). The X-ray crystal structure unambiguously shows that the enantiomer obtained from the reaction with (DHQD)_2_AQN has an *R* configuration.

**Figure 8:**
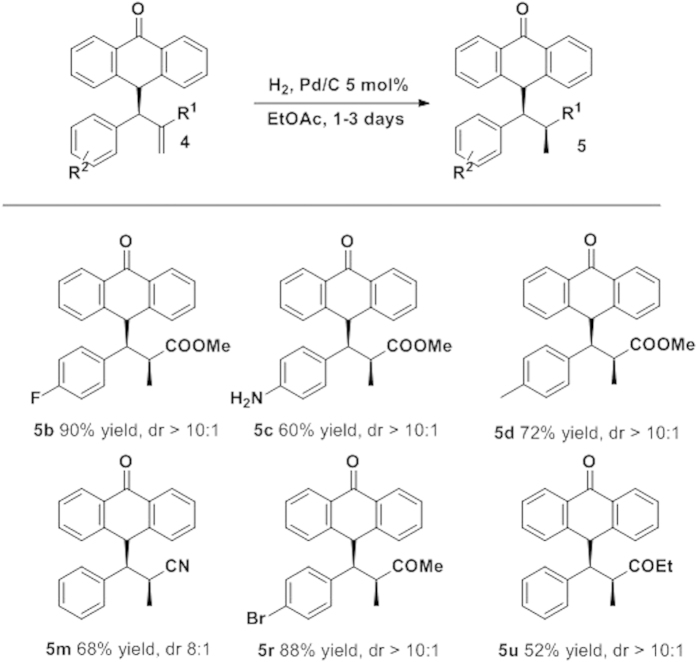
Hydrogenation of compounds 4 of the resulting adducts.

Next, we decided to study the applicability of the reaction by derivatization of compounds **4**. The reduction of the double bond was achieved by treatment of compounds **4** with Pd over H_2_, affording the hydrogenated compounds in excellent yields and excellent to good diastereoselectivities (Fig. 8). As it is shown in Fig. 8, in all the compounds the hydrogenation renders the final products in excellent diastereoselectivities. Interestingly, the carbonyl group of the anthrone remains unreduced. Only in the example **5c** a side reaction took place reducing the nitro group to amine. Remarkably the reaction shows a good group tolerance including halogens (**5b**,**5r**), cyano derivatives (**5m**) and ketones (**5r** and **5u**) giving the final reduced products as almost diastereopure and with moderate to good yields (52–90%).

**Figure 9:**
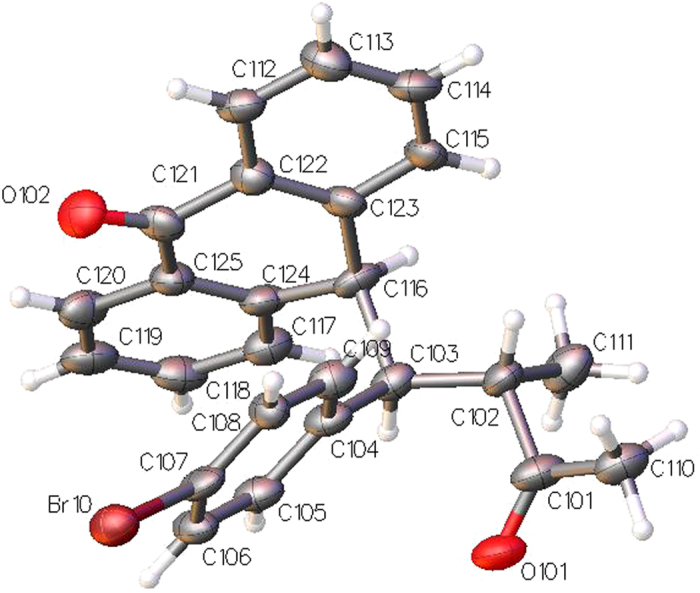
X-ray structure of compound 5r. The displacement ellipsoids are drawn at the 50% probability level.

The relative configuration of compound **5r** was ascertained by single-crystal X-ray analysis (Fig. 9). The X-ray crystal structure unambiguously shows that the diastereomer obtained from the hydrogenation of **4r** has an (*S*, *S*) absolute configuration.

## Methods

General Procedure for the racemic addition of anthrones to MBH carbonates: to a solution of anthrone (1equiv, 0.1 mmol) in dichloromethane (0.1 mol/L) was added the appropriate MBH-carbonate (2 equiv, 0.2 mmol) and 1,4-diaza-bicyclo[2.2.2]octane (20 mol%, 0.02 mmol).The reaction was stirred for 3 days at 0 °C. The reaction was followed by NMR until the disappearance of starting material. The reaction mixture was purified by column chromatography (mixtures Hexane/EtOAc).

General procedure for the chiral addition of anthrones to MBH carbonates: to a solution of anthrone (1equiv, 0.1 mmol) in dichloromethane (0.1 mol/L) was added the appropriate MBH-carbonate (2 equiv, 0.2 mmol) and (DHQD)_2_AQN (20 mol%, 0.02 mmol).The reaction was stirred for 5 days at 0 °C. The reaction was followed by NMR until the disappearance of starting material. The reaction mixture was purified by column chromatography (mixtures Hexane/EtOAc).

General procedure for the hydrogenation of products **4**: In a 2-neck round-bottom flask (RBF) product **4** (1.0 equiv) was added together with Pd/C (0.05 equiv w/w). EtOAc (1 mL, 0.065 mol/L) was added. The RBF was sealed and flushed twice with Argon. Then the reaction mixture was flushed once with H_2_. The rubber balloon was refilled with H_2_ and connected to the RBF. The reaction was monitored by TLC until the reaction completion. The mixture was filtered through Celite, washed with EtOAc and concentrated in vacuo. The mixture was purified by column chromatography (5:1 Hexane:EtOAc) (see Supplementary Information).

Crystal Data for C_25_H_19_FO_3_ (M = 386.40 g/mol) (**4b**): orthorhombic, space group P212121 (no. 19), a = 11.07781(8) Å, b = 12.29676(10) Å, c = 42.1557(3) Å, V = 5742.50(8) Å3, Z = 12, T = 100(2) K, μ(CuKα) = 0.767 mm-1, Dcalc = 1.341 g/cm3, 77409 reflections measured (4.192° ≤ 2Θ ≤ 137.932°), 10586 unique (Rint = 0.0403, Rsigma = 0.0152) which were used in all calculations. The final R1 was 0.0319 (I > 2σ(I)) and wR2 was 0.0832 (all data). Crystallographic data (excluding structure factors) for the structure **4b** have been deposited with the Cambridge Crystallographic Data Centre with CCDC number 1062129. Copies of the data can be obtained, free of charge, on application to Cambridge Crystallographic Data Centre, 12 Union Road, Cambridge CB2 1EZ, UK, (fax: +44-(0)1223-336033 or e-mail: deposit@ccdc.cam.ac.uk).

Crystal Data for C_25_H_21_BrO_2_ (M = 433.33 g/mol) (**5r**): orthorhombic, space group C2221 (no. 20), a = 14.1137(4) Å, b = 14.1141(5) Å, c = 39.2592(13) Å, V = 7820.5(4) Å3, Z = 16, T = 100(2) K, μ(CuKα) = 3.001 mm-1, Dcalc = 1.472 g/cm3, 57094 reflections measured (4.502° ≤ 2Θ ≤ 137.968°), 7193 unique (Rint = 0.1076, Rsigma = 0.0386) which were used in all calculations. The final R1 was 0.0663 (I > 2σ(I)) and wR2 was 0.1868 (all data). Crystallographic data (excluding structure factors) for the structure **5r** have been deposited with the Cambridge Crystallographic Data Centre with CCDC number 1403893–1403894. Copies of the data can be obtained, free of charge, on application to Cambridge Crystallographic Data Centre, 12 Union Road, Cambridge CB2 1EZ, UK, (fax: +44-(0)1223-336033 or e-mail: deposit@ccdc.cam.ac.uk).

## Additional Information

**How to cite this article**: Ceban, V. *et al.* Expanding the scope of Metal-Free enantioselective allylic substitutions: Anthrones. *Sci. Rep.* **5**, 16886; doi: 10.1038/srep16886 (2015).

## Supplementary Material

Supplementary Information

## Figures and Tables

**Figure 1:**
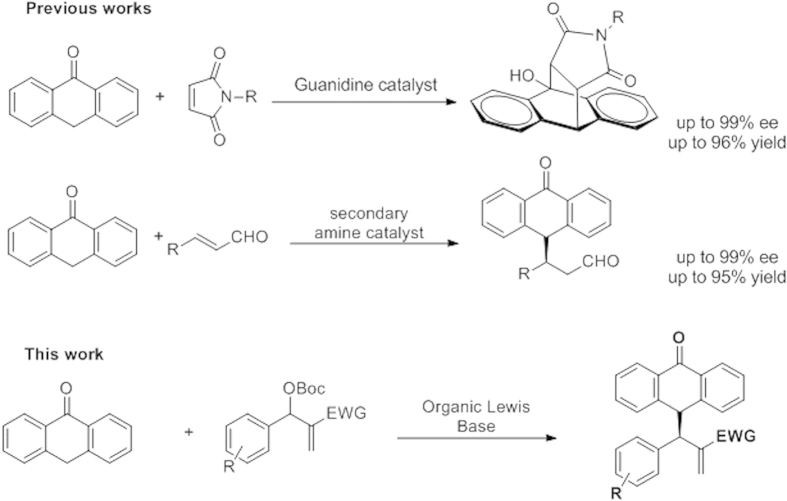
Reactions with Anthrones.

**Table 1:**
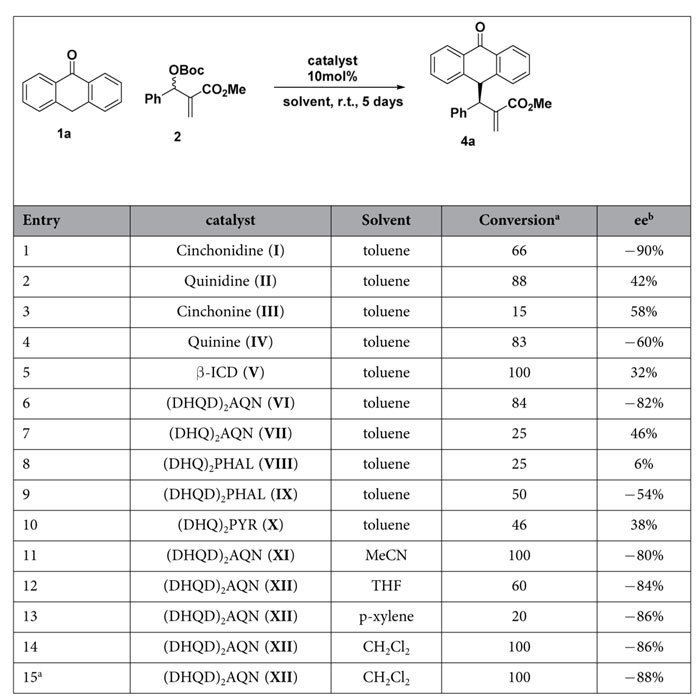
Screening.

^a^Conversion determined by ^1^H NMR analysis of the crude. ^b^Enantioselectivity determined by chiral HPLC analysis of the crude mixture. ^c^Reaction performed at 0 °C.
