# Supplementary material for: Expanding the scope of Metal-Free enantioselective allylic substitutions: Anthrones
Source: Sci Rep. 2015 Nov 23;5:16886. doi: 10.1038/srep16886 (PMC4655356; doi:10.1038/srep16886)

Expanding the scope of Metal-Free enantioselective allylic substitutions: Anthrones

Victor Ceban[a], Jirí Tauchman[a,b], Greg Gallagher[a], Marta Meazza, Mark E. Light[a], Jan Vessely[b], Ramon Rios*

[a] *School of Chemistry, University of Southampton, Southampton, SO17 1BJ, UK.*

[b] *Department of Organic Chemistry, Faculty of Science, Charles University in Prague, Hlavova 2030, 128 43 Praha 2, Czech Republic.*

Supporting information

Table of Contents

General 2

General procedure for the synthesis of chiral products 3

Hydrogenation reaction 18

Kinetic resolution 22

NMR spectra 23

NMR Spectra for Hydrogenation 45

HPLC Chiral data 52

X-Ray data 73

General

The general reaction is:

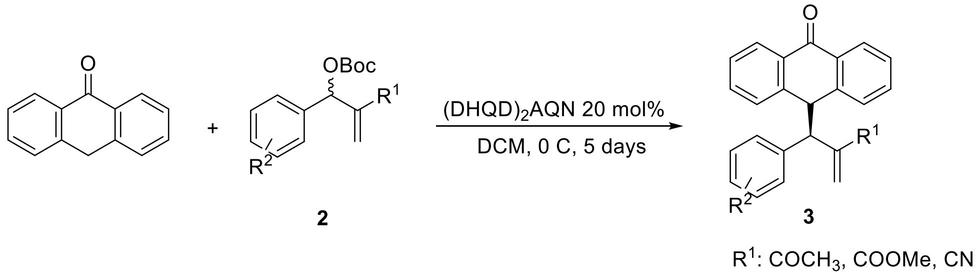

The MBH-carbnoates were synthesized and analysed following the procedure from the articles:

"Construction of adjacent quaternary and tertiary stereocenters via an organocatalytic allylic alkylation of Morita-Baylis-Hillman carbonates" (*Adv. Synth. Catal.* **2007**, 349, 281 – 286),[19]

"Traditional Morita–Baylis–Hillman reaction of aldehydes with methyl vinyl ketone co-catalyzed by triphenylphosphine and nitrophenol" (*Org. Biomol. Chem.* **2006**, 4, 1468–1470).[20]

Thin layer chromatography (TLC) was performed on Merck TLC Silicagel 60 F_{254}. Product spots were visualized by UV-light at 254nm, and developed with potassium permanganate. Column chromatography was effectuated using silica gel (Geduran Si60, 40-63µm).

Infra-red spectra were recorded on a Nicolet 280 FT-IR and Nicolet AVATAR 370 FT IR using KBr pellets.

1H-NMR, 13C-NMR, 19F-NMR were recorded with Bruker AV300, Bruker DPX400 and Bruker AVANCE III 600. Chemical shifts (σ/ppm) are given relative to the residual peak of the NMR solvent ($CDCl_3$: σ_H = 7.26 ppm, σ_C = 77.16 ppm).

High resolution mass spectra were recorded using Esquire 3000 (Bruker; low resolution) or an LTQ Orbitrap XL instrument (Thermo Fisher Scientific; high resolution) and MaXis (Bruker Daltonics, Bremen, Germany) mass spectrometer equipped with a Time of Flight (TOF) analyser.

Chiral HPLC was performed with an LCP 5020 Ignos liquid chromatography pump with an LCD 5000 spectrophotometric detector with Daicel Chiralpak® columns also with Perkin Elmer HPLC with Daicel Chiralpak® columns.

General procedure for the synthesis of racemic products

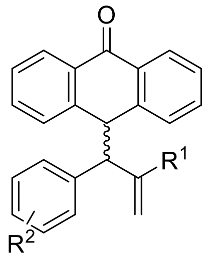

To a solution of anthrone (1equiv, 19 mg, 0.1 mmol) in dichloromethane (0.1 mol/L) was added the appropriate MBH-carbonate (2 equiv, 0.2 mmol), 1,4-diaza-bicyclo[2.2.2]octane (20 mol%, 2 mg, 0.02 mmol).The reaction was stirred for 3 days at room temperature. The reaction was followed by NMR until the disappearance of starting material. The reaction mixture was purified by column chromatography (20:1 Hexane/EtOAc).

General procedure for the synthesis of chiral products

methyl (R)-2-((10-oxo-9,10-dihydroanthracen-9-yl)(phenyl)methyl)acrylate (4a)

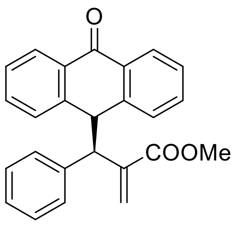

To a solution of anthrone (1equiv, 19 mg, 0.1 mmol) in dichloromethane (0.1 mol/L) was added methyl 2-(((tert-butoxycarbonyl)oxy)(phenyl)methyl)acrylate (2 equiv, 58 mg, 0.2 mmol), $(DHQD)_2AQN$ (20 mol%, 14 mg, 0.02 mmol). The reaction was stirred for 5 days at 0 °C. The reaction was followed by NMR until the disappearance of starting material. The reaction mixture was purified by column chromatography (20:1 Hexane/EtOAc) to obtain 30 mg of desired product. The product yield is 82 %. ^{1}H NMR (400 MHz, $CDCl_3$): σ 8.15 (dd, J = 7.5, 1.6 Hz, 1H), 7.97 (dd, J = 7.8, 1.0 Hz, 1H), 7.63 (d, J = 7.6 Hz, 1H), 7.55 (td, J = 7.5, 1.3 Hz, 1H), 7.46 (ddd, J = 9.0, 7.3, 1.6 Hz, 2H), 7.38 – 7.31 (m, 1H), 7.25 – 7.19 (m, 1H), 7.09 (t, J = 7.4 Hz, 1H), 6.96 (t, J = 7.7 Hz, 2H), 6.32 (s, 1H), 6.30 (d, J = 7.3 Hz, 2H), 5.37 (d, J = 1.1 Hz, 1H), 4.96 (d, J = 4.6 Hz, 1H), 4.48 (d, J = 4.5 Hz, 1H), 3.89 (s, 3H). ^{13}C NMR (101 MHz, $CDCl_3$) σ 184.3 (q), 168.0 (q), 144.0 (q), 141.3 (q), 140.2 (q), 135.8 (q), 134.1 (q), 132.9 (q), 132.4, 131.5, 130.0, 129.1, 128.4 (CH_2), 128.3, 127.8, 127.4, 127.3, 127.1, 126.9, 126.6, 58.4, 52.4, 46.8 (n-Hexane traces 31.6, 22.7, 14.1). HR-MS (m/z) for $C_{25}H_{20}O_3$ calculated $[M+H]^+$ 369.1485, measured $[M+H]^+$ 369.1489. IR (cm^{-1}): 3064 (=C-H, stretch), 3029 (=C-H, stretch), 1715 (C=O, stretch), 1666 (C=O, stretch), 1600 (C=C, stretch), 1313 (C-O ester, stretch). $[\alpha]_D^{19}$ = +48.4 (c = 0.015 g/ml, $CHCl_3$). The enantiomeric excess was determined by HPLC using a Chiralpak IC column [hexane/*i*PrOH = 90:10]; flow rate 1.0 mL/min; 254 nm; t_{r1}= 14.2, t_{r2} = 16.9; 88 % ee.

methyl (R)-2-((4-fluorophenyl)(10-oxo-9,10-dihydroanthracen-9-yl)methyl)acrylate (4b)

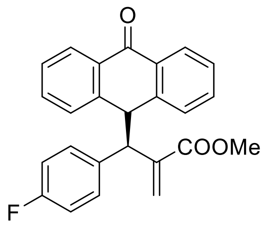

To a solution of anthrone (1equiv, 19 mg, 0.1 mmol) in dichloromethane (0.1 mol/L) was added methyl 2-(((tert-butoxycarbonyl)oxy)(4-fluorophenyl)methyl)acrylate (2 equiv, 60 mg, 0.2 mmol), $(DHQD)_2AQN$ (20 mol%, 14 mg, 0.02 mmol). The reaction was stirred for 5 days at 0 °C. The reaction was followed by NMR until the disappearance of starting material. The reaction mixture was purified by column chromatography (20:1 Hexane/EtOAc) to obtain 37 mg of desired product. The product yield is 96 %. ^{1}H NMR (400 MHz, $CDCl_3$) σ 8.16 (dd, *J* = 7.5, 1.6 Hz, 1H), 7.99 (dd, *J* = 7.8, 1.0 Hz, 1H), 7.65 (d, *J* = 7.6 Hz, 1H), 7.57 (m, 1H), 7.53 – 7.42 (m, 2H), 7.39 – 7.32 (m, 1H), 7.21 (d, *J* = 7.5 Hz, 1H), 6.65 (dd, *J* = 8.7 Hz, 2H), 6.32 (s, 1H), 6.28 – 6.19 (m, 2H), 5.31 (d, *J* = 1.3 Hz, 1H), 4.94 (d, *J* = 4.6 Hz, 1H), 4.46 (d, *J* = 4.5 Hz, 1H), 3.91 (s, 3H). ^{13}C NMR (101 MHz, $CDCl_3$) σ 184.1 (q), 167.9 (q), 143.9 (q), 140.9 (q), 140.3 (q), 134.0 (q), 132.8 (q), 132.7 (q), 132.6, 131.6, 131.48, 131.4 (q), 131.4, 129.1, 128.3, 128.2 (CH_2) 127.6, 127.1, 127.0, 126.8, 114.8, 114.6, 57.8, 52.5, 46.6. ^{19}F NMR (376 MHz, $CDCl_3$) σ -114.7. HR-MS (m/z) for $C_{25}H_{19}FO_3$ calculated $[M+H]^+$ 387.1391, measured $[M+H]^+$ 387.1387. IR (cm^{-1}): 3069 (=C-H, stretch), 3029 (=C-H, stretch), 1715 (C=O, stretch), 1666 (C=O, stretch), 1600 (C=C, stretch), 1314 (C-O ester, stretch). $[\alpha]_D^{19}$ = +16.7 (c = 0.019 g/ml, $CHCl_3$). The enantiomeric excess was determined by HPLC using a Chiralpak IC column [hexane/*i*PrOH = 90:10]; flow rate 1.0 mL/min; 254 nm; t_{r1}= 12.2, t_{r2} = 13.4; 88 % ee.

methyl (R)-2-((4-nitrophenyl)(10-oxo-9,10-dihydroanthracen-9-yl)methyl)acrylate (4c)

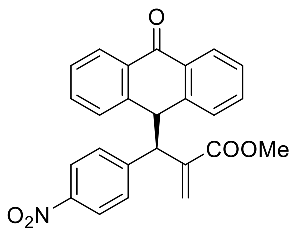

To a solution of anthrone (1equiv, 19 mg, 0.1 mmol) in dichloromethane (0.1 mol/L) was added methyl 2-(((tert-butoxycarbonyl)oxy)(4-nitrophenyl)methyl)acrylate (2 equiv, 60 mg, 0.2 mmol), $(DHQD)_2AQN$ (20 mol%, 14 mg, 0.02 mmol). The reaction was stirred for 5 days at 0 °C. The reaction was followed by NMR until the disappearance of starting material. The reaction mixture was purified by column chromatography (20:1 Hexane/EtOAc) to obtain 37 mg of desired product. The product yield is 91 %. ^{1}H NMR (400 MHz,

CDCl$_3$) σ 8.20 – 8.13 (m, 1H), 7.99 (dd, *J* = 7.8, 0.9 Hz, 1H), 7.83 (d, *J* = 8.8 Hz, 2H), 7.64 (d, *J* = 7.5 Hz, 1H), 7.58 (m, 1H), 7.54 – 7.44 (m, 2H), 7.44 – 7.34 (m, 1H), 7.20 (dd, *J* = 6.0, 2.8 Hz, 1H), 6.52 (d, *J* = 8.8 Hz, 2H), 6.36 (s, 1H), 5.30 (d, *J* = 1.3 Hz, 1H), 5.02 (d, *J* = 5.0 Hz, 1H), 4.56 (d, *J* = 4.9 Hz, 1H), 3.91 (s, 3H). ^{13}C NMR (101 MHz, CDCl$_3$) σ 184.0, 167.4, 147.0, 143.8, 143.2, 140.4, 139.3, 133.8 (q), 132.8, 132.6 (q), 131.7, 130.7, 129.1, 128.6 (CH$_2$), 128.2, 127.9, 127.5, 127.4, 127.1, 122.9, 58.6, 52.6, 46.3. HR-MS (m/z) for $C_{25}H_{19}NO_5$ calculated [M+H]$^+$ 414.1336, measured [M+H]$^+$ 414.1339. IR (cm^{-1}): 3075 (=C-H, stretch), 3029 (=C-H, stretch), 1713 (C=O, stretch), 1665 (C=O, stretch), 1600 (C=C, stretch), 1520 (N-O, asymmetric stretch), 1346 (N-O, symmetric stretch), 1313 (C-O ester, stretch). $[\alpha]_D^{19}$ = +34.5 (c = 0.019 g/ml, CHCl$_3$). The enantiomeric excess was determined by HPLC using a Chiralpak IB column [hexane/*i*PrOH = 95:5]; flow rate 1.0 mL/min; 254 nm; t_{r1}= 14.9, t_{r2} = 16.0; 92 % ee.

<u>methyl (R)-2-((10-oxo-9,10-dihydroanthracen-9-yl)(p-tolyl)methyl)acrylate</u> (4d)

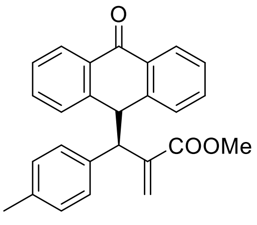

To a solution of anthrone (1equiv, 19 mg, 0.1 mmol) in dichloromethane (0.1 mol/L) was added methyl 2-(((tert-butoxycarbonyl)oxy)(p-tolyl)methyl)acrylate (2 equiv, 60 mg, 0.2 mmol), (DHQD)$_2$AQN (20 mol%, 14 mg, 0.02 mmol). The reaction was stirred for 5 days at 0 °C. The reaction was followed by NMR until the disappearance of starting material. The reaction mixture was purified by column chromatography (20:1 Hexane/EtOAc) to obtain 30 mg of desired product. The product yield is 90 %. ^{1}H NMR (400 MHz, CDCl$_3$) σ 8.15 (dd, *J* = 7.5, 1.6 Hz, 2H), 7.99 (dd, *J* = 7.8, 1.1 Hz, 2H), 7.63 (d, *J* = 7.6 Hz, 2H), 7.55 (td, *J* = 7.6, 1.4 Hz, 2H), 7.52 – 7.40 (m, 5H), 7.37 – 7.31 (m, 2H), 7.25 – 7.20 (m, 2H), 6.77 (d, *J* = 7.9 Hz, 4H), 6.30 (s, 2H), 6.19 (d, *J* = 8.1 Hz, 4H), 5.34 (d, *J* = 0.8 Hz, 2H), 4.93 (d, *J* = 4.6 Hz, 2H), 4.46 (d, *J* = 4.4 Hz, 2H), 3.88 (s, 6H), 2.19 (s, 6H). ^{13}C NMR (101 MHz, CDCl$_3$) σ 184.4 (q), 168.0 (q), 144.1 (q), 141.4 (q), 140.5 (q), 136.8 (q), 134.0 (q), 132.9 (q), 132.7 (q), 132.4, 131.5, 129.8, 129.2, 128.5, 128.4, 128.3 (CH$_2$), 127.4, 127.0, 126.9, 126.6, 58.0, 52.3, 46.8, 21.0. HR-MS (m/z) for $C_{26}H_{22}O_3$ calculated [M+H]$^+$ 383.1642, measured [M+H]$^+$ 383.1650. IR (cm^{-1}): 3056 (=C-H, stretch), 3026 (=C-H, stretch), 1715 (C=O, stretch), 1666 (C=O, stretch), 1600 (C=C, stretch), 1314 (C-O ester, stretch). $[\alpha]_D^{19}$ = +60.4 (c = 0.015 g/ml, CHCl$_3$). The enantiomeric excess

was determined by HPLC using a Chiralpak IC column [hexane/*i*PrOH = 97:3]; flow rate 1.0 mL/min; 254 nm; t_{r1}= 27.3, t_{r2} = 30.9; 92 % ee.

methyl (R)-2-((4-chlorophenyl)(10-oxo-9,10-dihydroanthracen-9-yl)methyl)acrylate (4e)

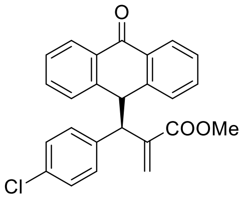

To a solution of anthrone (1 equiv, 49 mg, 0.25 mmol) in dichloromethane (0.25 mol/L) was added methyl 2-(((tert-butoxycarbonyl)oxy)(4-chlorophenyl)methyl)acrylate (2 equiv, 165 mg, 0.50 mmol), $(DHQD)_2AQN$ (20 mol%, 43 mg, 0.05 mmol). The reaction was stirred for 7 days at 0 °C. The reaction was followed by NMR until the disappearance of starting material. The reaction mixture was concentrated in vacuo and purified by column chromatography (20:1 Hexane/EtOAc) to obtain 96 mg of desired product. The product yield is 95 %. ^{1}H NMR (600 MHz, $CDCl_3$): σ 8.16 (d, *J* = 7.5 Hz, 1 H), 8.01 (d, *J* = 7.6 Hz, 1 H), 7.62 (d, *J* = 7.7 Hz, 1 H), 7.56 (td, *J* = 7.4, 1.1 Hz, 1 H), 7.52–7.41 (m, 2 H), 7.36 (t, *J* = 7.4 Hz, 1 H), 7.20 (d, *J* = 7.4 Hz, 1 H), 6.94 (d, *J* = 8.4 Hz, 2 H), 6.31 (s, 1 H), 6.25 (d, *J* = 8.4 Hz, 2 H), 5.29 (s, 1 H), 4.94 (d, *J* = 4.8 Hz, 1 H), 4.44 (d, *J* = 4.6 Hz, 1 H), 3.89 (s, 3 H). ^{13}C NMR (151 MHz, $CDCl_3$): σ 184.3, 167.9, 143.8, 141.1, 140.2, 134.7, 134.0, 133.4, 132.9, 132.7, 131.8, 131.3 (2 C), 129.3, 128.5, 128.4, 128.1 (2 C), 127.7, 127.4, 127.3, 127.0, 58.1, 52.6, 46.6. MS (ESI+) *m/z* 425 ($[M + Na]^+$). HR MS (ESI+) calc. for $C_{25}H_{19}O_3ClNa$ $[M + Na]^+$ 425.0915, found 425.0915. IR (KBr): 3068, 3028, 2952, 1723, 1665, 1627, 1598, 1491, 1462, 1435, 1359, 1312, 1289, 1242, 1195, 1171, 1148, 1135, 1092, 1015, 964, 932, 847, 815, 785, 726, 689, 633 cm^{-1}. $[\alpha]_D$ = +54.0 (*c* = 1.0, $CHCl_3$). The enantiomeric excess (90%) was determined by HPLC with an IA column (*n*-heptane/*i*-PrOH 80:20, 1 $mLmin^{-1}$, λ = 190 nm): t_R = 5.8 (major), 6.4 (minor) min.

methyl (R)-2-((3-chlorophenyl)(10-oxo-9,10-dihydroanthracen-9-yl)methyl)acrylate (4f)

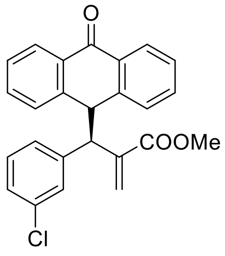

To a solution of anthrone (1 equiv, 49 mg, 0.25 mmol) in dichloromethane (0.25 mol/L) was added methyl 2-(((tert-butoxycarbonyl)oxy)(3-chlorophenyl)methyl)acrylate (2 equiv, 165 mg, 0.50 mmol), $(DHQD)_2AQN$ (20 mol%, 43 mg, 0.05 mmol). The reaction was stirred for 7 days at 0 °C. The reaction was followed

by NMR until the disappearance of starting material. The reaction mixture was concentrated in vacuo and purified by column chromatography (20:1 Hexane/EtOAc) to obtain 95 mg of desired product. The product yield is 94 %. ^{1}H NMR (600 MHz, $CDCl_3$): σ 8.18 (dd, *J* = 7.5, 1.6 Hz, 1 H), 8.00 (dd, *J* = 7.8, 1.1 Hz, 1 H), 7.60 (d, *J* = 7.3 Hz, 1 H), 7.56 (td, *J* = 7.4, 1.3 Hz, 1 H), 7.51–7.43 (m, 2 H), 7.37 (td, *J* = 7.5, 1.2 Hz, 1 H), 7.20 (dd, *J* = 7.3, 0.9 Hz, 1 H), 7.07 (m, 1 H), 6.90 (t, *J* = 7.9 Hz, 1 H), 6.33 (s, 1 H), 6.28 (t, *J* = 1.8 Hz, 1 H), 6.21 (d, *J* = 7.8 Hz, 1 H), 5.33 (d, *J* = 1.4 Hz, 1 H), 4.95 (d, *J* = 4.8 Hz, 1 H), 4.43 (d, *J* = 4.7 Hz, 1 H), 3.88 (s, 3 H). ^{13}C NMR (151 MHz, $CDCl_3$): σ 184.2, 167.8, 143.6, 141.0, 139.7, 138.1, 134.1, 133.73, 132.9, 132.7, 131.8, 130.2, 129.2, 129.1, 128.6, 128.4, 128.2, 127.8, 127.5, 127.4, 127.2, 127.0, 58.3, 52.6, 46.8. MS (ESI+) *m/z* 425 ([M + Na]$^+$). HR MS (ESI+) calc. for $C_{25}H_{19}O_3ClNa$ [M + Na]$^+$ 425.0915, found 425.0915. IR (KBr): 3075, 3063, 3025, 2947, 1712, 1673, 1622, 1598, 1568, 1461, 1434, 1359, 1314, 1266, 1213, 1177, 1156, 1138, 1084, 976, 955, 934, 917, 821, 806, 722, 710, 686, 635 cm^{-1}. $[\alpha]_D$ = +56.0 (*c* = 1.0, $CHCl_3$). The enantiomeric excess (94%) was determined by HPLC with an IA column (*n*-heptane/*i*-PrOH 80:20, 1 mLmin^{-1}, λ = 190 nm): t_R = 5.5 (minor), 6.1 (major) min.

<u>methyl (S)-2-((2-chlorophenyl)(10-oxo-9,10-dihydroanthracen-9-yl)methyl)acrylate</u> (4g)

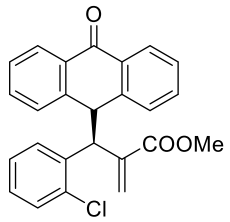

To a solution of anthrone (1 equiv, 49 mg, 0.25 mmol) in dichloromethane (0.25 mol/L) was added methyl 2-(((tert-butoxycarbonyl)oxy)(2-chlorophenyl)methyl)acrylate (2 equiv, 165 mg, 0.50 mmol), $(DHQD)_2AQN$ (20 mol%, 43 mg, 0.05 mmol). The reaction was stirred for 7 days at 0 °C. The reaction was followed by NMR until the disappearance of starting material. The reaction mixture was concentrated in vacuo and purified by column chromatography (20:1 Hexane/EtOAc) to obtain 96 mg of desired product. The product yield is 95 %. ^{1}H NMR (600 MHz, $CDCl_3$): σ 8.17 (d, *J* = 7.7 Hz, 1 H), 8.08 (d, *J* = 7.7 Hz, 1 H), 7.53–7.42 (m, 3 H), 7.41–7.30 (m, 3 H), 7.21 (d, *J* = 7.9 Hz, 1 H), 7.09 (td, *J* = 7.6, 1.3 Hz, 1 H), 6.96 (t, *J* = 7.6 Hz, 1 H), 6.45 (d, *J* = 7.8 Hz, 1 H), 6.29 (s, 1 H), 5.37 (d, *J* = 5.0 Hz, 1 H), 5.18 (s, 1 H), 5.06 (d, *J* = 5.1 Hz, 1 H), 3.79 (s, 3 H). ^{13}C NMR (151 MHz, $CDCl_3$) σ 184.7, 167.6, 142.5, 140.0, 139.1, 135.2, 134.7, 134.0, 133.2, 132.2, 132.0, 131.1, 130.0, 129.5, 129.1, 128.9, 128.6, 127.6, 127.5, 127.2, 126.9, 126.0, 52.5, 51.6, 45.8. MS (ESI+) *m/z* 425 ([M + Na]$^+$). HR MS (ESI+) calc. for

$C_{25}H_{19}O_3ClNa$ [M + Na]$^+$ 425.0915, found 425.0915. IR (KBr): 3071, 3031, 2997, 2950, 1715, 1662, 1624, 1600, 1474, 1462, 1439, 1313, 1288, 1255, 1219, 1189, 1134, 1091, 1038, 991, 962, 934, 814, 761, 753, 740, 705, 690, 667, 636 cm^{-1}. $[\alpha]_D$ = +59.0 (*c* = 1.0, $CHCl_3$). The enantiomeric excess (94%) was determined by HPLC with an IA column (*n*-heptane/*i*-PrOH 95:5, 1 mLmin^{-1}, λ = 190 nm): t_R = 7.9 (major), 8.5 (minor) min.

methyl (R)-2-((4-bromophenyl)(10-oxo-9,10-dihydroanthracen-9-yl)methyl)acrylate (4h)

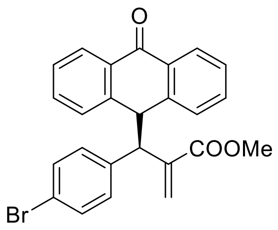

To a solution of anthrone (1 equiv, 49 mg, 0.25 mmol) in dichloromethane (0.25 mol/L) was added methyl 2-((4-bromophenyl)((tert-butoxycarbonyl)oxy)methyl)acrylate (2 equiv, 185 mg, 0.50 mmol), $(DHQD)_2AQN$ (20 mol%, 43 mg, 0.05 mmol). The reaction was stirred for 7 days at 0 °C. The reaction was followed by NMR until the disappearance of starting material. The reaction mixture was concentrated in vacuo and purified by column chromatography (20:1 Hexane/EtOAc) to obtain 106 mg of desired product. The product yield is 95 %. ^1H NMR (600 MHz, $CDCl_3$): σ 8.17 (dd, *J* = 7.7, 1.6 Hz, 1 H), 8.02 (dd, *J* = 7.7, 1.2 Hz, 1 H), 7.61 (d, *J* = 7.7 Hz, 1 H), 7.56 (td, *J* = 7.5, 1.4 Hz, 1 H), 7.51–7.42 (m, 2 H), 7.36 (td, *J* = 7.5, 1.1 Hz, 1 H), 7.20 (d, *J* = 7.4 Hz, 1 H), 7.14–7.07 (m, 2 H), 6.30 (s, 1 H), 6.23–6.17 (m, 2 H), 5.28 (d, *J* = 1.4 Hz, 1 H), 4.94 (d, *J* = 4.9 Hz, 1 H), 4.43 (d, *J* = 4.8 Hz, 1 H), 3.88 (s, 3 H). ^{13}C NMR (151 MHz, $CDCl_3$): σ 184.3, 167.9, 143.8, 141.1, 140.1, 135.3, 134.0, 132.9, 132.7, 131.8, 131.6 (2 C), 131.1 (2 C), 129.3, 128.5, 128.4, 127.8, 127.4, 127.3, 127.1, 121.5, 58.2, 52.6, 46.6. MS (ESI+) *m/z* 469 ([M + Na]$^+$). HR MS (ESI+) calc. for $C_{25}H_{19}O_3BrNa$ [M + Na]$^+$ 469.0410, found 469.0410. IR (KBr): 3065, 3026, 2950, 1716, 1668, 1600, 1488, 1462, 1437, 1407, 1360, 1314, 1293, 1245, 1191, 1171, 1138, 1075, 1010, 952, 932, 815, 767, 720, 688, 635 cm^{-1}. $[\alpha]_D$ = +56.0 (*c* = 1.0, $CHCl_3$). The enantiomeric excess (93%) was determined by HPLC with an IA column (*n*-heptane/*i*-PrOH 80:20, 1 mLmin^{-1}, λ = 196 nm): t_R = 6.0 (minor), 6.6 (major) min.

methyl (R)-2-((3-bromophenyl)(10-oxo-9,10-dihydroanthracen-9-yl)methyl)acrylate (4i)

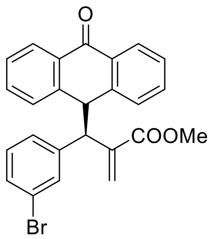

To a solution of anthrone (1 equiv, 49 mg, 0.25 mmol) in dichloromethane (0.25 mol/L) was added methyl 2-((3-bromophenyl)((tert-butoxycarbonyl)oxy)methyl)acrylate (2 equiv, 185 mg, 0.50 mmol), $(DHQD)_2AQN$ (20 mol%, 43 mg, 0.05 mmol). The reaction was stirred for 7 days at 0 °C. The reaction was followed by NMR until the disappearance of starting material. The reaction mixture was concentrated in vacuo and purified by column chromatography (20:1 Hexane/EtOAc) to obtain 91 mg of desired product. The product yield is 81 %. ^1H NMR (600 MHz, $CDCl_3$): σ 8.19 (dd, *J* = 7.7, 1.6 Hz, 1 H), 8.00 (dd, *J* = 7.7, 1.1 Hz, 1 H), 7.60 (d, *J* = 7.2 Hz, 1 H), 7.57 (td, *J* = 7.4, 1.4 Hz, 1 H), 7.45–7.51 (m, 2 H), 7.42–7.32 (m, 1 H), 7.23 (m, 1 H), 7.20 (dd, *J* = 7.4, 0.9 Hz, 1 H), 6.84 (t, *J* = 7.8 Hz, 1 H), 6.41 (t, *J* = 1.7 Hz, 1 H), 6.33 (s, 1 H), 6.26 (d, *J* = 7.8 Hz, 1 H), 5.32 (d, *J* = 1.4 Hz, 1 H), 4.94 (d, *J* = 4.8 Hz, 1 H), 4.42 (d, *J* = 4.7 Hz, 1 H), 3.89 (s, 3 H). ^{13}C NMR (151 MHz, $CDCl_3$): σ 184.2, 167.8, 143.6, 141.0, 139.7, 138.4, 134.1, 133.1, 132.9, 132.7, 131.8, 130.5, 129.4, 129.2, 128.6 (2 C), 128.4, 127.8, 127.4, 127.2, 126.9, 121.9, 58.3, 52.6, 46.8. MS (ESI+) *m/z* 469 ($[M + Na]^+$). HR MS (ESI+) calc. for $C_{25}H_{19}O_3BrNa$ $[M + Na]^+$ 469.0410, found 469.0411. IR (KBr): 3076, 3059, 3026, 2949, 1713, 1671, 1624, 1600, 1561, 1475, 1462, 1434, 1360, 1312, 1300, 1264, 1214, 1159, 1137, 955, 931, 816, 801, 718, 688, 634 cm^{-1}. $[\alpha]_D$ = –25.7 (*c* = 0.7, $CHCl_3$). The enantiomeric excess (68%) was determined by HPLC with an IA column (*n*-heptane/*i*-PrOH 80:20, 1 $mLmin^{-1}$, λ = 190 nm): t_R = 5.6 (major), 6.1 (minor) min.

methyl (S)-2-((2-bromophenyl)(10-oxo-9,10-dihydroanthracen-9-yl)methyl)acrylate (4j)

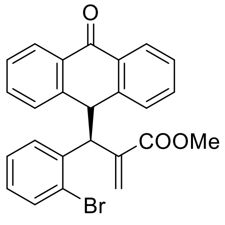

To a solution of anthrone (1 equiv, 49 mg, 0.25 mmol) in dichloromethane (0.25 mol/L) was added methyl 2-((2-bromophenyl)((tert-butoxycarbonyl)oxy)methyl)acrylate (2 equiv, 185 mg, 0.50 mmol), $(DHQD)_2AQN$ (20 mol%, 43 mg, 0.05 mmol). The reaction was stirred for 7 days at 0 °C. The reaction was followed by NMR until the disappearance of starting material. The reaction mixture was concentrated in vacuo and purified by column chromatography (20:1 Hexane/EtOAc) to obtain 105 mg of desired product. The product yield is 94 %. ^1H

NMR (600 MHz, $CDCl_3$): σ 8.18 (d, *J* = 7.6 Hz, 1 H), 8.09 (d, *J* = 7.6 Hz, 1 H), 7.58–7.36 (m, 6 H), 7.32 (d, *J* = 7.7 Hz, 1 H), 7.01 (m, 2 H), 6.48 (m, 1 H), 6.28 (s, 1 H), 5.35 (d, *J* = 5.2 Hz, 1 H), 5.19 (s, 1 H), 5.06 (d, *J* = 5.3 Hz, 1 H), 3.79 (s, 3 H). ^{13}C NMR (151 MHz, $CDCl_3$): σ 184.82, 167.59, 142.33, 142.01, 139.19, 136.48, 134.05, 133.46, 133.24, 132.22, 132.04, 131.18, 129.68, 129.07, 128.89 (2 C), 127.65, 127.54, 127.22, 126.97, 126.65, 126.44, 54.43, 52.49, 45.79. MS (ESI+) *m/z* 469 ($[M + Na]^+$). HR MS (ESI+) calc. for $C_{25}H_{19}O_3BrNa$ $[M + Na]^+$ 469.0410, found 469.0410. IR (KBr): 3068, 3032, 3010, 2946, 1715, 1670, 1601, 1467, 1436, 1361, 1315, 1295, 1261, 1194, 1160, 1138, 1021, 955, 944, 932, 813, 789, 758, 737, 717, 689, 661, 636 cm^{-1}. $[\alpha]_D$ = +59.0 (*c* = 1.0, $CHCl_3$). The enantiomeric excess (94%) was determined by HPLC with an IA column (*n*-heptane/*i*-PrOH 90:10, 1 $mLmin^{-1}$, λ = 190 nm): t_R = 6.4 (major), 7.0 (minor) min.

<u>methyl (S)-2-((10-oxo-9,10-dihydroanthracen-9-yl)(thiophen-2-yl)methyl)acrylate</u> (4k)

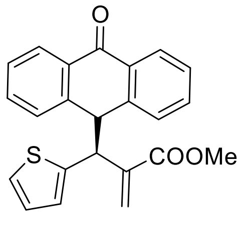

To a solution of anthrone (1 equiv, 49 mg, 0.25 mmol) in dichloromethane (0.25 mol/L) was added methyl 2-(((tert-butoxycarbonyl)oxy)(thiophen-2-yl)methyl)acrylate (2 equiv, 149 mg, 0.50 mmol), $(DHQD)_2AQN$ (20 mol%, 43 mg, 0.05 mmol). The reaction was stirred for 7 days at 0 °C. The reaction was followed by NMR until the disappearance of starting material. The reaction mixture was concentrated in vacuo and purified by column chromatography (20:1 Hexane/EtOAc) to obtain 87 mg of desired product. The product yield is 93 %. ^1H NMR (600 MHz, $CDCl_3$): σ 8.19 (dd, *J* = 7.6, 1.5 Hz, 1 H), 8.04 (dd, *J* = 7.8, 1.2 Hz, 1 H), 7.63 (d, *J* = 7.7 Hz, 1 H), 7.57 (td, *J* = 7.5, 1.4 Hz, 1 H), 7.53–7.45 (m, 2 H), 7.38 (dt, *J* = 7.5, 1.1 Hz, 1 H), 7.25 (m, 1 H), 6.99 (dd, *J* = 5.1, 0.9 Hz, 1 H), 6.70 (dd, *J* = 5.1, 3.6 Hz, 1 H), 6.38 (s, 1 H), 5.90 (d, *J* = 3.4 Hz, 1 H), 5.37 (d, *J* = 1.1 Hz, 1 H), 4.88 (d, *J* = 4.3 Hz, 1 H), 4.85 (d, *J* = 4.2 Hz, 1 H), 3.89 (s, 3 H). ^{13}C NMR (151 MHz, $CDCl_3$): σ 184.5, 167.6, 143.6, 140.5, 139.6, 138.2, 134.4, 133.1, 132.8, 131.5, 129.7, 129.5, 128.4, 127.8, 127.5, 127.4, 127.0, 126.9, 126.7, 124.6, 52.9, 52.6, 47.6. MS (ESI+) *m/z* 397 ($[M + Na]^+$). HR MS (ESI+) calc. for $C_{23}H_{18}O_3SNa$ $[M + Na]^+$ 397.0869, found 397.0870. IR (KBr): 3069, 3030, 2956, 1709, 1666, 1627, 1600, 1463, 1477, 1443, 1314, 1301, 1275, 1264, 1211, 1196, 1170, 1146, 1091, 1045, 988, 951, 933, 846, 817, 771, 702, 635, 569 cm^{-1}. $[\alpha]_D$ = +73.0 (*c* = 1.0, $CHCl_3$). The

enantiomeric excess (90%) was determined by HPLC with an IA column (*n*-heptane/*i*-PrOH 80:20, 1 mLmin^{-1}, λ = 190 nm): t_R = 5.7 (minor), 7.0 (major) min.

methyl (S)-2-methylene-3-(10-oxo-9,10-dihydroanthracen-9-yl)-5-phenylpentanoate (4l)

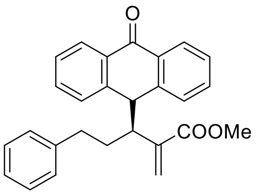

To a solution of anthrone (1equiv, 19 mg, 0.1 mmol) in dichloromethane (0.1 mol/L) was added methyl 3-((tert-butoxycarbonyl)oxy)-2-methylene-5-phenylpentanoate (2 equiv, 65 mg, 0.2 mmol), $(DHQD)_2AQN$ (20 mol%, 14 mg, 0.02 mmol). The reaction was stirred for 5 days at 0 °C. The reaction was followed by NMR until the disappearance of starting material. The reaction mixture was purified by column chromatography (20:1 Hexane/EtOAc) to obtain 20 mg of desired product. The product yield is 55 %. ^{1}H NMR (400 MHz, $CDCl_3$) σ 8.21 – 8.10 (m, 2H), 7.53 – 7.30 (m, 5H), 7.20 (ddd, *J* = 12.2, 7.2, 2.3 Hz, 2H), 7.13 – 7.00 (m, 4H), 6.77 (d, *J* = 6.7 Hz, 2H), 6.26 (s, 1H), 4.83 (s, 1H), 4.31 (d, *J* = 3.5 Hz, 1H), 3.62 (s, 3H), 3.22 (ddd, *J* = 11.4, 3.5, 3.3 Hz, 1H), 2.35 (ddd, *J* = 14.1, 9.6, 4.5 Hz, 1H), 2.24 – 2.12 (m, 1H), 1.53 (m, 1H), 1.25 – 1.16 (m, 1H). ^{13}C NMR (101 MHz, $CDCl_3$) σ 185.1 (q), 167.6 (q), 143.1 (q), 141.7 (q), 141.2 (q), 138.9 (q), 133.5 (q), 133.1 (q), 132.5, 131.7, 129.3, 128.7, 128.3, 128.1, 127.5 (CH_2), 127.3, 127.2, 127.1, 126.7, 125.9, 52.0, 49.0, 46.6, 33.1 (CH_2), 29.8 (CH_2). HR-MS (m/z) for $C_{27}H_{24}O_3$ calculated $[M+H]^+$ 397.1798, measured $[M+H]^+$ 397.1803. IR (cm^{-1}): 3061 (=C-H, stretch), 3026 (=C-H, stretch), 2947 (C-H, stretch), 1715 (C=O, stretch), 1665 (C=O, stretch), 1600 (C=C, stretch), 1314 (C-O ester, stretch). $[\alpha]_D^{19}$ = +2.1 (c = 0.01 g/ml, $CHCl_3$). The enantiomeric excess was determined by HPLC using a Chiralpak IC column [hexane/*i*PrOH = 90:10]; flow rate 1.0 mL/min; 254 nm; t_{r1}= 21.4, t_{r2} = 43.5; 4 % ee.

(R)-2-((10-oxo-9,10-dihydroanthracen-9-yl)(phenyl)methyl)acrylonitrile (4m)

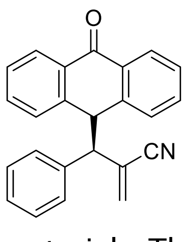

To a solution of anthrone (1 equiv, 19 mg, 0.1 mmol) in dichloromethane (0.1 mol/L) was added tert-butyl (2-cyano-1-phenylallyl) carbonate (2 equiv, 50 mg, 0.2 mmol), $(DHQD)_2AQN$ (20 mol%, 14 mg, 0.02 mmol). The reaction was stirred for 5 days at 0 °C. The reaction was followed by NMR until the disappearance of starting material. The reaction mixture was purified by column chromatography (20:1

Hexane/EtOAc) to obtain 26 mg of desired product. The product yield is 80 %. ^{1}H NMR (400 MHz, $CDCl_3$) σ 8.08 (ddd, *J* = 23.2, 7.7, 1.4 Hz, 2H), 7.61 (ddd, *J* = 14.1, 9.0, 4.5 Hz, 2H), 7.41 (ddd, *J* = 15.3, 7.8, 1.3 Hz, 2H), 7.35 (m, 1H), 7.23 – 7.14 (m, 2H), 7.07 (dd, *J* = 10.5, 4.8 Hz, 2H), 6.55 (d, *J* = 7.2 Hz, 2H), 5.86 (d, *J* = 0.9 Hz, 1H), 5.41 (d, *J* = 1.4 Hz, 1H), 4.90 (d, *J* = 6.8 Hz, 1H), 3.64 (d, *J* = 6.8 Hz, 1H). ^{13}C NMR (101 MHz, $CDCl_3$) σ 184.4 (q), 142.7 (q), 140.8 (q), 134.9 (q), 133.6 (CH_2), 133.5 (q), 132.8 (q), 132.6, 131.8, 129.1, 128.7, 128.4, 128.3, 128.1, 127.8, 127.7, 127.4, 127.3, 124.2(q), 118.9 (q), 63.2, 47.8. HR-MS (m/z) for $C_{24}H_{17}NO$ calculated $[M+H]^+$ 336.1383, measured $[M+H]^+$ 336.1379. IR (cm^{-1}): 3064 (=C-H, stretch), 3030 (=C-H, stretch), 2221 (C≡N, stretch), 1665 (C=O, stretch), 1600 (C=C, stretch). $[\alpha]_D^{19}$ = +1.7 (c = 0.013 g/ml, $CHCl_3$). The enantiomeric excess was determined by HPLC using a Chiralpak ID column [hexane/*i*PrOH = 90:10]; flow rate 1.0 mL/min; 254 nm; t_{r1}= 16.7, t_{r2} = 18.0; 63 % ee.

<u>(R)-2-((10-oxo-9,10-dihydroanthracen-9-yl)(p-tolyl)methyl)acrylonitrile</u> (4n)

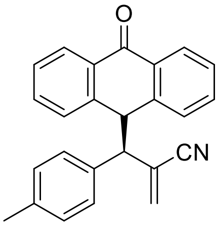

To a solution of anthrone (1 equiv, 19 mg, 0.1 mmol) in dichloromethane (0.1 mol/L) was added tert-butyl (2-cyano-1-(p-tolyl)allyl) carbonate (2 equiv, 55 mg, 0.2 mmol), $(DHQD)_2AQN$ (20 mol%, 14 mg, 0.02 mmol). The reaction was stirred for 5 days at 0 °C. The reaction was followed by NMR until the disappearance of starting material. The reaction mixture was purified by column chromatography (20:1 Hexane/EtOAc) to obtain 32 mg of desired product. The product yield is 92 %. ^{1}H NMR (400 MHz, $CDCl_3$) σ 8.09 (ddd, *J* = 21.8, 7.7, 1.4 Hz, 2H), 7.65 (d, *J* = 7.3 Hz, 1H), 7.59 (ddd, *J* = 8.5, 7.5, 1.2 Hz, 1H), 7.39 (m, 3H), 7.24 – 7.17 (m, 1H), 6.88 (d, *J* = 7.9 Hz, 2H), 6.45 (d, *J* = 8.1 Hz, 2H), 5.83 (d, *J* = 0.6 Hz, 1H), 5.37 (d, *J* = 1.4 Hz, 1H), 4.87 (d, *J* = 6.8 Hz, 1H), 3.61 (d, *J* = 6.8 Hz, 1H), 2.24 (s, 3H). ^{13}C NMR (101 MHz, $CDCl_3$) σ 184.5 (q), 142.8 (q), 140.9 (q), 137.8 (q), 133.5 (q), 133.4 (CH_2), 132.8 (q), 132.5, 131.9 (q), 131.8, 129.0, 128.9, 128.8, 128.5, 127.7, 127.7, 127.4, 127.3, 124.5 (q), 119.0 (q), 62.9, 47.7, 21.1. HR-MS (m/z) for $C_{25}H_{19}NO$ calculated $[M+H]^+$ 350.1539, measured $[M+H]^+$ 350.1547. IR (cm^{-1}): 3064 (=C-H, stretch), 3024 (=C-H, stretch), 2220 (C≡N, stretch), 1666 (C=O, stretch), 1600 (C=C, stretch). $[\alpha]_D^{19}$ = +4.9 (c = 0.016 g/ml, $CHCl_3$). The enantiomeric excess was determined by HPLC using a Chiralpak ID column [hexane/*i*PrOH = 95:5]; flow rate 1.0 mL/min; 254 nm; t_{r1}= 21.6, t_{r2} = 23.6; 68 % ee.

(R)-10-(2-methylene-3-oxo-1-phenylbutyl)anthracen-9(10H)-one (4o)

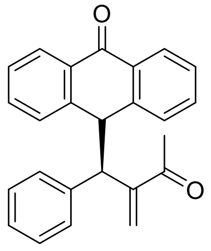

To a solution of anthrone (1 equiv, 19 mg, 0.1 mmol) in dichloromethane (0.1 mol/L) was added tert-butyl (2-methylene-3-oxo-1-phenylbutyl) carbonate (2 equiv, 55 mg, 0.2 mmol), $(DHQD)_2AQN$ (20 mol%, 14 mg, 0.02 mmol). The reaction was stirred for 5 days at 0 °C. The reaction was followed by NMR until the disappearance of starting material. The reaction mixture was purified by column chromatography (20:1 Hexane/EtOAc) to obtain 31 mg of desired product. The product yield is 88 %. ^{1}H NMR (400 MHz, $CDCl_3$) σ 8.15 (dd, *J* = 7.5, 1.6 Hz, 1H), 8.00 (d, *J* = 7.7 Hz, 1H), 7.55 – 7.39 (m, 4H), 7.34 (ddd, *J* = 8.2, 5.9, 2.6 Hz, 1H), 7.20 – 7.15 (m, 1H), 7.11 (dd, *J* = 7.3 Hz, 1H), 7.02 (dd, *J* = 7.5, 7.3 Hz, 2H), 6.47 (d, *J* = 7.4, 7.2 Hz, 2H), 6.19 (s, 1H), 5.51 (d, *J* = 0.8 Hz, 1H), 4.92 (d, *J* = 4.9 Hz, 1H), 4.62 (d, *J* = 4.8 Hz, 1H), 2.39 (s, 3H). ^{13}C NMR (101 MHz, $CDCl_3$) σ 199.7 (q), 184.5 (q), 147.7 (q), 143.7 (q), 141.9 (q), 136.9 (q), 133.9 (q), 133.0 (q), 132.2, 131.5, 129.9, 129.2 (CH_2), 129.0, 128.5, 127.9, 127.4, 127.1, 127.08, 127.0, 126.6, 56.3, 46.9, 26.5. HR-MS (m/z) for $C_{25}H_{20}O_2$ calculated $[M+H]^+$ 353.1536, measured $[M+H]^+$ 353.1540. IR (cm^{-1}): 3064 (=C-H, stretch), 3029 (=C-H, stretch), 1665 (C=O, stretch), 1600 (C=C, stretch). $[\alpha]_D^{19}$ = +87.2 (c = 0.015 g/ml, $CHCl_3$). The enantiomeric excess was determined by HPLC using a Chiralpak ID column [hexane/*i*PrOH = 90:10]; flow rate 1.0 mL/min; 254 nm; t_{r1}= 28.0, t_{r2} = 31.6; 88 % ee.

(R)-10-(2-methylene-3-oxo-1-(p-tolyl)butyl)anthracen-9(10H)-one (4p)

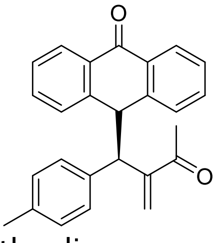

To a solution of anthrone (1 equiv, 19 mg, 0.1 mmol) in dichloromethane (0.1 mol/L) was added tert-butyl (2-methylene-3-oxo-1-(p-tolyl)butyl) carbonate (2 equiv, 58 mg, 0.2 mmol), $(DHQD)_2AQN$ (20 mol%, 14 mg, 0.02 mmol). The reaction was stirred for 5 days at 0 °C. The reaction was followed by NMR until the disappearance of starting material. The reaction mixture was purified by column chromatography (20:1 Hexane/EtOAc) to obtain 33 mg of desired product. The product yield is 92 %. ^{1}H NMR (400 MHz, $CDCl_3$) σ 8.16 (dd, *J* = 7.5, 1.6 Hz, 1H), 8.01 (d, *J* = 7.6 Hz, 1H), 7.57 – 7.39 (m, 4H), 7.34 (s, 1H), 7.19 (d, *J* = 7.4 Hz, 1H), 6.83 (d, *J* = 7.9 Hz, 2H), 6.36 (d, *J* = 8.1 Hz, 2H), 6.17 (s, 1H), 5.47 (s, 1H), 4.89 (d, *J* = 4.9 Hz, 1H), 4.60 (d, *J* = 4.8 Hz, 1H), 2.38 (s, 3H), 2.21 (s, 3H). ^{13}C NMR (101

MHz, $CDCl_3$) σ 199.7 (q), 184.6 (q), 148.0 (q), 143.9 (q), 142.1 (q), 136.7 (q), 133.9 (q), 133.9 (q), 133.0 (q), 132.2, 131.5, 129.7, 129.1 (CH_2), 129.0, 128.6, 128.6, 127.3, 127.0, 127.0, 126.7, 55.9, 46.9, 26.5, 21.0. HR-MS (m/z) for $C_{26}H_{22}O_2$ calculated $[M+H]^+$ 367.1693, measured $[M+H]^+$ 367.1690. IR (cm^{-1}): 3064 (=C-H, stretch), 3027 (=C-H, stretch), 1665 (C=O, stretch), 1600 (C=C, stretch). $[\alpha]_D^{19}$ = +32.0 (c = 0.016 g/ml, $CHCl_3$). The enantiomeric excess was determined by HPLC using a Chiralpak IA column [hexane/*i*PrOH = 90:10]; flow rate 1.0 mL/min; 254 nm; t_{r1}= 8.5, t_{r2} = 12.6; 86 % ee.

<u>(R)-10-(2-methylene-1-(4-nitrophenyl)-3-oxobutyl)anthracen-9(10H)-one</u> (4q)

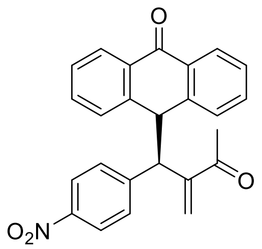

To a solution of anthrone (1 equiv, 19 mg, 0.1 mmol) in dichloromethane (0.1 mol/L) was added tert-butyl (2-methylene-1-(4-nitrophenyl)-3-oxobutyl) carbonate (2 equiv, 64 mg, 0.2 mmol), $(DHQD)_2AQN$ (20 mol%, 14 mg, 0.02 mmol). The reaction was stirred for 5 days at 0 °C. The reaction was followed by NMR until the disappearance of starting material. The reaction mixture was purified by column chromatography (20:1 Hexane/EtOAc) to obtain 33 mg of desired product. The product yield is 92 %. ^1H NMR (400 MHz, $CDCl_3$) σ 8.23 – 8.09 (m, 1H), 8.02 (d, *J* = 7.7 Hz, 1H), 7.88 (d, *J* = 8.8 Hz, 2H), 7.58 – 7.50 (m, 2H), 7.50 – 7.42 (m, 2H), 7.41 – 7.33 (m, 1H), 7.19 – 7.08 (m, 1H), 6.68 (d, *J* = 8.8 Hz, 2H), 6.23 (s, 1H), 5.47 (s, 1H), 4.96 (d, *J* = 5.4 Hz, 1H), 4.66 (d, *J* = 5.3 Hz, 1H), 2.41 (s, 3H). ^{13}C NMR (101 MHz, $CDCl_3$) σ 199.2 (q), 184.3 (q), 146.9 (q), 145.0 (q), 143.0 (q), 141.1 (q), 133.7 (q), 132.8 (q), 132.6, 131.8 (q), 130.6, 129.4 (CH_2), 128.9, 128.3, 127.8, 127.6, 127.4, 127.1, 123.0, 56.9, 46.5, 26.5. HR-MS (m/z) for $C_{25}H_{19}NO_4$ calculated $[M+H]^+$ 398.1387, measured $[M+H]^+$ 398.1391. IR (cm^{-1}): 3071 (=C-H, stretch), 3029 (=C-H, stretch), 1665 (C=O, stretch), 1600 (C=C, stretch), 1520 (N-O, asymmetric stretch), 1346 (N-O, symmetric stretch). $[\alpha]_D^{19}$ = +51.0 (c = 0.016 g/ml, $CHCl_3$). The enantiomeric excess was determined by HPLC using a Chiralpak IE column [hexane/*i*PrOH = 80:20]; flow rate 1.0 mL/min; 254 nm; t_{r1}= 37.1, t_{r2} = 41.5; 85 % ee.

(R)-10-(1-(4-bromophenyl)-2-methylene-3-oxobutyl)anthracen-9(10H)-one (5r)

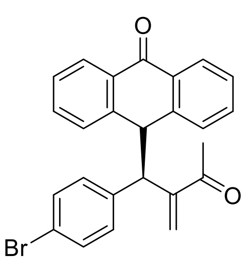

To a solution of anthrone (1 equiv, 19 mg, 0.1 mmol) in dichloromethane (0.1 mol/L) was added 1-(4-bromophenyl)-2-methylene-3-oxobutyl tert-butyl carbonate (2 equiv, 71 mg, 0.2 mmol), $(DHQD)_2AQN$ (20 mol%, 14 mg, 0.02 mmol). The reaction was stirred for 5 days at 0 °C. The reaction was followed by NMR until the disappearance of starting material. The reaction mixture was purified by column chromatography (20:1 Hexane/EtOAc) to obtain 35 mg of desired product. The product yield is 83 %. ^{1}H NMR (400 MHz, $CDCl_3$) σ 8.17 (dd, *J* = 7.4, 1.7 Hz, 1H), 8.04 (d, *J* = 7.7 Hz, 1H), 7.50 (dd, *J* = 7.7, 2.5 Hz, 2H), 7.48 – 7.41 (m, 2H), 7.37 (ddd, *J* = 8.2, 5.2, 3.3 Hz, 1H), 7.20 – 7.10 (m, 3H), 6.38 (d, *J* = 8.4 Hz, 2H), 6.18 (s, 1H), 5.43 (s, 1H), 4.90 (d, *J* = 5.1 Hz, 1H), 4.55 (d, *J* = 5.1 Hz, 1H), 2.38 (s, 3H). ^{13}C NMR (101 MHz, $CDCl_3$) σ 199.5 (q), 184.4 (q), 147.5 (q), 143.4 (q), 141.7 (q), 136.3 (q), 133.7 (q), 132.9 (q), 132.3, 131.7, 131.4, 131.1, 129.2 (CH_2), 129.0, 128.5, 127.5, 127.3, 127.2, 126.9, 121.2 (q), 56.1, 46.6, 26.5. HR-MS (m/z) for $C_{25}H_{19}BrO_2$ calculated $[M+H]^+$ 431.0641, measured $[M+H]^+$ 431.0635. IR (cm^{-1}): 3066 (=C-H, stretch), 3027 (=C-H, stretch), 1665 (C=O, stretch), 1600 (C=C, stretch). $[\alpha]_D^{19}$ = +51.1 (c = 0.017 g/ml, $CHCl_3$). The enantiomeric excess was determined by HPLC using a Chiralpak IC column [hexane/*i*PrOH = 95:5]; flow rate 1.0 mL/min; 254 nm; t_{r1}= 24.0, t_{r2} = 27.2; 92 % ee.

(R)-10-(1-(4-chlorophenyl)-2-methylene-3-oxobutyl)anthracen-9(10H)-one (4s)

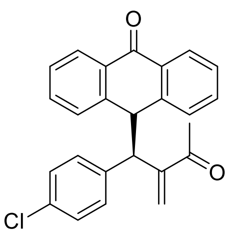

To a solution of anthrone (1equiv, 19 mg, 0.1 mmol) in dichloromethane (0.1 mol/L) was added tert-butyl (1-(4-chlorophenyl)-2-methylene-3-oxobutyl) carbonate (2 equiv, 62 mg, 0.2 mmol), $(DHQD)_2AQN$ (20 mol%, 14 mg, 0.02 mmol). The reaction was stirred for 5 days at 0 °C. The reaction was followed by NMR until the disappearance of starting material. The reaction mixture was purified by column chromatography (20:1 Hexane/EtOAc) to obtain 29 mg of desired product. The product yield is 76 %. ^{1}H NMR (400 MHz, $CDCl_3$) σ 8.17 (dd, *J* = 7.4, 1.7 Hz, 1H), 8.03 (d, *J* = 7.7 Hz, 1H), 7.52 (d, *J* = 3.7 Hz, 2H), 7.45 (m, 2H), 7.37 (m, 1H), 7.19 – 7.12 (m, 1H), 7.04 – 6.96 (m, 2H), 6.42 (d, *J* = 8.5 Hz, 2H), 6.18 (s, 1H), 5.43 (s, 1H), 4.90 (d, *J* = 5.2 Hz, 1H), 4.56 (d, *J* = 5.0 Hz, 1H), 2.39 (s, 3H). ^{13}C NMR (101 MHz, $CDCl_3$) σ 199.5 (q), 184.4 (q), 147.6 (q), 143.5 (q), 141.7

(q), 135.7 (q), 133.8 (q), 133.0 (q), 132.9 (q), 132.4, 131.6, 131.1, 129.2 (CH_2), 129.0, 128.6, 128.4, 128.1, 127.5, 127.3, 127.2, 126.9, 56.1, 46.7, 26.5. HR-MS (m/z) for $C_{25}H_{19}ClO_2$ calculated $[M+H]^+$ 387.1146, measured $[M+H]^+$ 387.1150. IR (cm^{-1}): 3067 (=C-H, stretch), 3027 (=C-H, stretch), 1665 (C=O, stretch), 1600 (C=C, stretch). $[\alpha]_D^{19}$ = +68.4 (c = 0.015 g/ml, $CHCl_3$). The enantiomeric excess was determined by HPLC using a Chiralpak ID column [hexane/*i*PrOH = 90:10]; flow rate 1.0 mL/min; 254 nm; t_{r1}= 24.0, t_{r2} = 29.4; 90 % ee.

(R)-4-(2-methylene-3-oxo-1-(10-oxo-9,10-dihydroanthracen-9-yl)butyl)benzonitrile (4t)

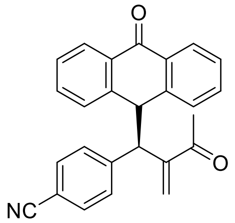

To a solution of anthrone (1equiv, 19 mg, 0.1 mmol) in dichloromethane (0.1 mol/L) was added tert-butyl (1-(4-cyanophenyl)-2-methylene-3-oxobutyl) carbonate (2 equiv, 60 mg, 0.2 mmol), $(DHQD)_2AQN$ (20 mol%, 14 mg, 0.02 mmol). The reaction was stirred for 5 days at 0 °C. The reaction was followed by NMR until the disappearance of starting material. The reaction mixture was purified by column chromatography (20:1 Hexane/EtOAc) to obtain 32 mg of desired product. The product yield is 85 %. ^1H NMR (400 MHz, $CDCl_3$) σ 8.20 – 8.13 (m, 1H), 8.03 (d, *J* = 7.5 Hz, 1H), 7.56 – 7.42 (m, 4H), 7.41 – 7.35 (m, 1H), 7.32 (d, *J* = 8.3 Hz, 2H), 7.16 – 7.08 (m, 1H), 6.63 (d, *J* = 8.3 Hz, 2H), 6.21 (s, 1H), 5.45 (s, 1H), 4.94 (d, *J* = 5.4 Hz, 1H), 4.59 (d, *J* = 5.3 Hz, 1H), 2.40 (s, 3H). ^{13}C NMR (101 MHz, $CDCl_3$) σ 199.3 (q), 184.3 (q), 146.9 (q), 143.1 (q), 142.9 (q), 141.2 (q), 133.7 (q), 132.8 (q), 132.5, 131.8, 131.7, 130.5, 129.3 (CH_2), 129.0, 128.3, 127.8, 127.5, 127.3, 127.1, 118.5 (q), 111.1 (q), 57.1, 46.5, 26.5. HR-MS (m/z) for $C_{26}H_{19}NO_2$ calculated $[M+H]^+$ 378.1489, measured $[M+H]^+$ 378.1483. IR (cm^{-1}): 3068 (=C-H, stretch), 3029 (=C-H, stretch), 2228 (C≡N, stretch), 1665 (C=O, stretch), 1600 (C=O, stretch). $[\alpha]_D^{19}$ = +46.2 (c = 0.016 g/ml, $CHCl_3$). The enantiomeric excess was determined by HPLC using a Chiralpak IE column [hexane/*i*PrOH = 70:30]; flow rate 0.5 mL/min; 254 nm; t_{r1}= 52.2, t_{r2} = 54.0; 79 % ee.

(R)-10-(2-methylene-3-oxo-1-phenylpentyl)anthracen-9(10H)-one (4u)

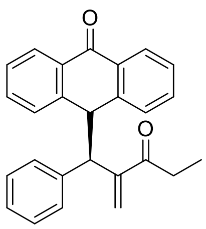

To a solution of anthrone (1 equiv, 19 mg, 0.1 mmol) in dichloromethane (0.1 mol/L) was added tert-butyl (2-methylene-3-oxo-1-phenylpentyl) carbonate (2 equiv, 58 mg, 0.2 mmol), $(DHQD)_2AQN$ (20 mol%, 14 mg, 0.02 mmol). The reaction was stirred for 5 days at 0 °C. The reaction was followed by NMR until the disappearance of starting material. The reaction mixture was purified by column chromatography (20:1 Hexane/EtOAc) to obtain 29 mg of desired product. The product yield is 81 %. ^1H NMR (400 MHz, $CDCl_3$) σ 8.15 (dd, *J* = 7.5, 1.5 Hz, 1H), 8.00 (d, *J* = 7.6 Hz, 1H), 7.60 – 7.38 (m, 5H), 7.38 – 7.31 (m, 1H), 7.18 (d, *J* = 8.0 Hz, 1H), 7.11 (dd, *J* = 7.3, 7.2 Hz, 1H), 7.02 (dd, *J* = 7.5, 7.3 Hz, 2H), 6.48 (d, *J* = 7.6 Hz, 2H), 6.18 (s, 1H), 5.46 (s, 1H), 4.92 (d, *J* = 4.9 Hz, 1H), 4.64 (d, *J* = 4.8 Hz, 1H), 2.81 (dt, *J* = 10.9, 7.3 Hz, 1H), 2.69 (dt, *J* = 10.1, 7.3 Hz, 1H), 1.12 (t, *J* = 7.3 Hz, 3H). ^{13}C NMR (101 MHz, $CDCl_3$) σ 202.5 (q), 184.5 (q), 147.3 (q), 143.8 (q), 142.0 (q), 137.1 (q), 133.9 (q), 133.0 (q), 132.2, 131.5, 129.9, 129.0, 128.6, 127.9, 127.8 (CH_2), 127.3, 127.1, 127.0, 127.0, 126.6, 56.7, 47.0, 31.4, 8.5. HR-MS (m/z) for $C_{26}H_{22}O_2$ calculated $[M+H]^+$ 367.1693, measured $[M+H]^+$ 367.1701. IR (cm^{-1}): 3064 (=C-H, stretch), 3027 (=C-H, stretch), 2978 (C-H, stretch), 2937 (C-H, stretch), 1716 (C=O, stretch), 1665 (C=O, stretch), 1600 (C=C, stretch). $[\alpha]_D^{19}$ = +47.2 (c = 0.015 g/ml, $CHCl_3$). The enantiomeric excess was determined by HPLC using a Chiralpak ID column [hexane/*i*PrOH = 90:10]; flow rate 1.0 mL/min; 254 nm; t_{r1}= 22.7, t_{r2} = 28.4; 98 % ee.

Hydrogenation reaction

methyl (2S,3S)-3-(4-fluorophenyl)-2-methyl-3-(10-oxo-9,10-dihydroanthracen-9-yl)propanoate (5b)

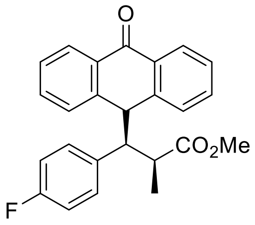

In a 2-neck round-bottom flask (RBF) methyl (R)-2-((4-fluorophenyl)(10-oxo-9,10-dihydroanthracen-9-yl)methyl)acrylate (4b) (1.0 equiv, 20 mg, 0.052 mmol) was added together with Pd/C (0.15 equiv w/w, 3 mg). EtOAc (1 ml, 0.052 mol/L) was added. The RBF was sealed and flushed twice with Argon. Then the reaction mixture was flushed once with H_2. The rubber balloon was refilled with H_2 and connected to the RBF. The reaction was monitored by TLC until the reaction completion (3 days). The mixture was filtered through Celite, washed with EtOAc and concentrated in vacuo. The mixture was purified by Column chromatography (10:1 Hexane:EtOAc) to obtain 18 mg of desired product. The product yield is 90 %. ^{1}H NMR (400 MHz, $CDCl_3$) σ 8.01 (dd, *J* = 8.0, 1.3 Hz, 1H), 7.92 (d, *J* = 6.9 Hz, 1H), 7.62 (ddd, *J* = 11.7, 9.5, 4.3 Hz, 3H), 7.47 (t, *J* = 7.0 Hz, 2H), 7.43 – 7.35 (m, 1H), 6.46 (t, *J* = 8.7 Hz, 2H), 5.76 (dd, *J* = 8.6, 5.4 Hz, 2H), 4.74 (d, *J* = 3.0 Hz, 1H), 3.30 – 3.23 (dd, J = 11.3, 3.1 Hz, 1H), 3.26 (s, 3H), 3.15 - 3.05 (m, 1H), 1.79 (d, *J* = 6.7 Hz, 3H). ^{19}F NMR (376 MHz, $CDCl_3$) σ -114.79. ^{13}C NMR (101 MHz, $CDCl_3$) σ 183.1 (q), 175.3 (q), 144.2 (q), 140.0 (q), 134.7 (q), 133.6 (q), 132.7, 131.9, 131.2 (q), 131.2 (q), 127.9, 127.9, 127.7, 127.2, 127.1, 126.4, 114.1, 113.9, 59.0, 51.5, 43.8, 42.2, 17.3. HR-MS (m/z) for $C_{25}H_{21}FO_3$ calculated $[M+H]^+$ 389.1547, measured $[M+H]^+$ 389.1544. IR (cm^{-1}): 1735 (C=O, stretch), 1668 (C=O, stretch), 1314 (C-O ester, stretch), 1166 (C-F, stretch), 931, 836, 754, 722, 693. $[\alpha]_D^{21}$ = -6.1 (c = 0.006 g/ml, $CHCl_3$).

methyl (2S,3S)-3-(4-aminophenyl)-2-methyl-3-(10-oxo-9,10-dihydroanthracen-9-yl)propanoate (5c)

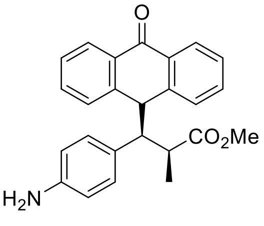

In a 2-neck round-bottom flask (RBF) methyl (R)-2-((4-aminophenyl)(10-oxo-9,10-dihydroanthracen-9-yl)methyl)acrylate (4c) (1.0 equiv, 20 mg, 0.054 mmol) was added together with Pd/C (0.15 equiv w/w, 3 mg). EtOAc (1 ml, 0.054 mol/L) was added. The RBF was sealed and flushed twice with Argon. Then the reaction mixture was flushed once with H_2. The rubber balloon was refilled with H_2 and connected to the RBF. The reaction was monitored by TLC

until the reaction completion (overnight). The mixture was filtered through Celite, washed with EtOAc and concentrated in vacuo. The mixture was purified by Column chromatography (5:1 Hexane:EtOAc) to obtain 18 mg of desired product. The product yield is 60 %. ^{1}H NMR (400 MHz, $CDCl_3$) σ 8.02 – 7.96 (m, 1H), 7.91 (d, *J* = 7.1 Hz, 1H), 7.64 – 7.53 (m, 3H), 7.47 – 7.41 (m, 2H), 7.37 (dd, *J* = 10.8, 4.0 Hz, 1H), 6.09 (d, *J* = 8.4 Hz, 2H), 5.55 (d, *J* = 8.4 Hz, 2H), 4.71 (d, *J* = 2.9 Hz, 1H), 3.27 (s, 3H), 3.15 (dd, *J* = 11.6, 3.0 Hz, 1H), 3.09 – 3.00 (m, 1H), 1.76 (d, *J* = 6.6 Hz, 3H). ^{13}C NMR (101 MHz, $CDCl_3$) σ 195.4 (q), 175.5 (q), 144.7 (q), 144.6 (q), 143.8 (q), 140.6 (q), 134.9 (q), 132.4, 131.6, 130.2, 129.3 (q), 127.9, 127.8, 127.4, 126.9, 126.8, 126.2, 113.9, 59.2, 51.4, 44.2, 42.2, 17.3. HR-MS (m/z) for $C_{25}H_{23}NO_3$ calculated $[M+Na]^+$ 408.1570, measured $[M+Na]^+$ 408.1572. IR (cm^{-1}): 3420 and 3365 (NH_2, stretch), 1733 (C=O, stretch), 1665 (C=O, stretch), 1315 (C-O ester, stretch), 1195, 1168, 932, 756, 695. $[\alpha]_D^{21}$ = -6.4 (c = 0.006 g/ml, $CHCl_3$).

methyl (2S,3S)-2-methyl-3-(10-oxo-9,10-dihydroanthracen-9-yl)-3-(p-tolyl)propanoate (5d)

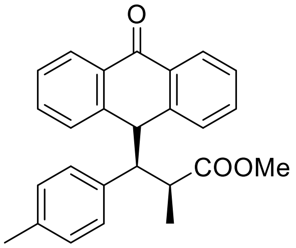

In a 2-neck round-bottom flask (RBF) methyl (R)-2-((10-oxo-9,10-dihydroanthracen-9-yl)(p-tolyl)methyl)acrylate (4d) (1.0 equiv, 25 mg, 0.065 mmol) was added together with Pd/C (0.15 equiv w/w, 3 mg). EtOAc (1 ml, 0.065 mol/L) was added. The RBF was sealed and flushed twice with Argon. Then the reaction mixture was flushed once with H_2. The rubber balloon was refilled with H_2 and connected to the RBF. The reaction was monitored by TLC until the reaction completion (2 days). The mixture was filtered through Celite, washed with EtOAc and concentrated in vacuo. The mixture was purified by Column chromatography (5:1 Hexane:EtOAc) to obtain 18 mg of desired product. The product yield is 72 %. ^{1}H NMR (400 MHz, $CDCl_3$) σ 7.99 (dd, *J* = 8.3, 1.2 Hz, 1H), 7.90 (d, *J* = 7.7 Hz, 1H), 7.66 – 7.55 (m, 3H), 7.44 (dd, *J* = 11.4, 4.3 Hz, 2H), 7.41 – 7.34 (m, 1H), 6.55 (d, *J* = 7.9 Hz, 2H), 5.68 (d, *J* = 8.1 Hz, 2H), 4.73 (d, *J* = 3.1 Hz, 1H), 3.29 – 3.20 (m, 4H), 3.12 (dd, *J* = 11.6, 6.6 Hz, 1H), 2.12 (s, 3H), 1.78 (d, *J* = 6.6 Hz, 3H). ^{13}C NMR (101 MHz, $CDCl_3$) σ 183.2 (q), 175.5 (q), 144.5 (q), 140.5 (q), 136.6 (q), 134.7 (q), 133.7 (q), 132.5, 132.2, 131.7, 129.0, 128.0, 127.9, 127.7, 127.5, 127.0, 126.9, 126.3, 59.5, 51.4, 43.9, 42.2, 21.0, 17.3. LC-MS (m/z) for $C_{26}H_{24}O_3$ $[M+H]^+$ 385.32. HR-MS (m/z) for $C_{26}H_{24}O_3$ calculated $[M+Na]^+$ 407.1618, measured $[M+Na]^+$ 407.1615. IR (cm^{-1}): 3062 (=C-H aromatic, stretch), 2949 (=C-H

aromatic, stretch), 1668 (C=O, stretch), 1164, 1100, 1055, 931. $[\alpha]_D^{21}$ = -11.5 (c = 0.005 g/ml, $CHCl_3$).

<u>(2S,3S)-2-methyl-3-(10-oxo-9,10-dihydroanthracen-9-yl)-3-phenylpropanenitrile</u> (5m)

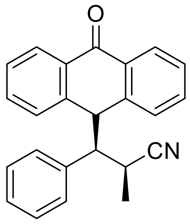

In a 2-neck round-bottom flask (RBF) (R)-2-((10-oxo-9,10-dihydroanthracen-9-yl)(phenyl)methyl)acrylonitrile (4m) (1.0 equiv, 25 mg, 0.074 mmol) was added together with Pd/C (0.15 equiv w/w, 3 mg). EtOAc (1 ml, 0.074 mol/L) was added. The RBF was sealed and flushed twice with Argon. Then the reaction mixture was flushed once with H_2. The rubber balloon was refilled with H_2 and connected to the RBF. The reaction was monitored by TLC until the reaction completion (2 days). The mixture was filtered through Celite, washed with EtOAc and concentrated in vacuo. The mixture was purified by Column chromatography (5:1 Hexane:EtOAc) to obtain 16 mg of desired product. The product yield is 68 %. Dr is 8:1. ^{1}H NMR (400 MHz, $CDCl_3$) σ 8.09 (ddd, *J* = 27.9, 7.7, 0.9 Hz, 2H), 7.82 (d, *J* = 7.6 Hz, 1H), 7.65 (td, *J* = 7.5, 1.3 Hz, 1H), 7.50 (t, *J* = 7.1 Hz, 1H), 7.32 (dd, *J* = 11.0, 4.1 Hz, 1H), 7.24 – 7.16 (m, 2H), 7.12 (t, *J* = 7.6 Hz, 2H), 6.78 (dd, *J* = 20.3, 7.4 Hz, 3H), 4.57 (d, *J* = 8.4 Hz, 1H), 2.80 (p, *J* = 6.9 Hz, 1H), 2.57 (dd, *J* = 8.3, 6.6 Hz, 1H), 1.20 (d, *J* = 7.0 Hz, 3H). ^{13}C NMR (101 MHz, $CDCl_3$) σ185.0 (q), 142.9 (q), 142.0 (q), 136.4 (q), 133.2 (q), 132.5, 131.4, 129.0, 128.5, 128.4, 128.3, 128.1, 128.0, 127.8, 127.7 (q), 127.5, 127.4, 122.0 (q), 61.3, 47.5, 28.8, 17.3. HR-MS (m/z) for $C_{24}H_{19}NO$ calculated $[M+H]^+$ 338.1539, measured $[M+H]^+$ 338.1538. IR (cm^{-1}): 3066 and 3031 (C=CH aromatic, stretch), 2244 (C≡N, stretch), 1668 (C=O, stretch), 1170, 966, 709. $[\alpha]_D^{21}$ = -8.0 (c = 0.005 g/ml, $CHCl_3$).

<u>10-((1S,2S)-1-(4-bromophenyl)-2-methyl-3-oxobutyl)anthracen-9(10H)-one</u> (5r)

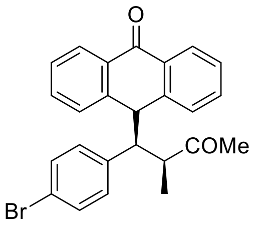

In a 2-neck round-bottom flask (RBF) (R)-10-(1-(4-bromophenyl)-2-methylene-3-oxobutyl)anthracen-9(10H)-one (4r) (1.0 equiv, 25 mg, 0.058 mmol) was added together with Pd/C (0.15 equiv w/w, 3 mg). EtOAc (1 ml, 0.058 mol/L) was added. The RBF was sealed and flushed twice with Argon. Then the reaction mixture was flushed once with H_2. The rubber balloon was refilled with H_2 and connected to the RBF. The reaction was monitored by TLC until the reaction completion (2 days). The mixture was filtered through Celite, washed with EtOAc and concentrated in

vacuo. The mixture was purified by Column chromatography (5:1 Hexane:EtOAc) to obtain 22 mg of desired product. The product yield is 88 %. ^{1}H NMR (400 MHz, $CDCl_3$) σ 8.03 (d, *J* = 7.6 Hz, 1H), 7.93 (d, *J* = 7.8 Hz, 1H), 7.64 (t, *J* = 7.4 Hz, 2H), 7.57 (d, *J* = 7.5 Hz, 1H), 7.49 (dd, *J* = 12.3, 7.4 Hz, 2H), 7.40 (t, *J* = 7.5 Hz, 1H), 6.89 (d, *J* = 8.3 Hz, 2H), 5.65 (d, *J* = 8.2 Hz, 2H), 4.74 (s, 1H), 3.36 – 3.10 (m, 2H), 1.81 (s, 3H), 1.71 (d, *J* = 6.1 Hz, 3H). ^{13}C NMR (101 MHz, $CDCl_3$) σ 210.5 (q), 183.0 (q), 144.0 (q), 140.1 (q), 134.9 (q), 134.7 (q), 133.5 (q), 132.8, 131.9, 130.7 (q), 130.4, 127.9, 127.8, 127.7, 127.3, 127.2, 126.5, 121.4, 58.7, 48.5, 43.7, 29.3, 16.8. HR-MS (m/z) for $C_{25}H_{21}BrO_2$ calculated $[M+H]^+$ 433.0798, measured $[M+H]^+$ 433.0806. IR (cm^{-1}): 3066 (=C-H aromatic, stretch), 3027 (=C-H aromatic, stretch), 1667 (C=O, stretch), 1156, 1073, 1010, 931. $[\alpha]_D^{21}$ = -9.2 (c = 0.007 g/ml, $CHCl_3$).

<u>10-((1S,2S)-2-methyl-3-oxo-1-phenylpentyl)anthracen-9(10H)-one</u> (5u)

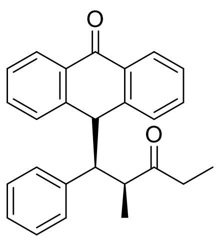

In a 2-neck round-bottom flask (RBF) methyl (R)-10-(2-methylene-3-oxo-1-phenylpentyl)anthracen-9(10H)-one (5u) (1.0 equiv, 25 mg, 0.068 mmol) was added together with Pd/C (0.15 equiv w/w, 3 mg). EtOAc (1 ml, 0.068 mol/L) was added. The RBF was sealed and flushed twice with Argon. Then the reaction mixture was flushed once with H_2. The rubber balloon was refilled with H_2 and connected to the RBF. The reaction was monitored by TLC until the reaction completion (3 days). The mixture was filtered through Celite, washed with EtOAc and concentrated in vacuo. The mixture was purified by Column chromatography (10:1 Hexane:EtOAc) to obtain 13 mg of desired product. The product yield is 52 %. ^{1}H NMR (400 MHz, $CDCl_3$) σ 7.99 (dd, *J* = 7.8, 1.2 Hz, 1H), 7.87 (dd, *J* = 7.8, 1.0 Hz, 1H), 7.67 – 7.56 (m, 3H), 7.53 (d, *J* = 7.6 Hz, 1H), 7.46 (td, *J* = 7.8, 1.1 Hz, 1H), 7.40 – 7.34 (m, 1H), 7.02 – 6.94 (m, 1H), 6.74 (t, *J* = 7.8 Hz, 2H), 5.74 (d, *J* = 7.2 Hz, 2H), 4.76 (d, *J* = 2.7 Hz, 1H), 3.39 – 3.19 (m, 2H), 2.33 – 2.15 (m, 1H), 1.99 (ddd, *J* = 18.0, 15.2, 7.9 Hz, 1H), 1.70 (d, *J* = 6.3 Hz, 3H), 0.66 (t, *J* = 7.2 Hz, 3H). ^{13}C NMR (101 MHz, $CDCl_3$) σ 190.7, 170.1, 140.6, 137.8, 137.6, 136.7, 136.2, 135.5, 134.9, 133.7, 132.6, 131.7, 129.3, 128.0, 127.8, 127.5, 127.2, 127.1, 127.0, 126.9, 126.2, 59.3, 47.7, 44.0, 35.6, 17.1. LC-MS (m/z) for $C_{26}H_{24}O_2$ $[M+H]^+$ 369.29. HR-MS (m/z) for $C_{26}H_{24}O_2$ calculated $[M+Na]^+$ 391.1669, measured $[M+Na]^+$ 391.1676. IR (cm^{-1}): 3067 (=C-H aromatic, stretch), 2927 (=C-H aromatic, stretch), 1669 (C=O, stretch), 1313, 1073, 1010, 710. $[\alpha]_D^{21}$ = -3 (c = 0.004 g/ml, $CHCl_3$).

Kinetic resolution

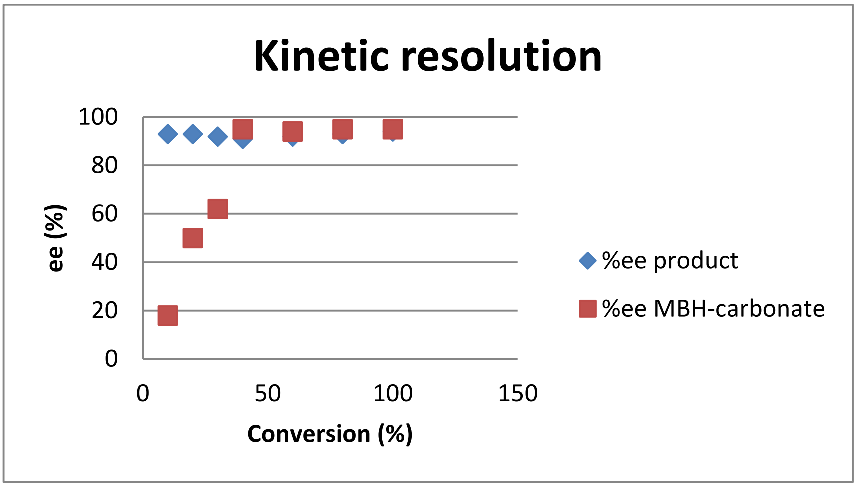

These are the results from HPLC analysis which I made the graphic:

**conversion**	**10**	**20**	**30**	**40**	**60**	**80**	**100**
**%ee product**	**93**	**93**	**92**	**91**	**92**	**93**	**94**
**%ee MBH-carbonate**	**18**	**50**	**62**	**95**	**94**	**95**	**95**

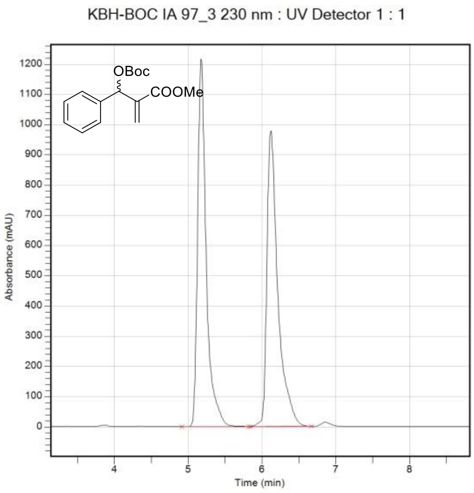

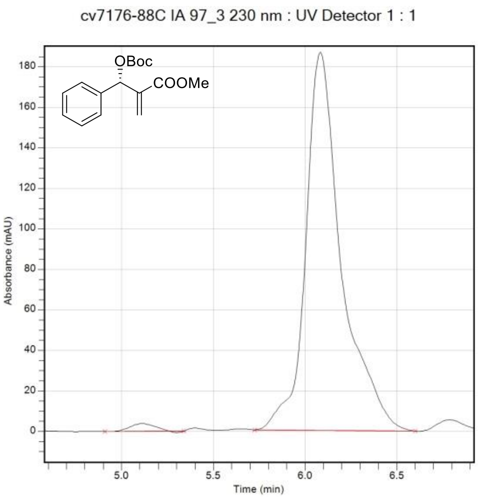

Time	Area	Area %
5.177	9,906,048.4	49.52
6.121	10,099,707.2	50.48
**Total**	20,005,755.5	100.00

Time	Area	Area %
5.115	14,039.4	2.56
6.100	534,132.1	97.44
**Total**	548,171.5	100.00

NMR spectra

methyl (R)-2-((10-oxo-9,10-dihydroanthracen-9-yl)(phenyl)methyl)acrylate (4a)

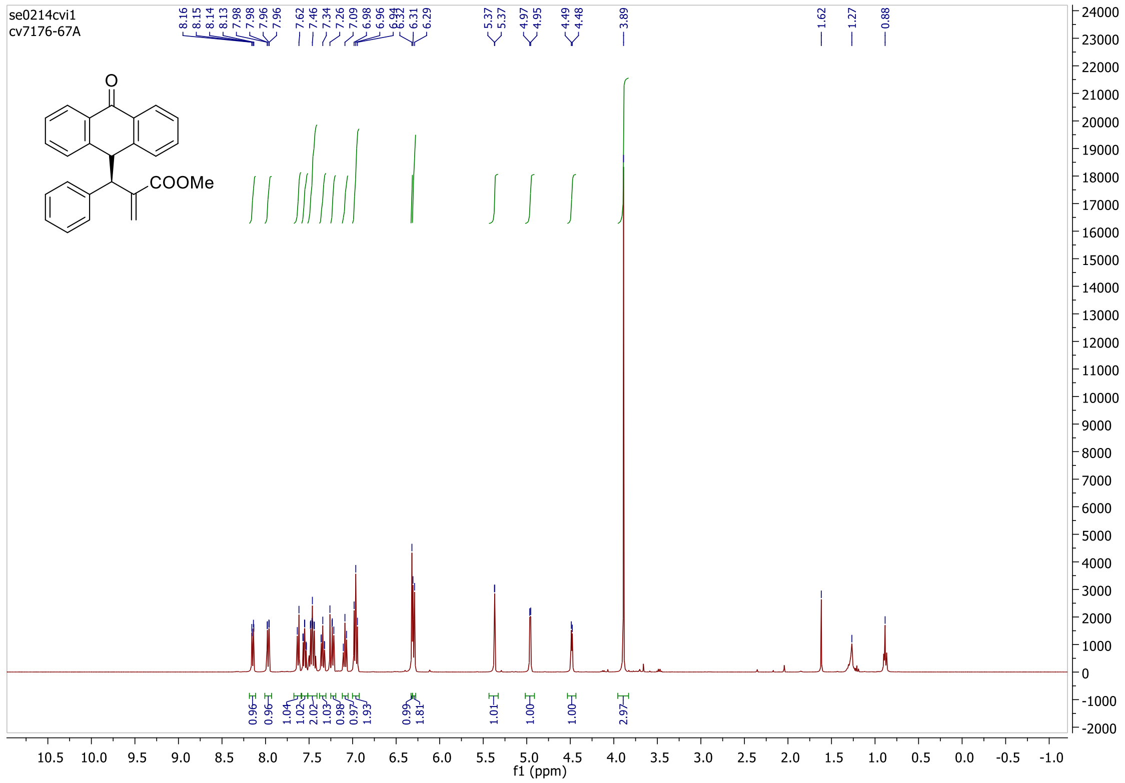

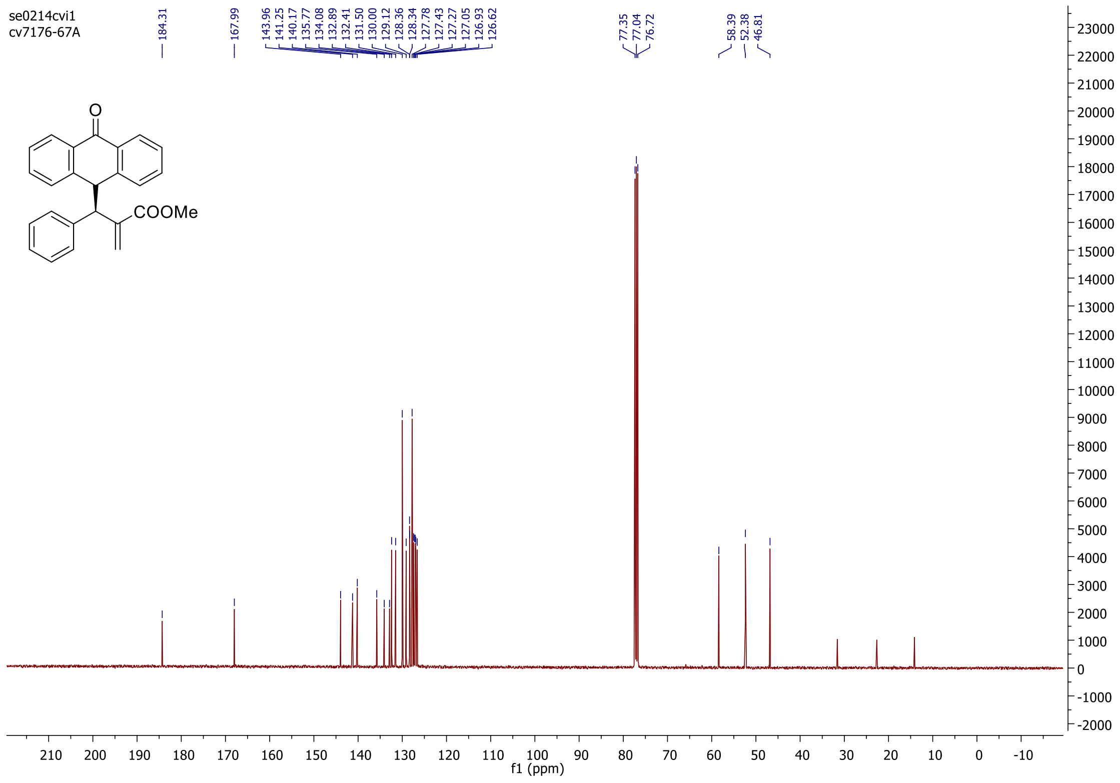

methyl (R)-2-((4-fluorophenyl)(10-oxo-9,10-dihydroanthracen-9-yl)methyl)acrylate (4b)

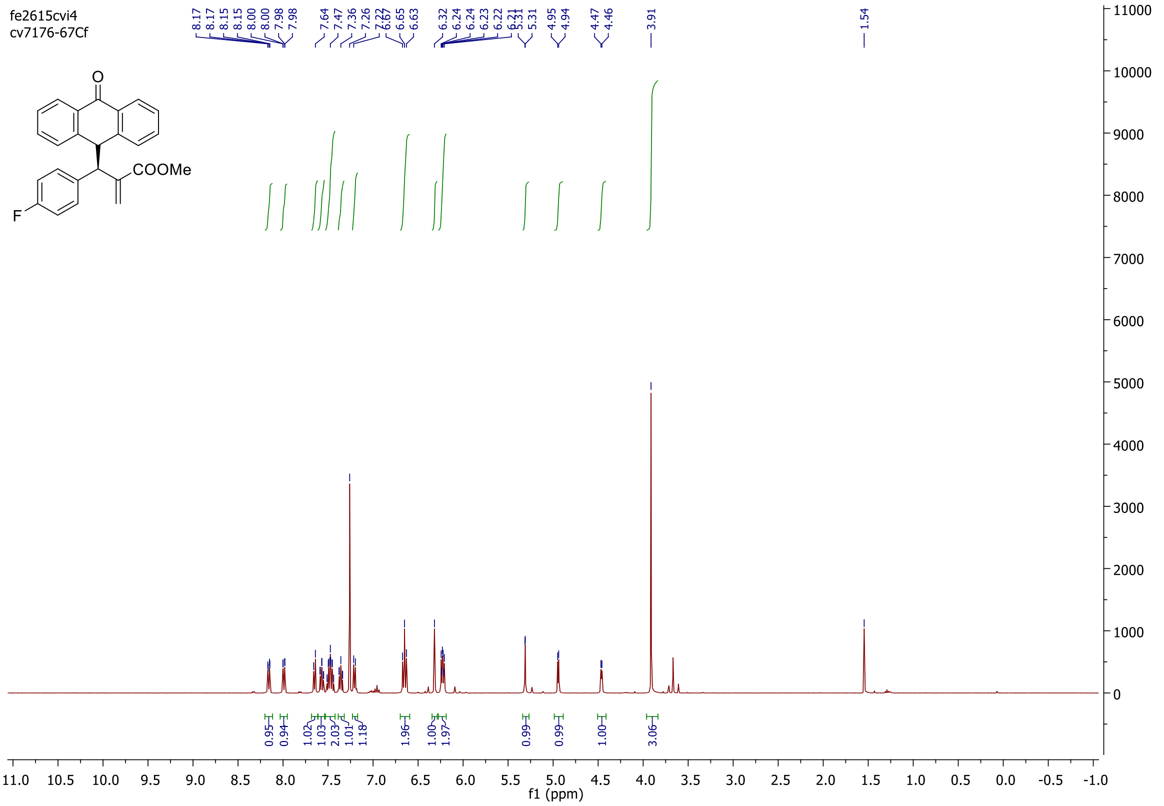

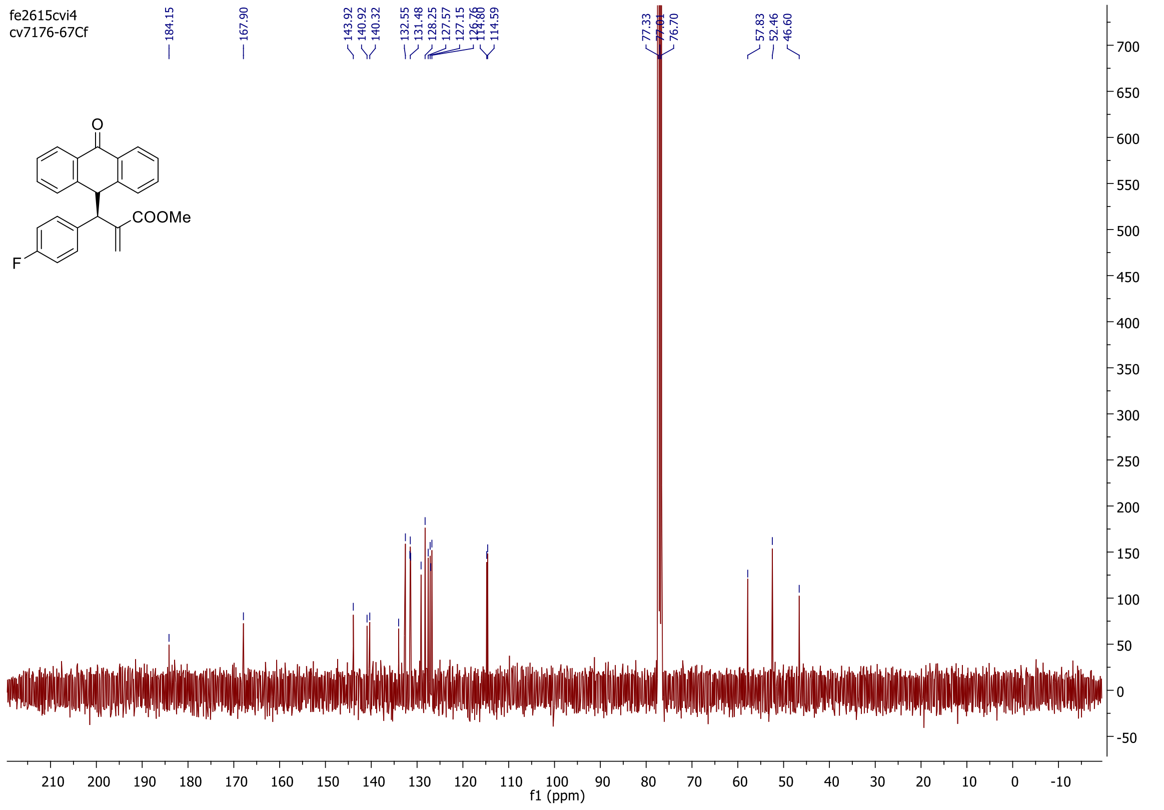

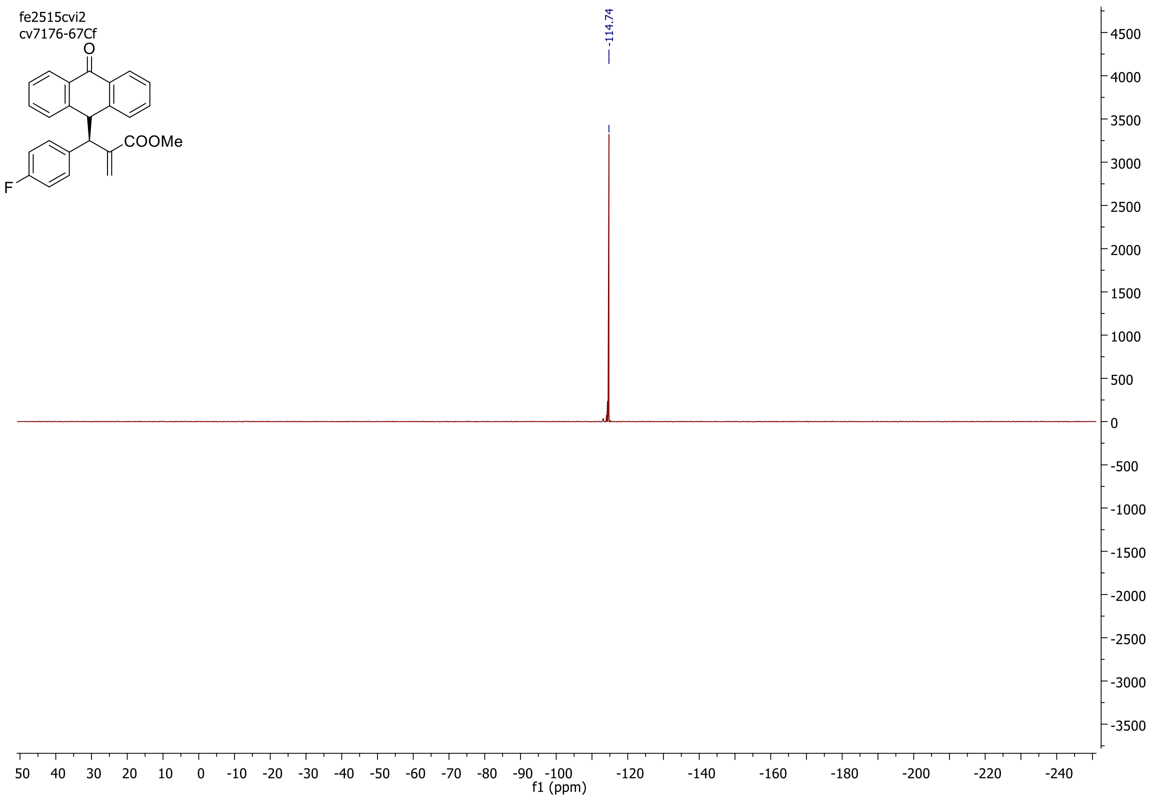

<u>**methyl (R)-2-((4-nitrophenyl)(10-oxo-9,10-dihydroanthracen-9-yl)methyl)acrylate**</u> **(4c)**

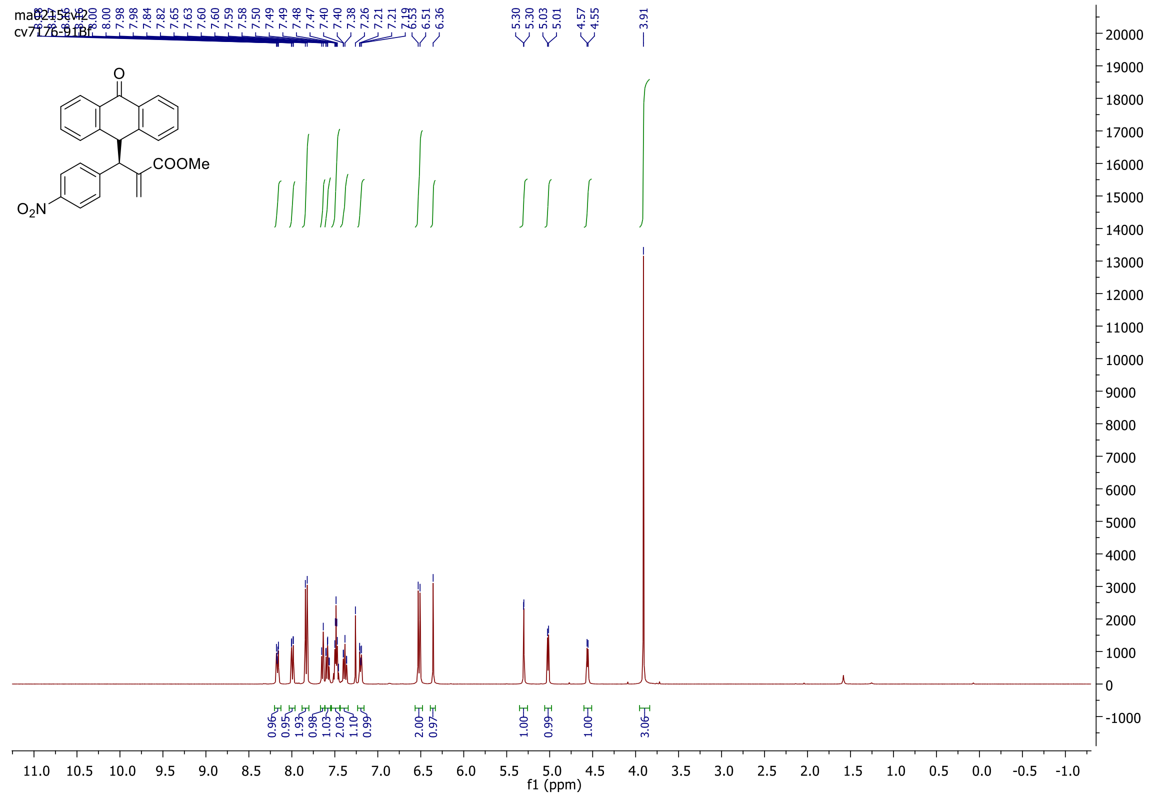

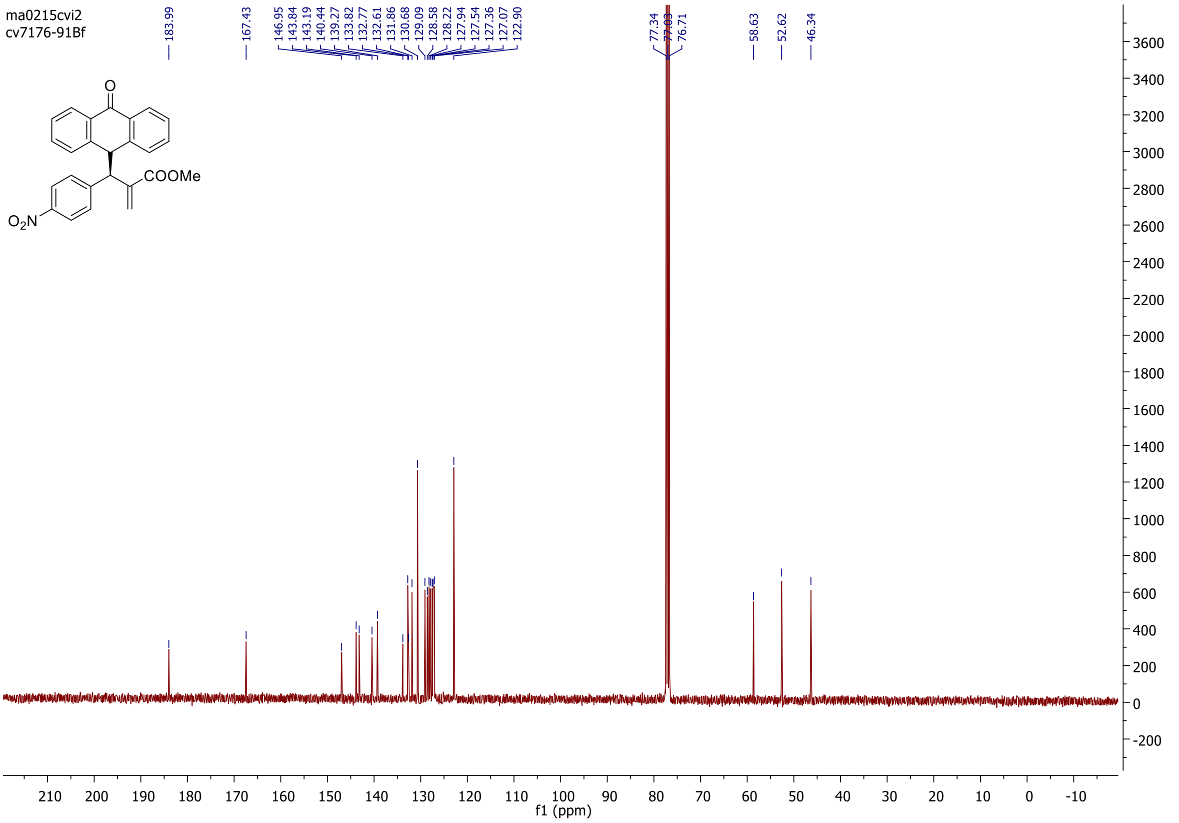

<u>methyl (R)-2-((10-oxo-9,10-dihydroanthracen-9-yl)(p-tolyl)methyl)acrylate</u> (4d)

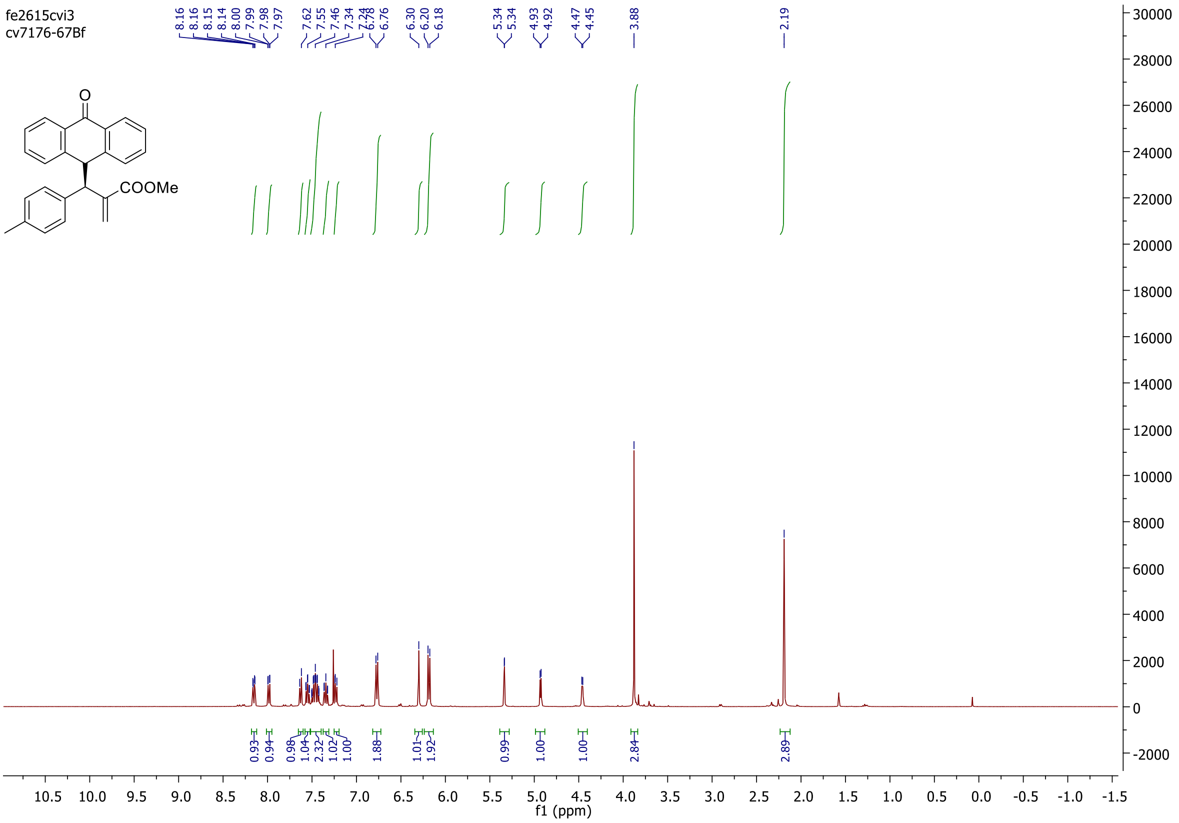

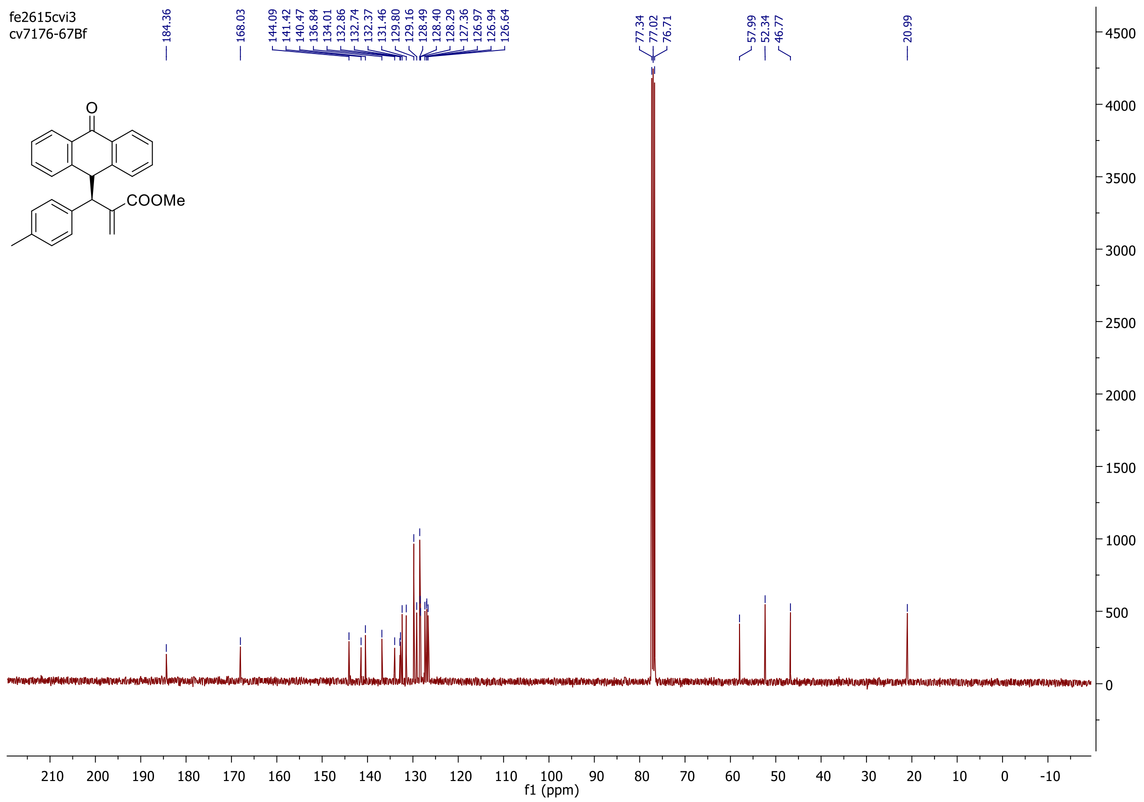

methyl (R)-2-((4-chlorophenyl)(10-oxo-9,10-dihydroanthracen-9-yl)methyl)acrylate (4e)

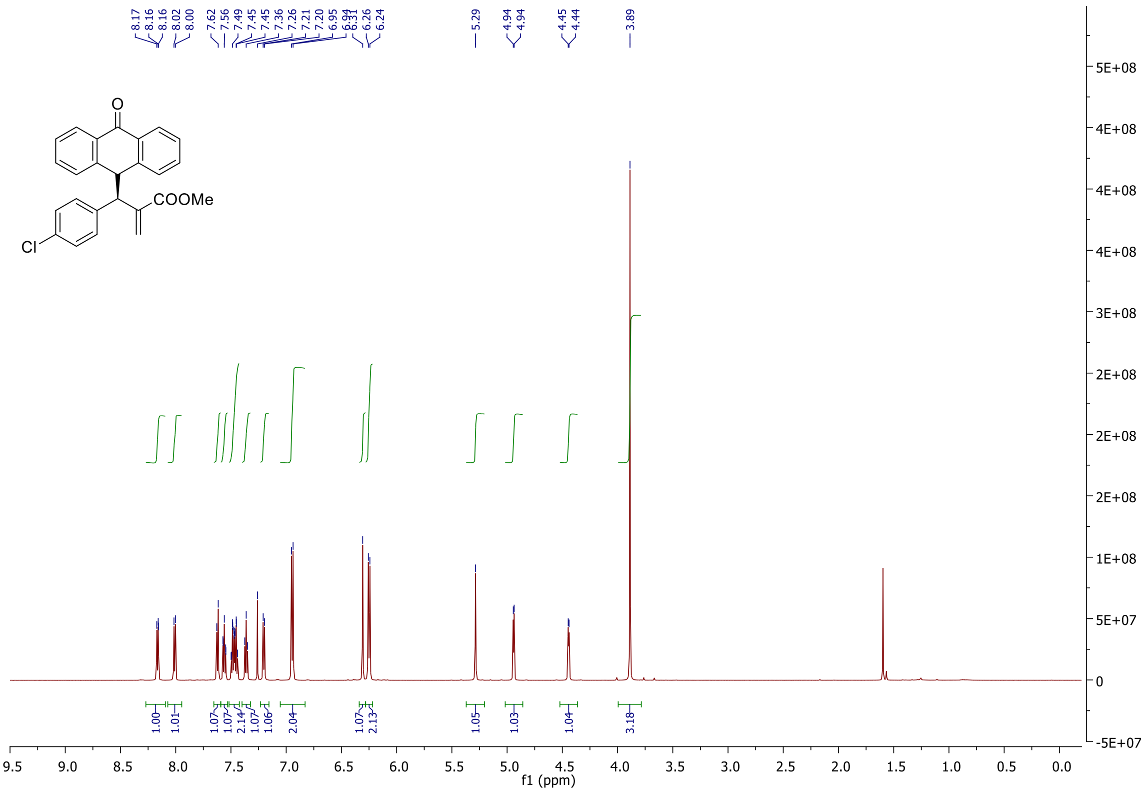

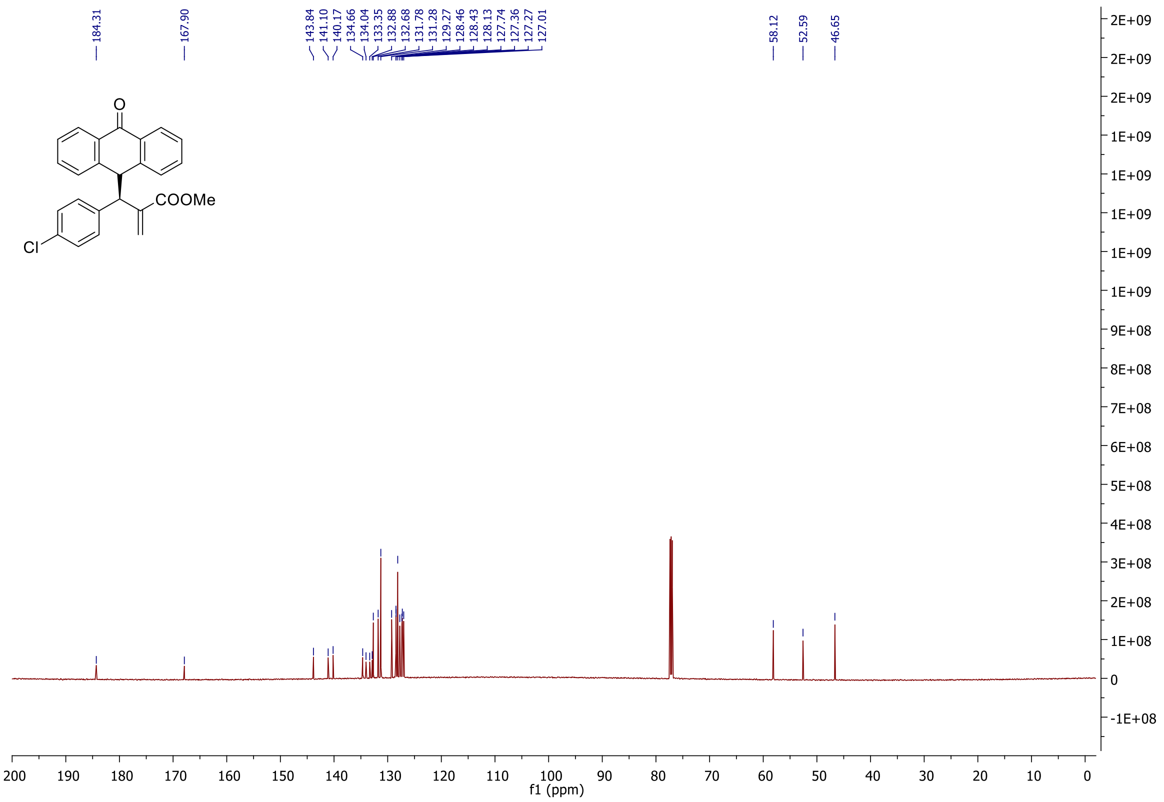

methyl (R)-2-((3-chlorophenyl)(10-oxo-9,10-dihydroanthracen-9-yl)methyl)acrylate (4f)

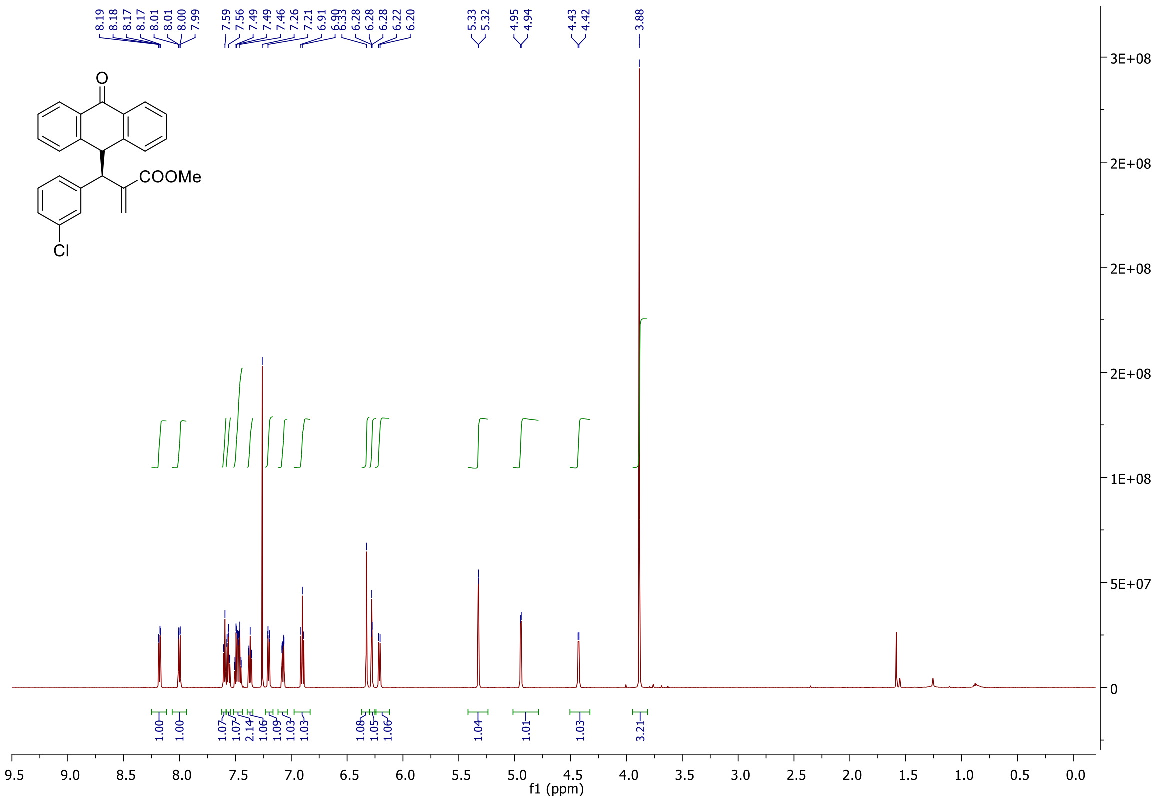

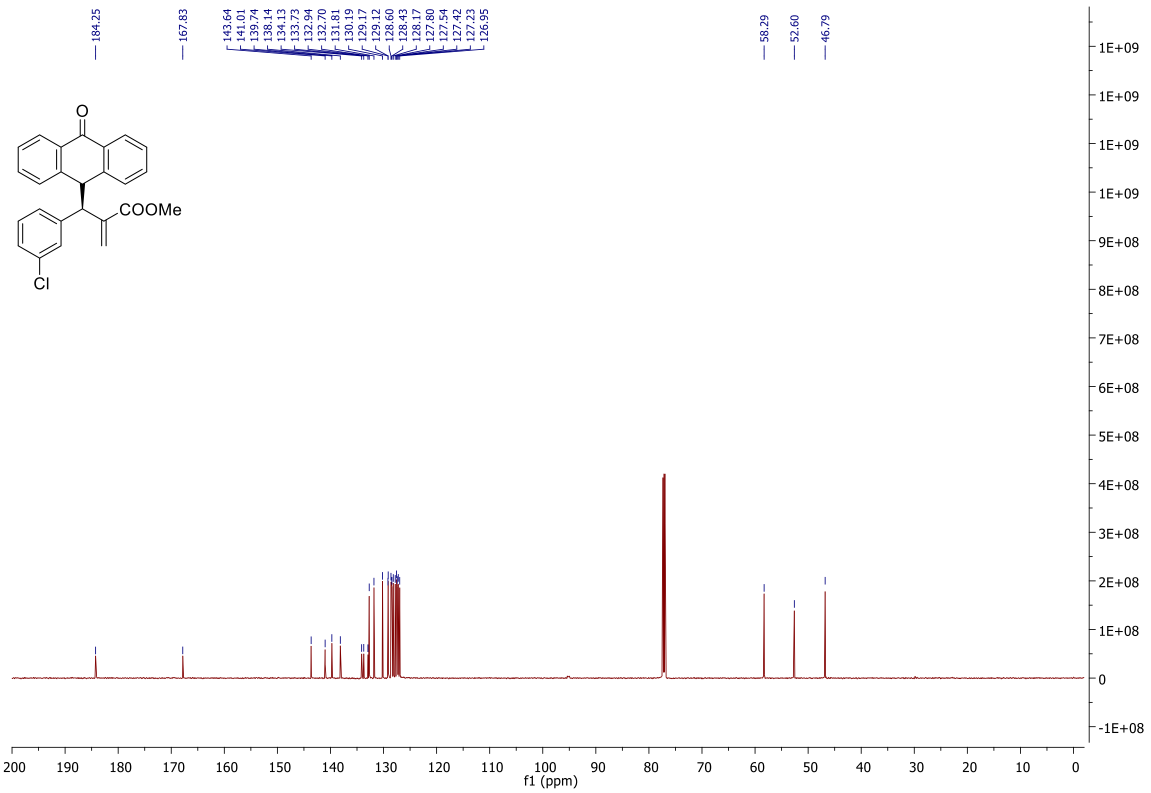

methyl (S)-2-((2-chlorophenyl)(10-oxo-9,10-dihydroanthracen-9-yl)methyl)acrylate (4g)

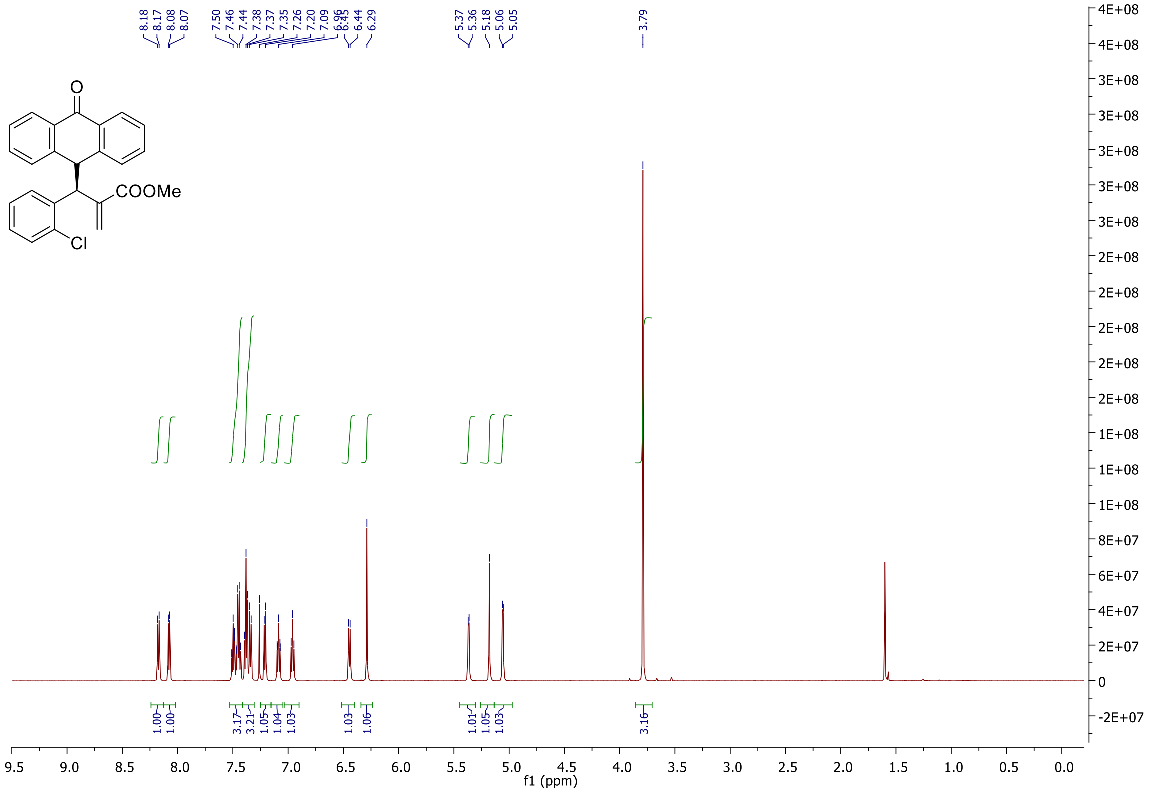

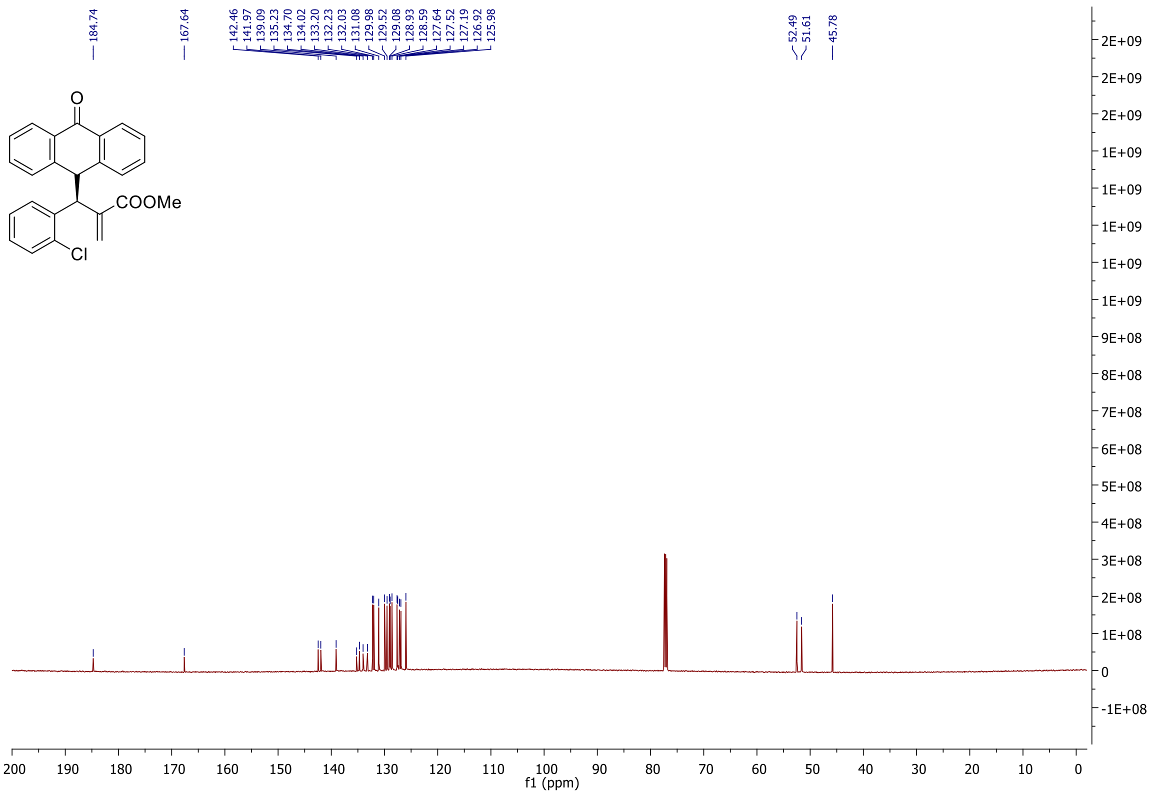

<u>methyl (R)-2-((4-bromophenyl)(10-oxo-9,10-dihydroanthracen-9-yl)methyl)acrylate</u> (4h)

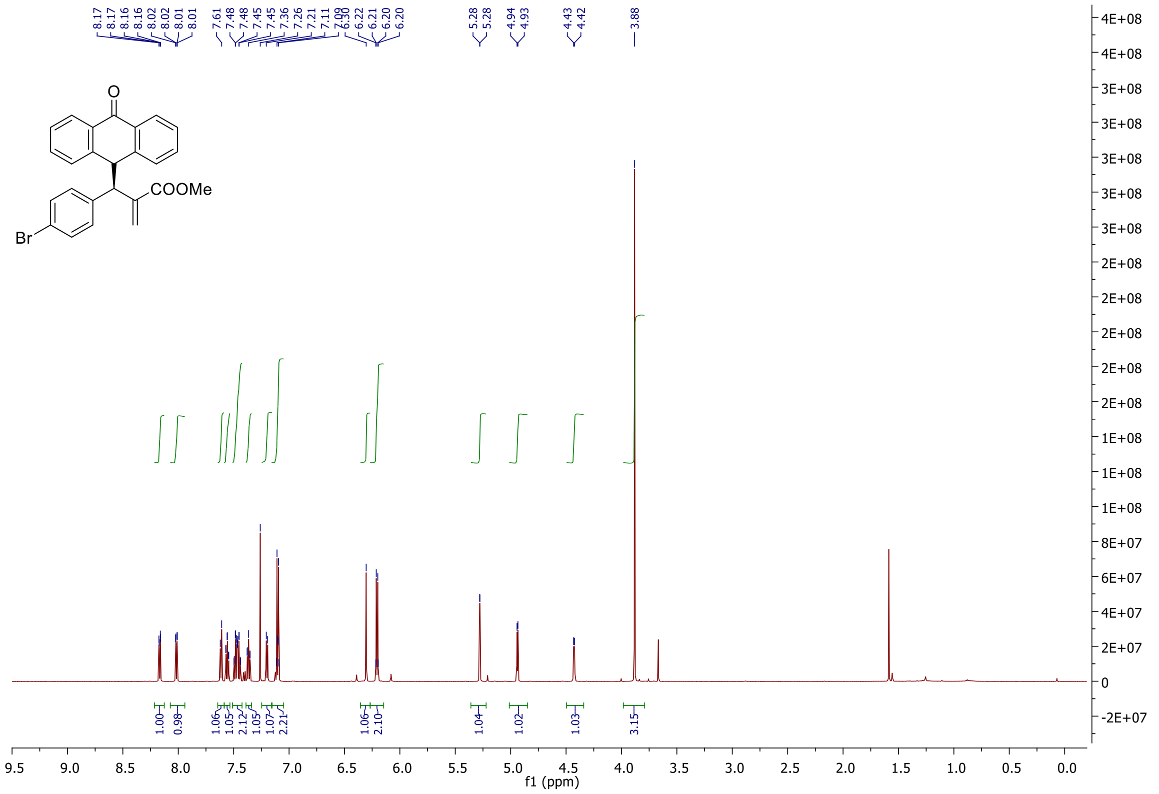

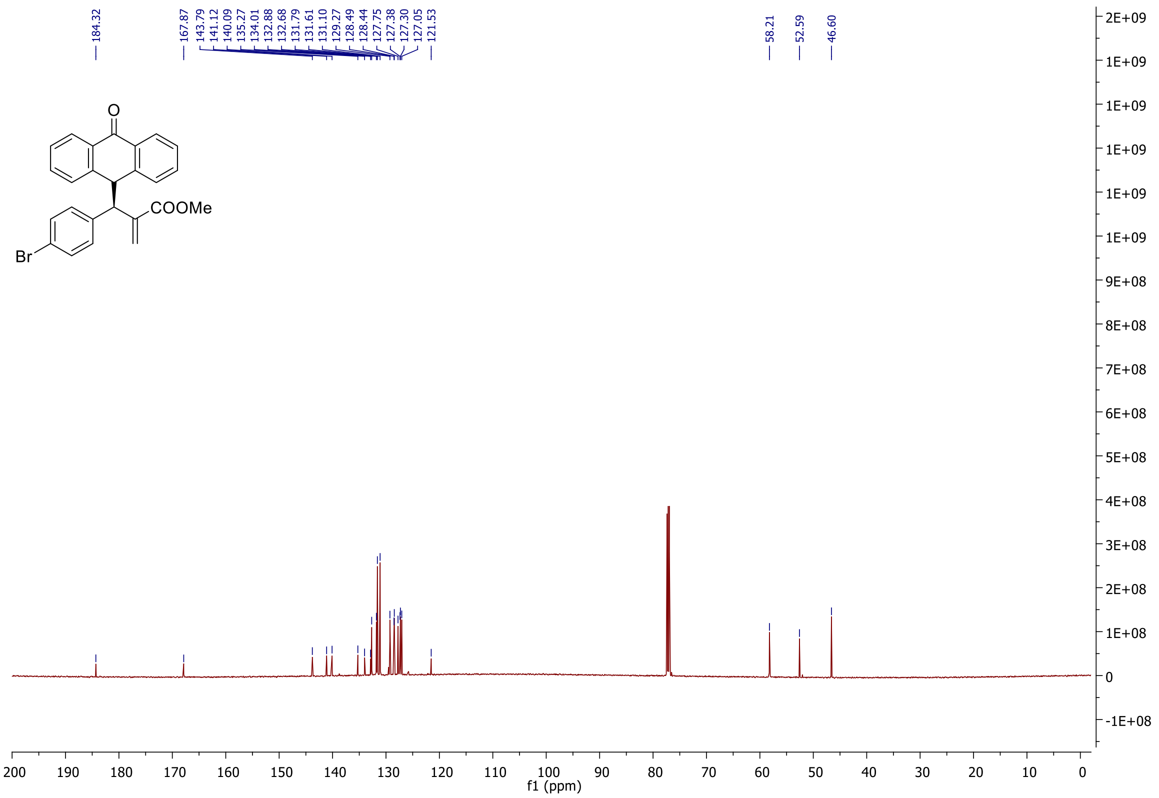

methyl (R)-2-((3-bromophenyl)(10-oxo-9,10-dihydroanthracen-9-yl)methyl)acrylate (4i)

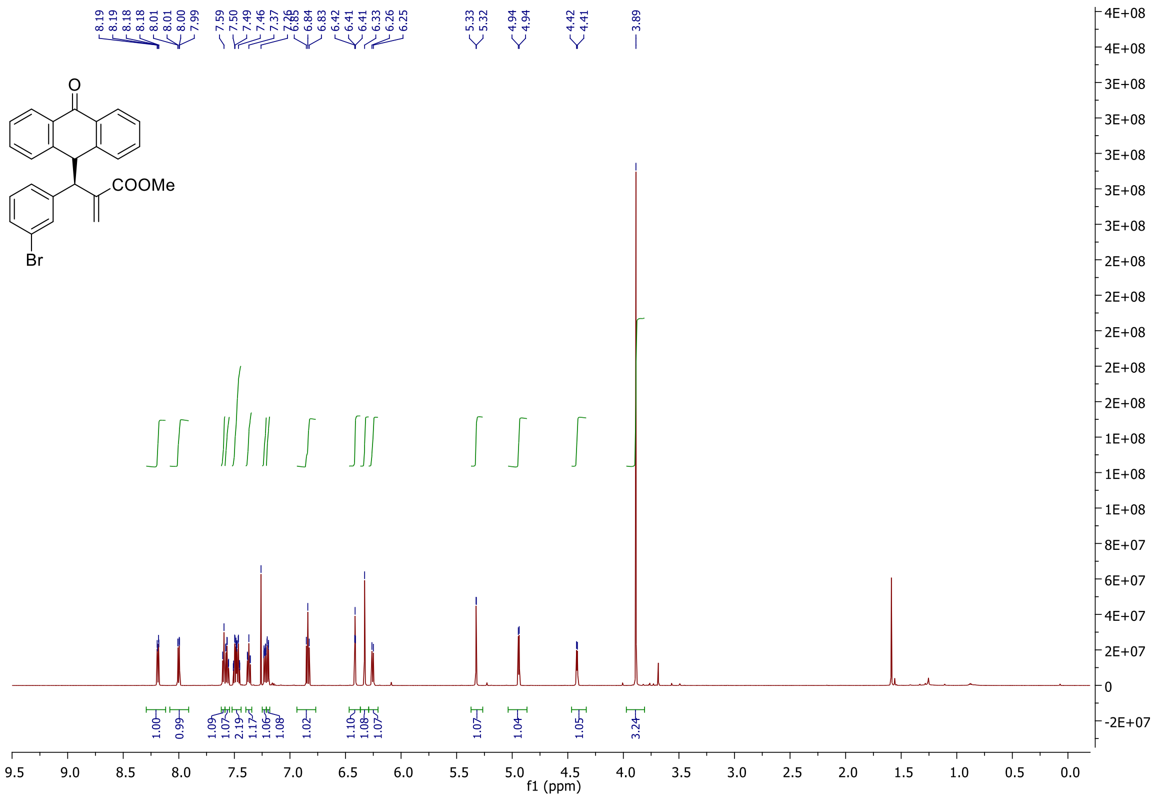

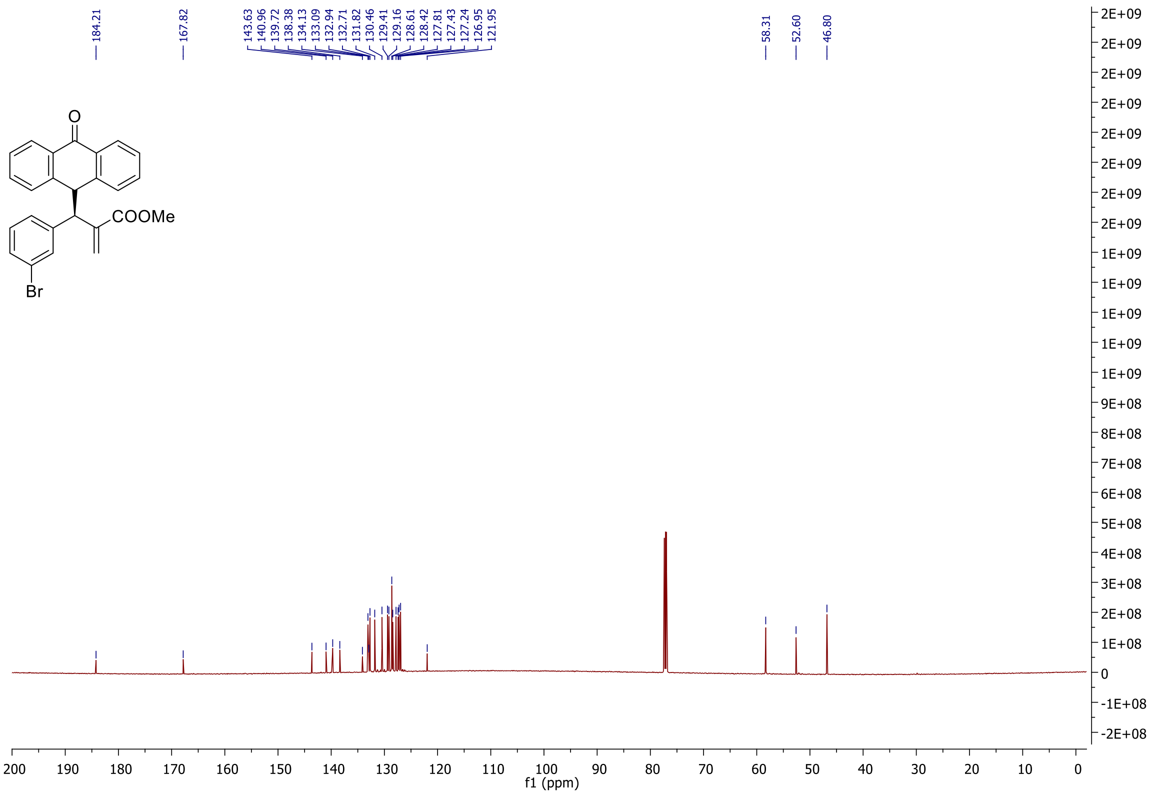

methyl (S)-2-((2-bromophenyl)(10-oxo-9,10-dihydroanthracen-9-yl)methyl)acrylate (4j)

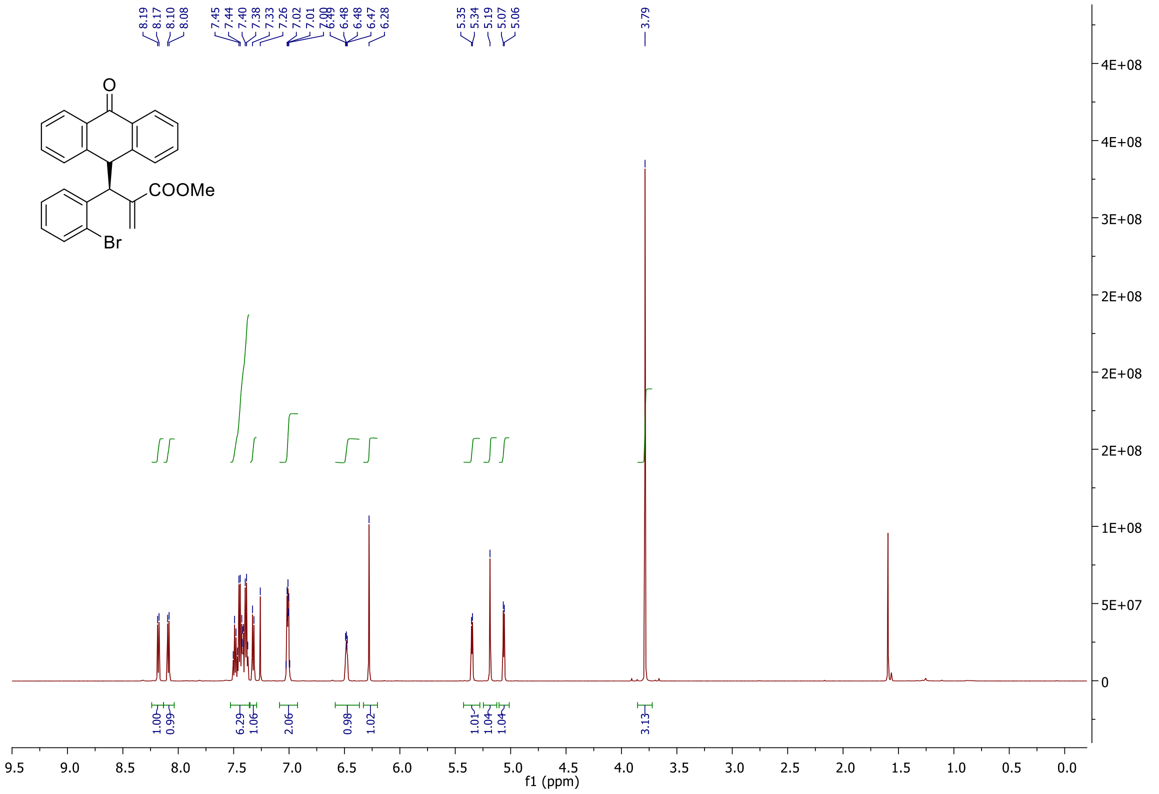

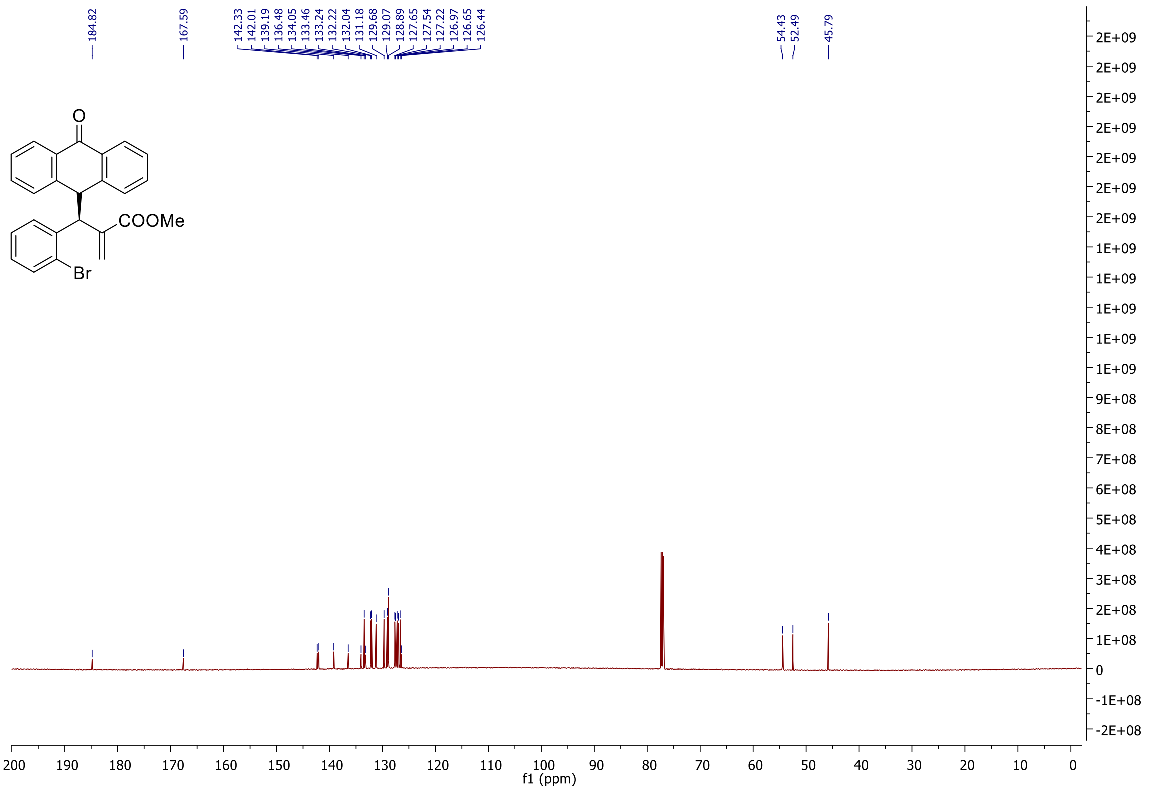

methyl (S)-2-((10-oxo-9,10-dihydroanthracen-9-yl)(thiophen-2-yl)methyl)acrylate (4k)

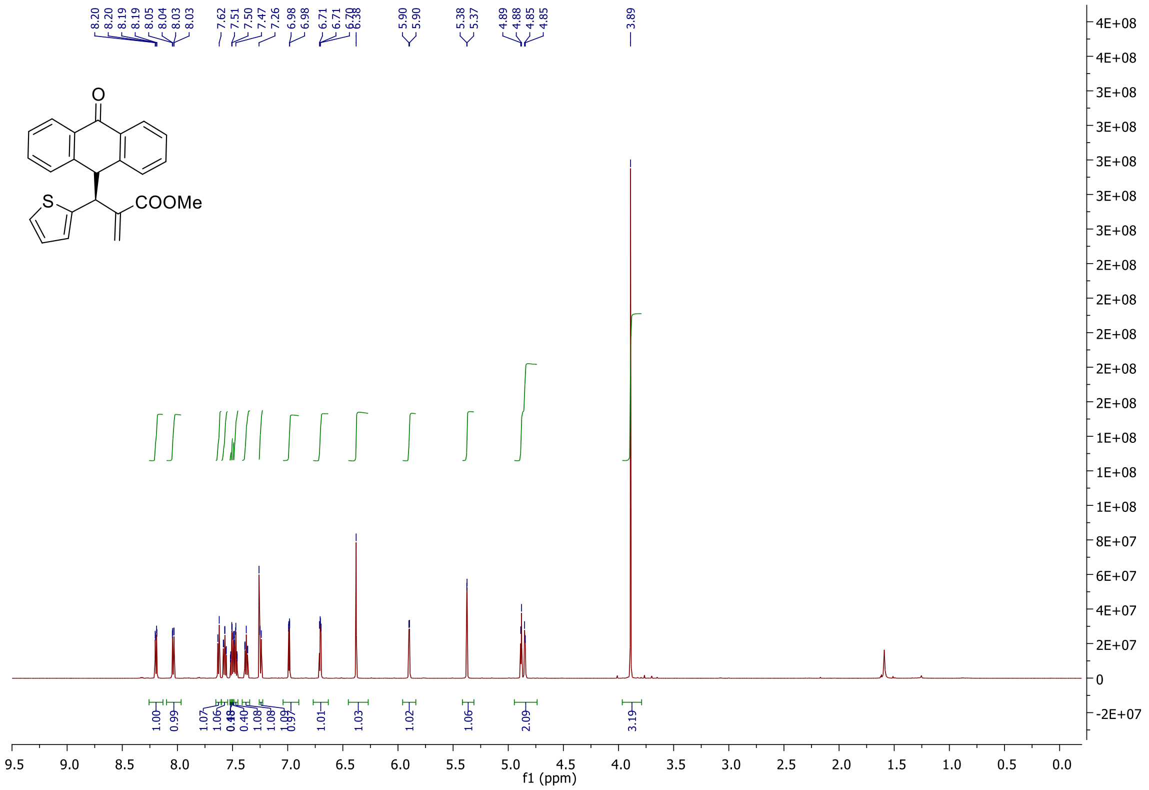

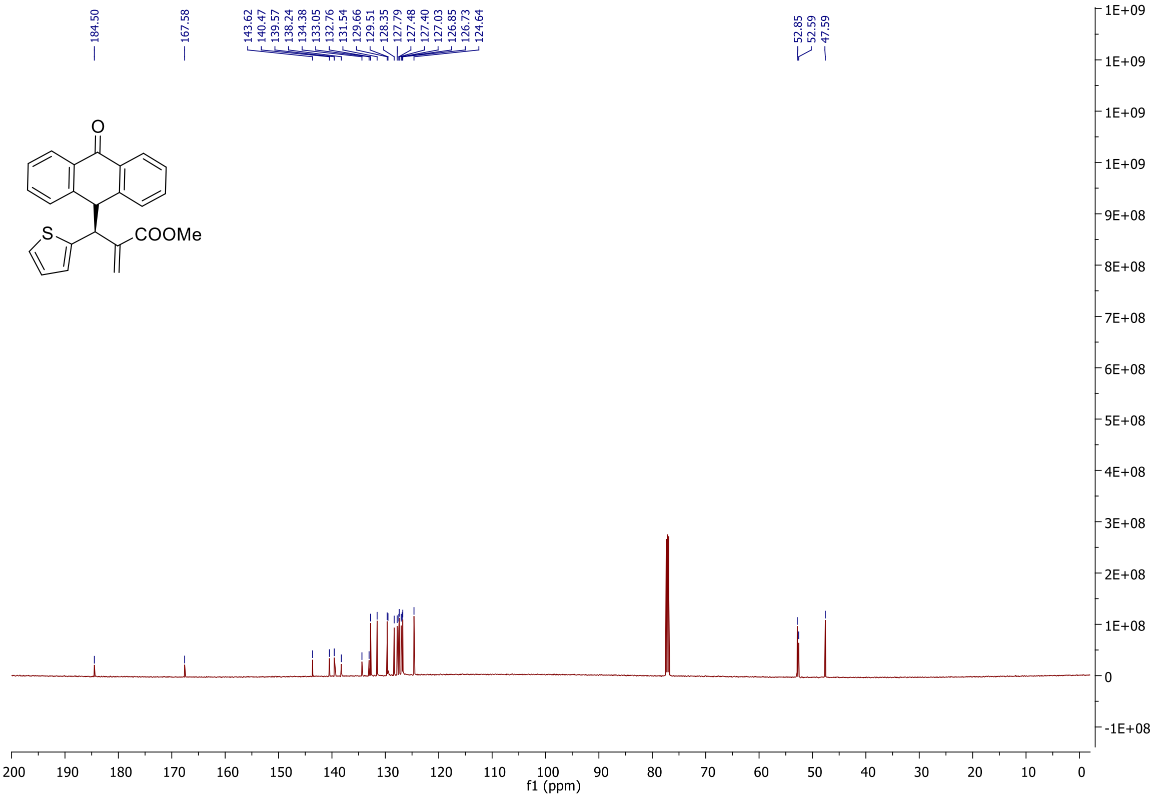

methyl (S)-2-methylene-3-(10-oxo-9,10-dihydroanthracen-9-yl)-5-phenylpentanoate (4l)

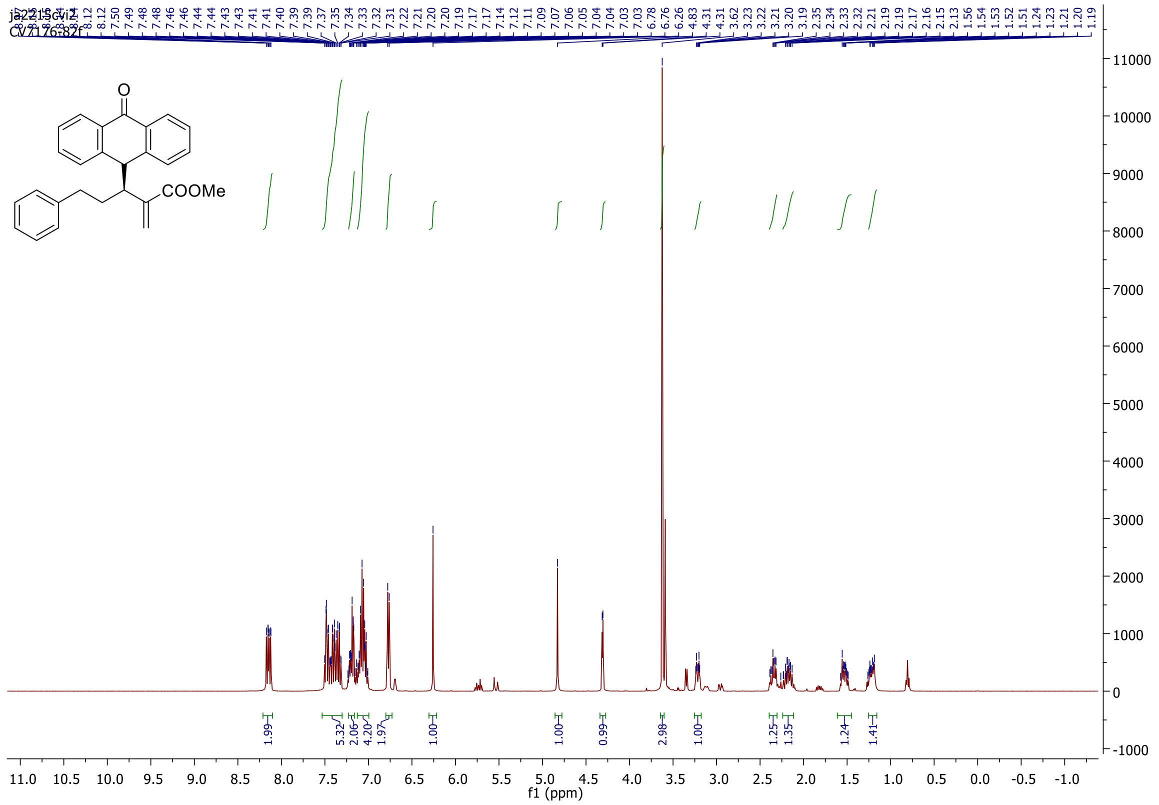

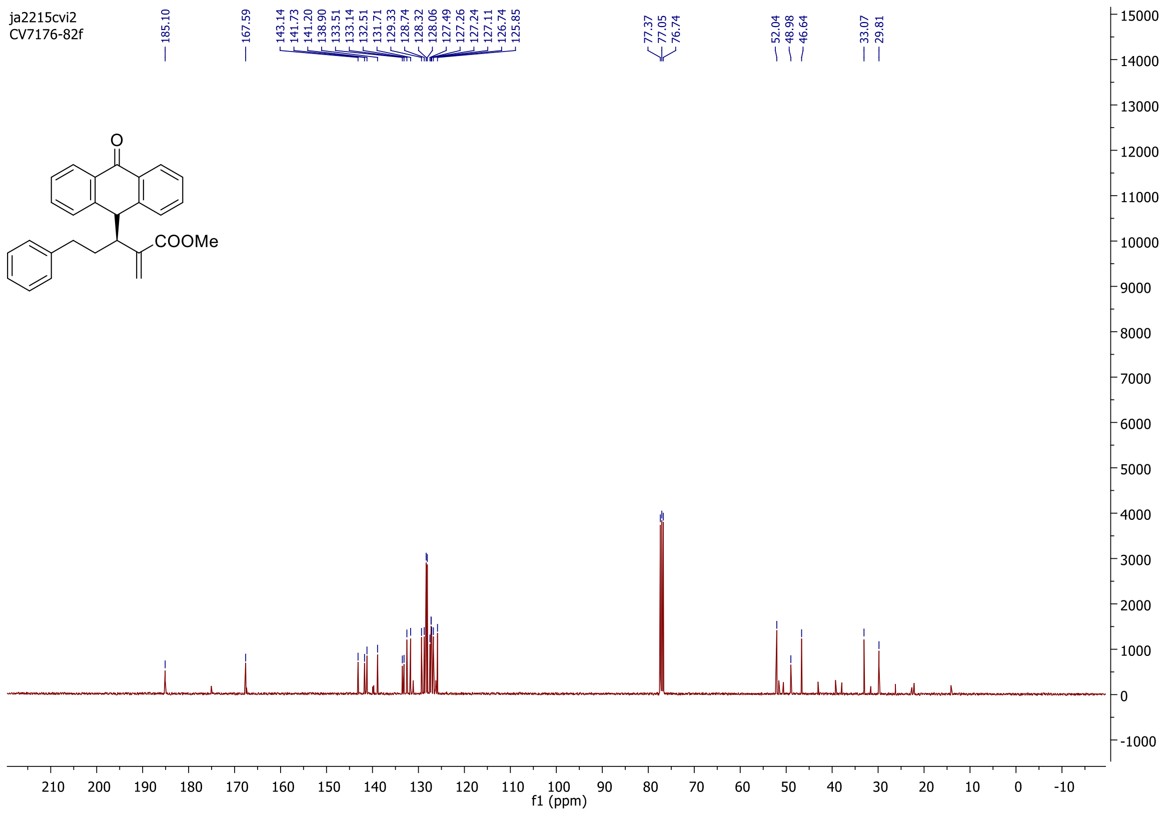

(R)-2-((10-oxo-9,10-dihydroanthracen-9-yl)(phenyl)methyl)acrylonitrile (4m)

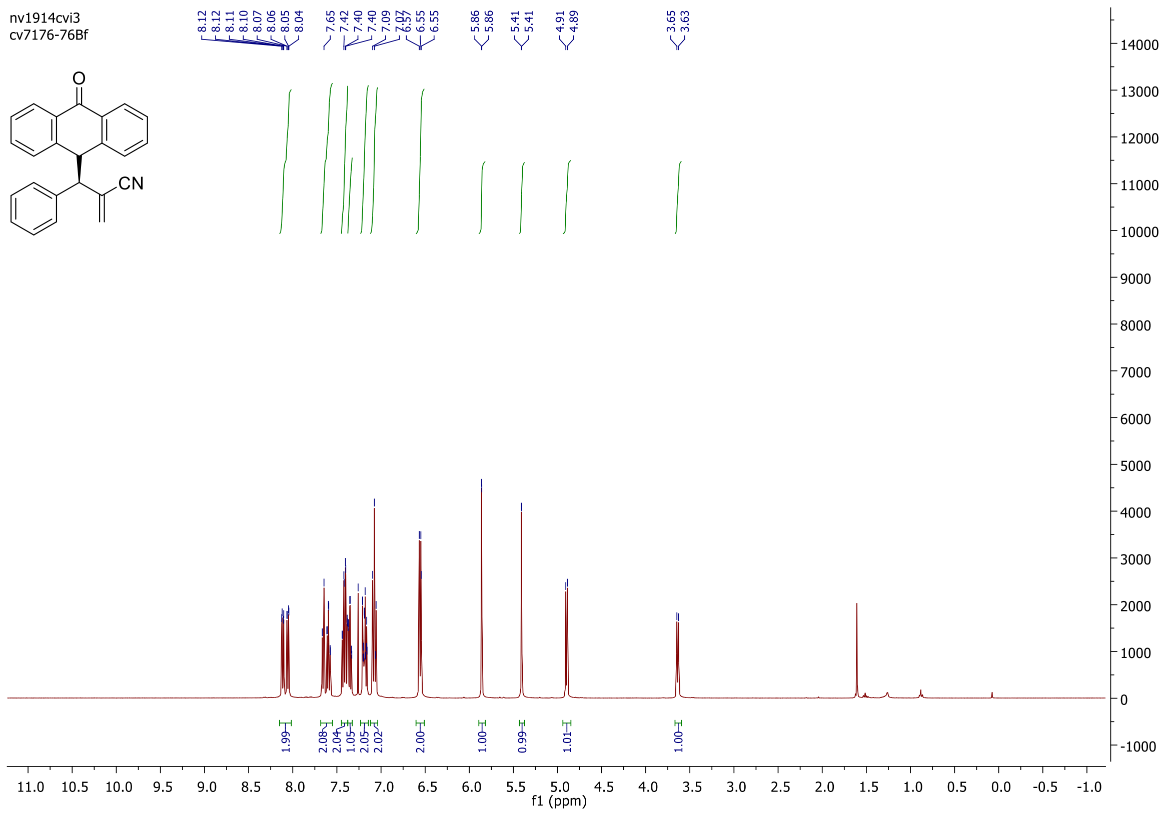

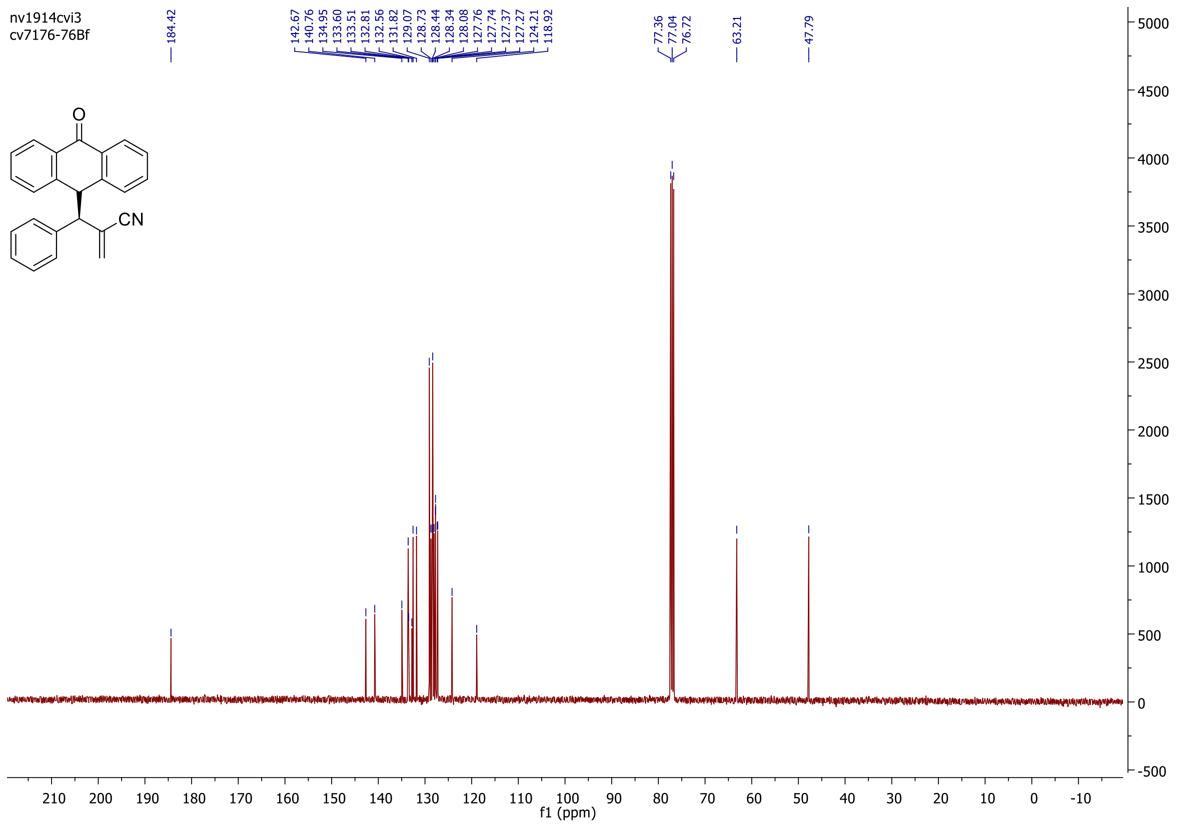

(R)-2-((10-oxo-9,10-dihydroanthracen-9-yl)(p-tolyl)methyl)acrylonitrile (4n)

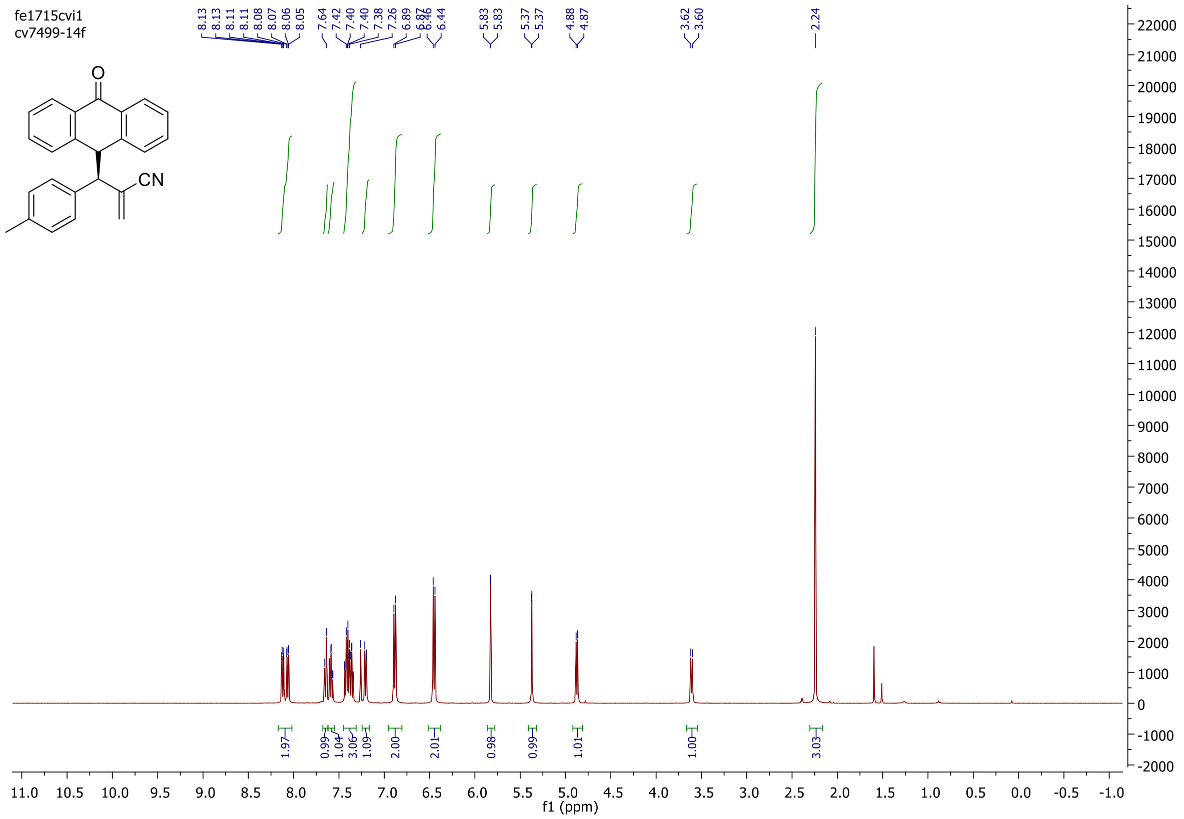

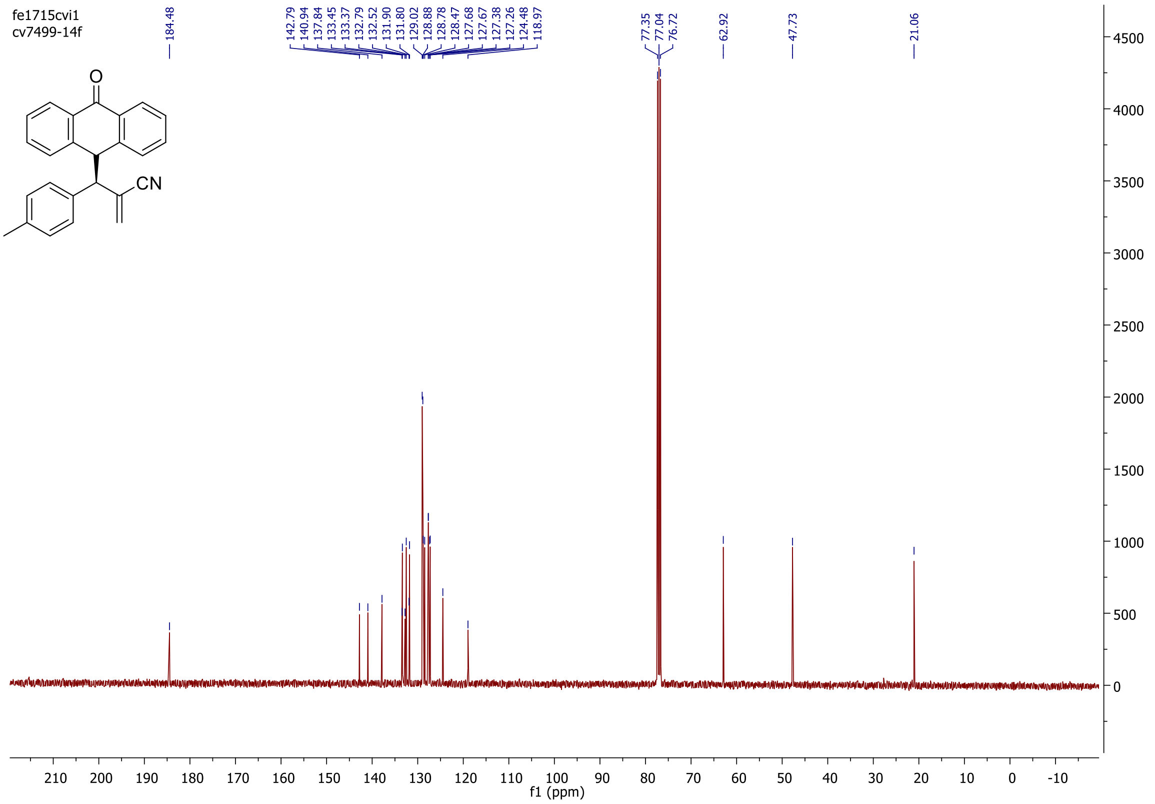

(R)-10-(2-methylene-3-oxo-1-phenylbutyl)anthracen-9(10H)-one (4o)

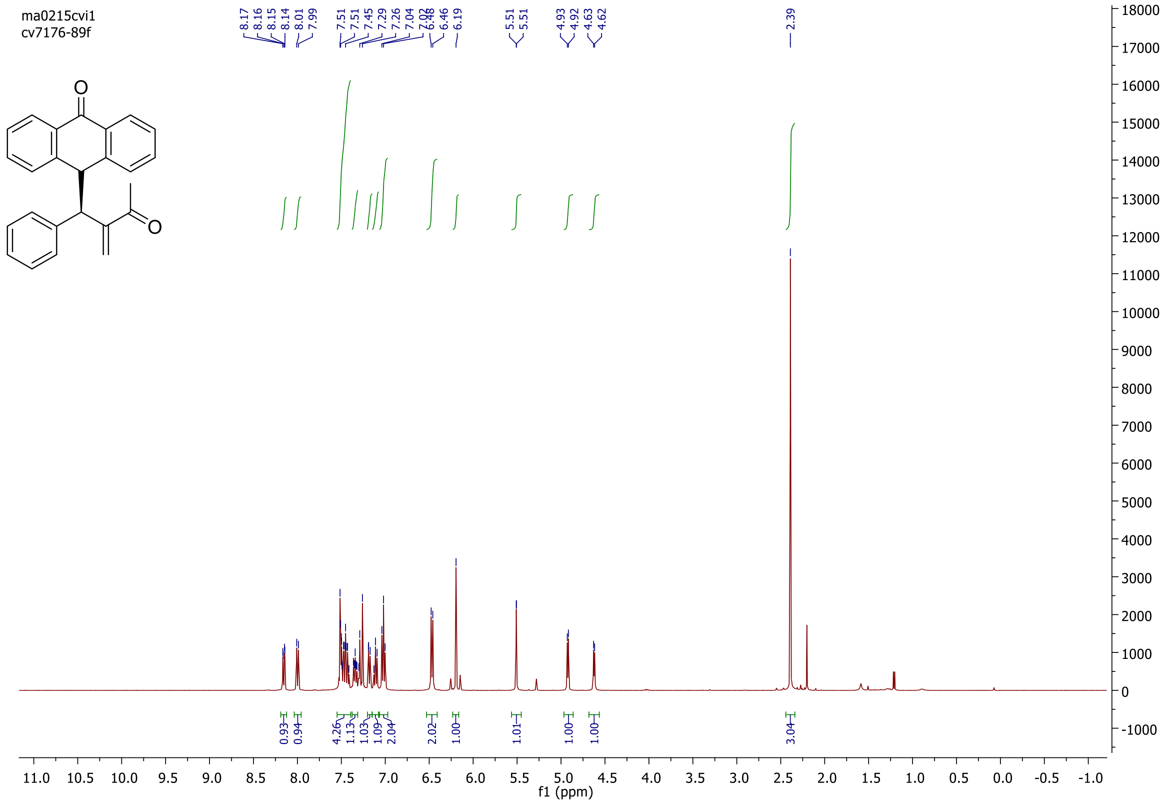

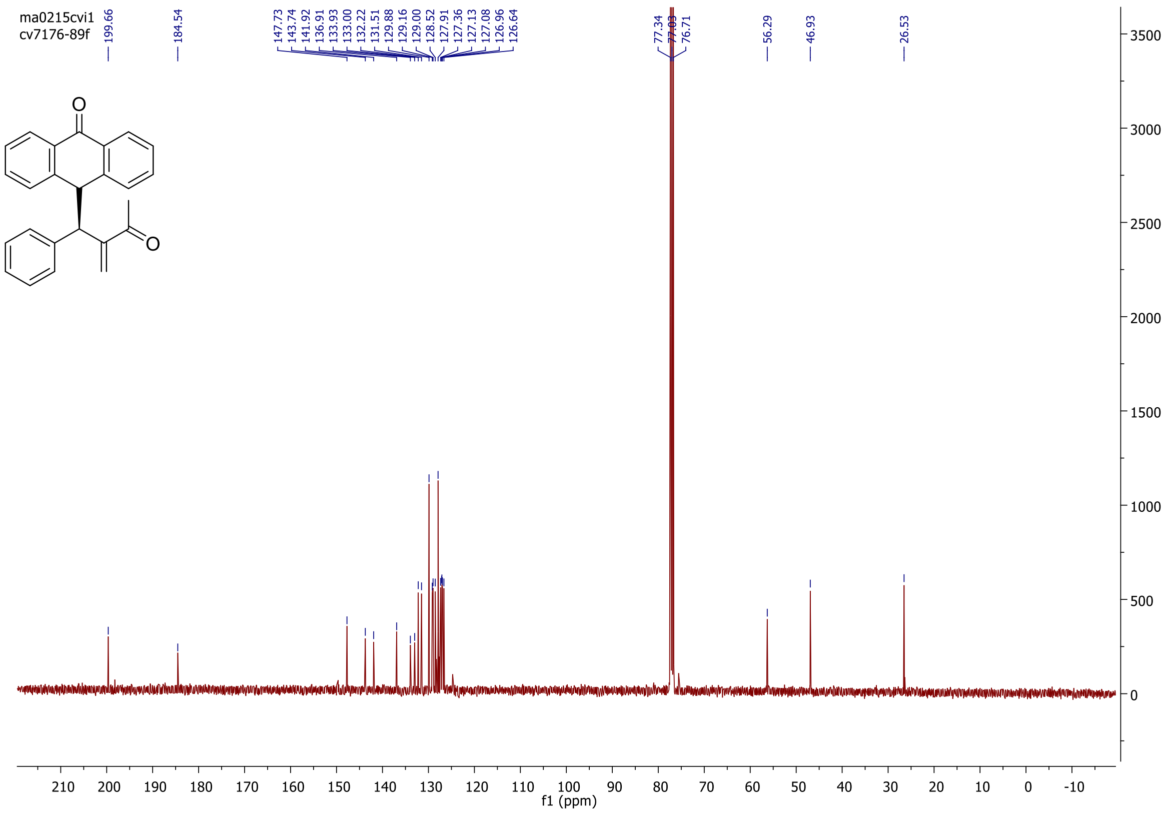

(R)-10-(2-methylene-3-oxo-1-(p-tolyl)butyl)anthracen-9(10H)-one (4p)

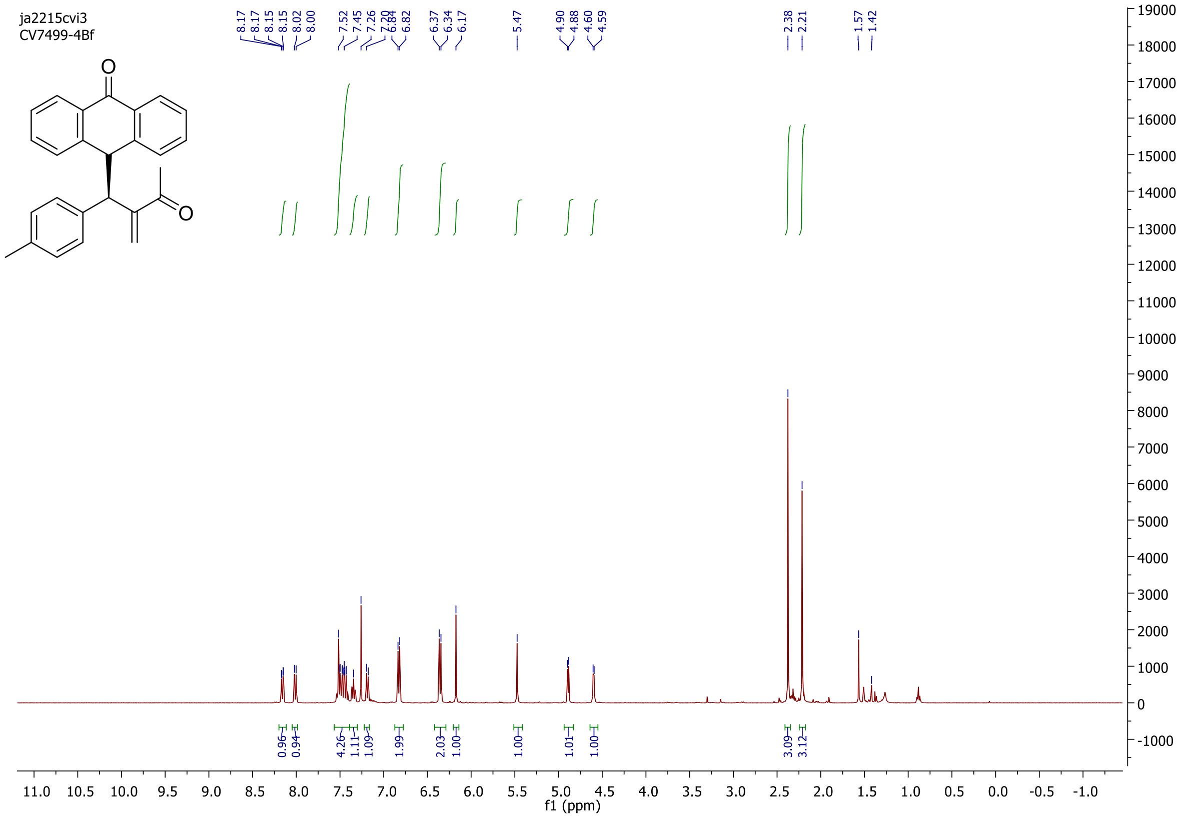

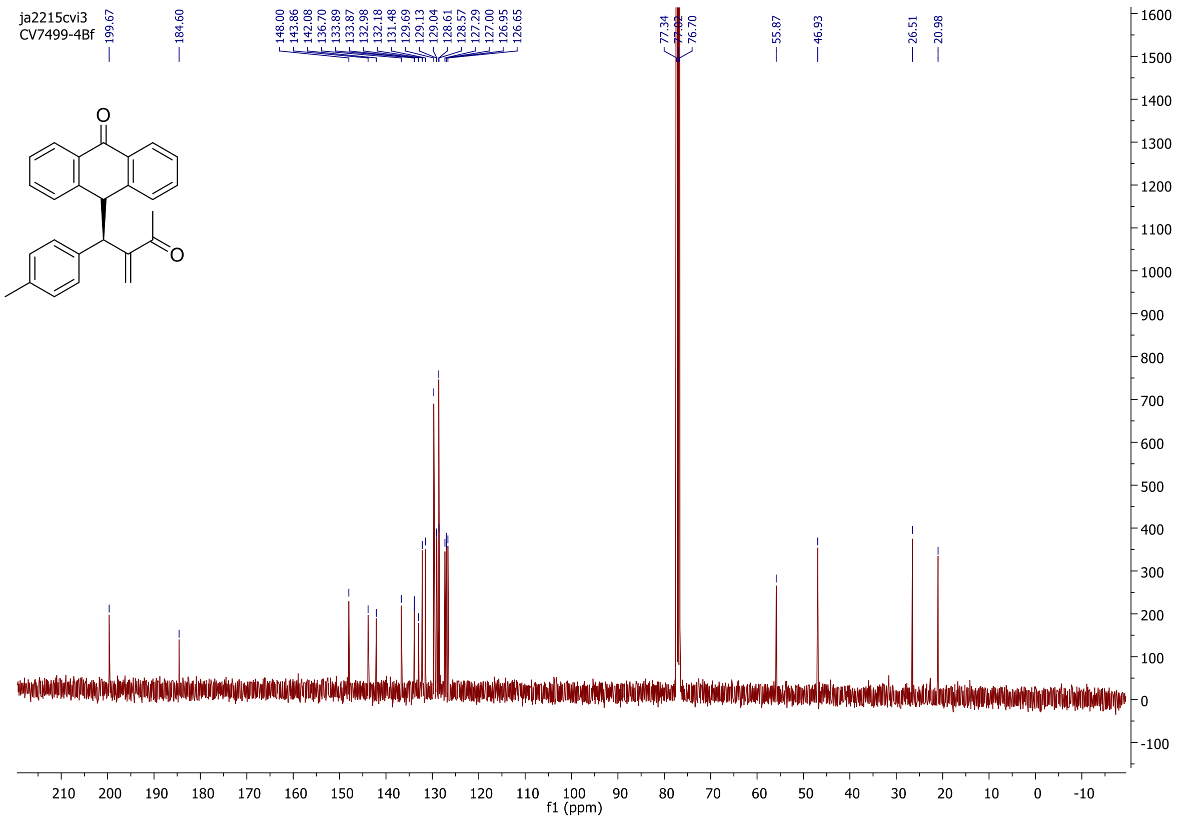

<u>(R)-10-(2-methylene-1-(4-nitrophenyl)-3-oxobutyl)anthracen-9(10H)-one</u> (4q)

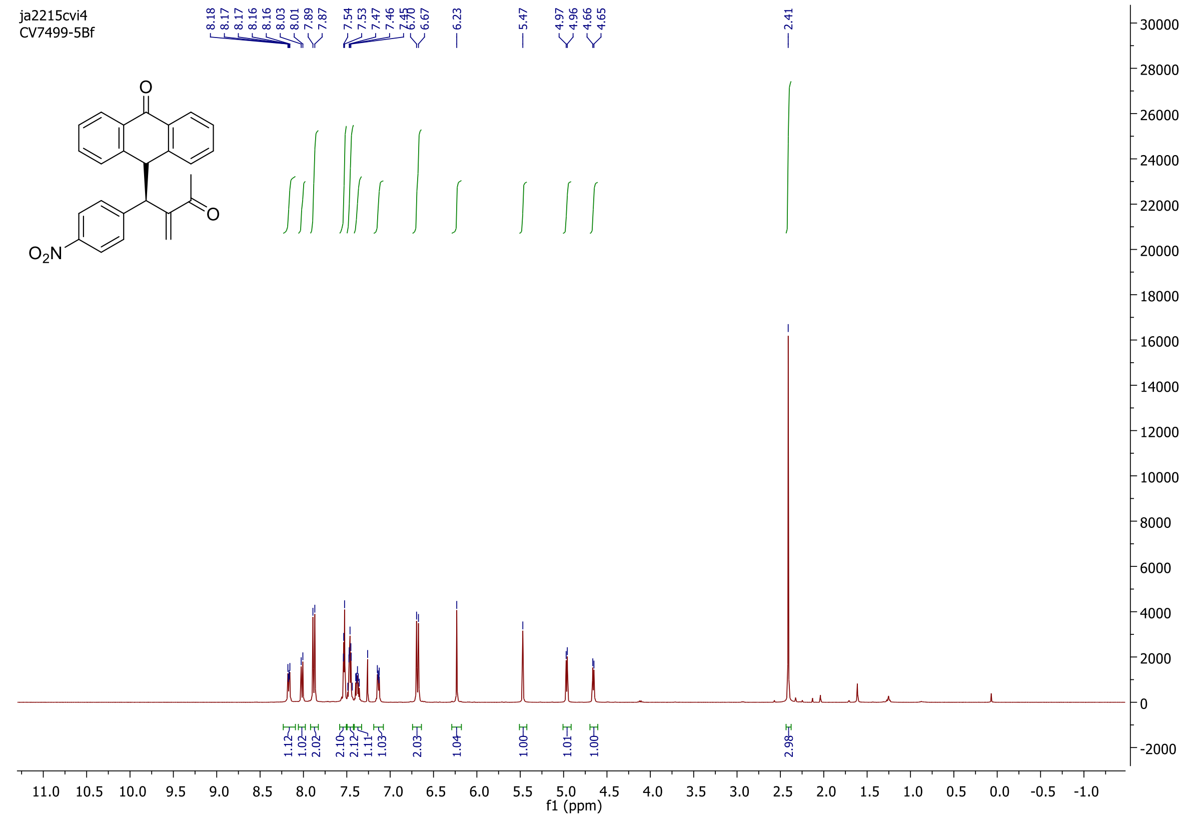

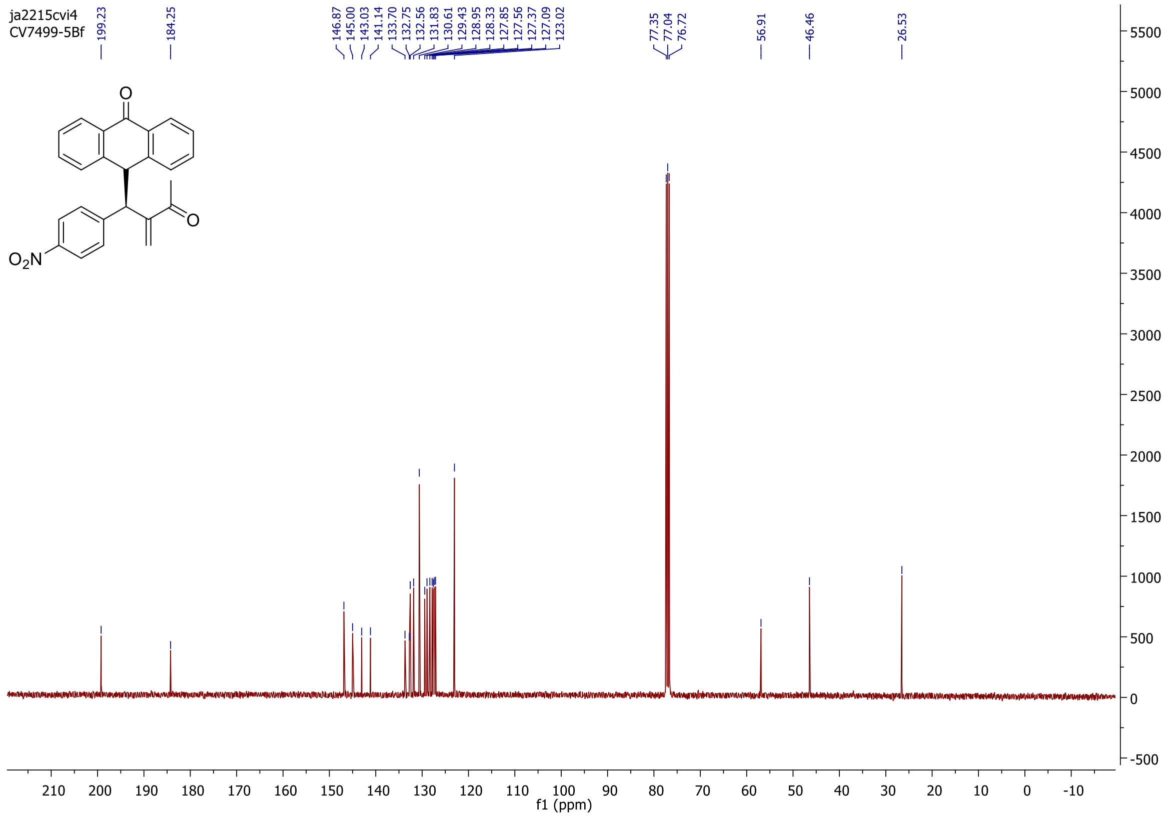

<u>(R)-10-(1-(4-bromophenyl)-2-methylene-3-oxobutyl)anthracen-9(10H)-one</u> (4r)

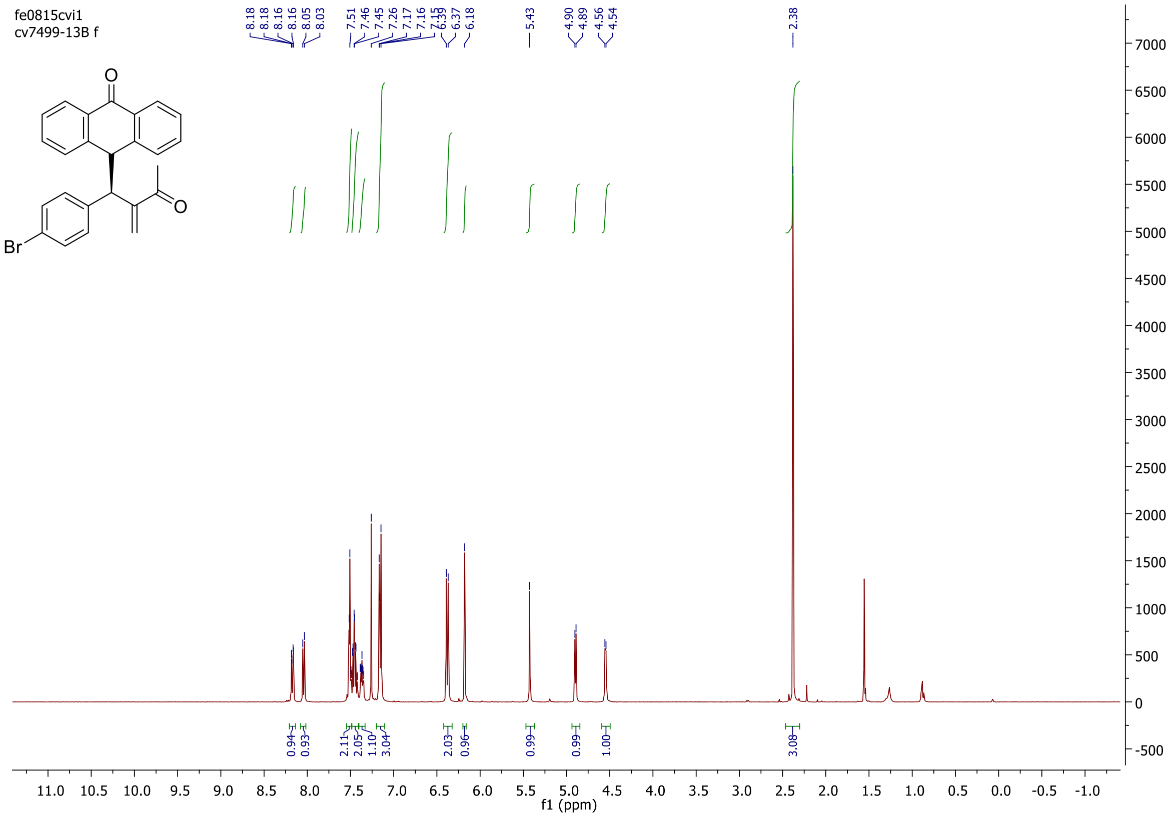

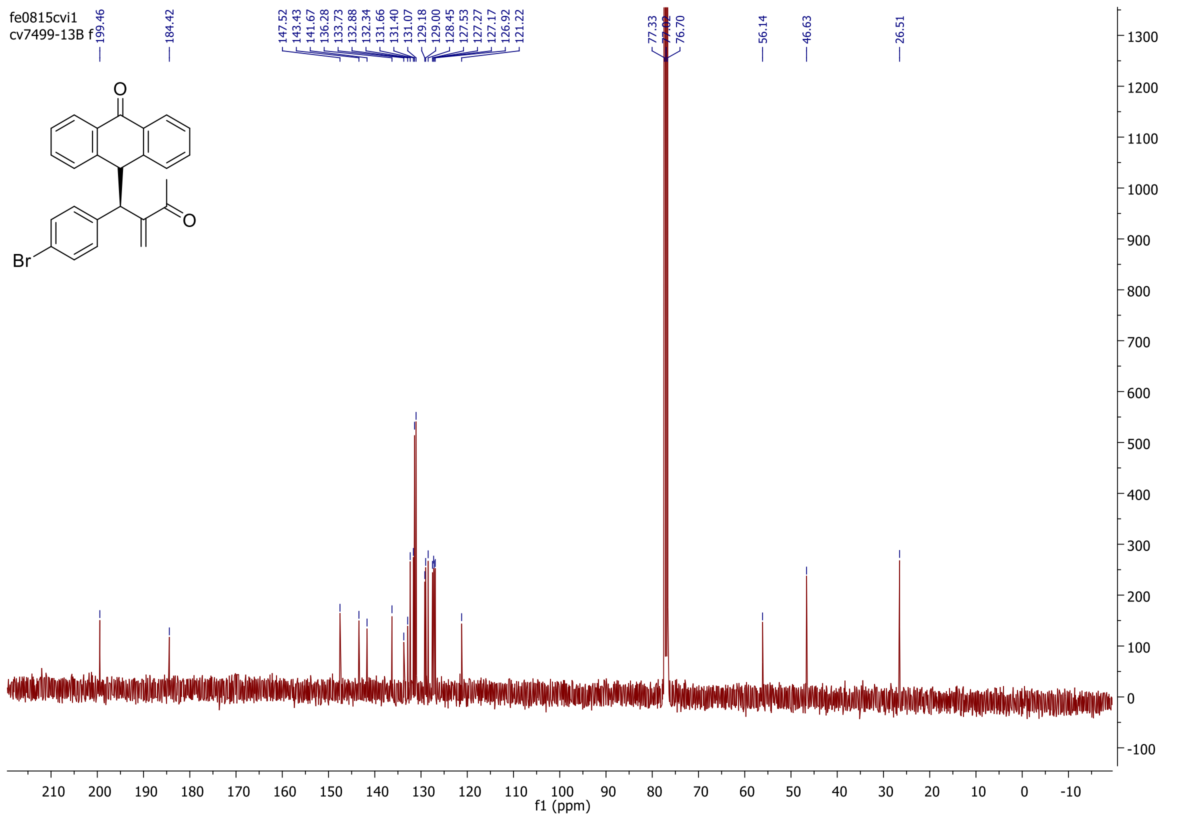

(R)-10-(1-(4-chlorophenyl)-2-methylene-3-oxobutyl)anthracen-9(10H)-one (4s)

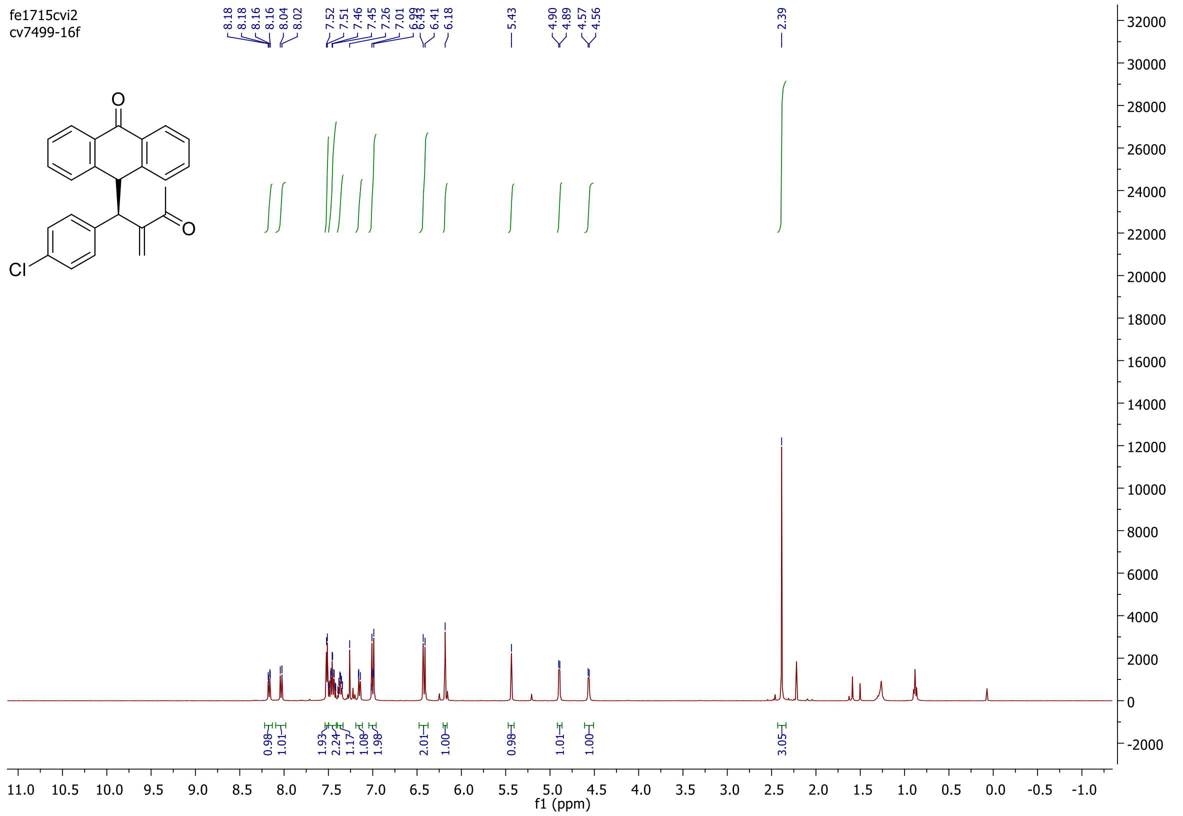

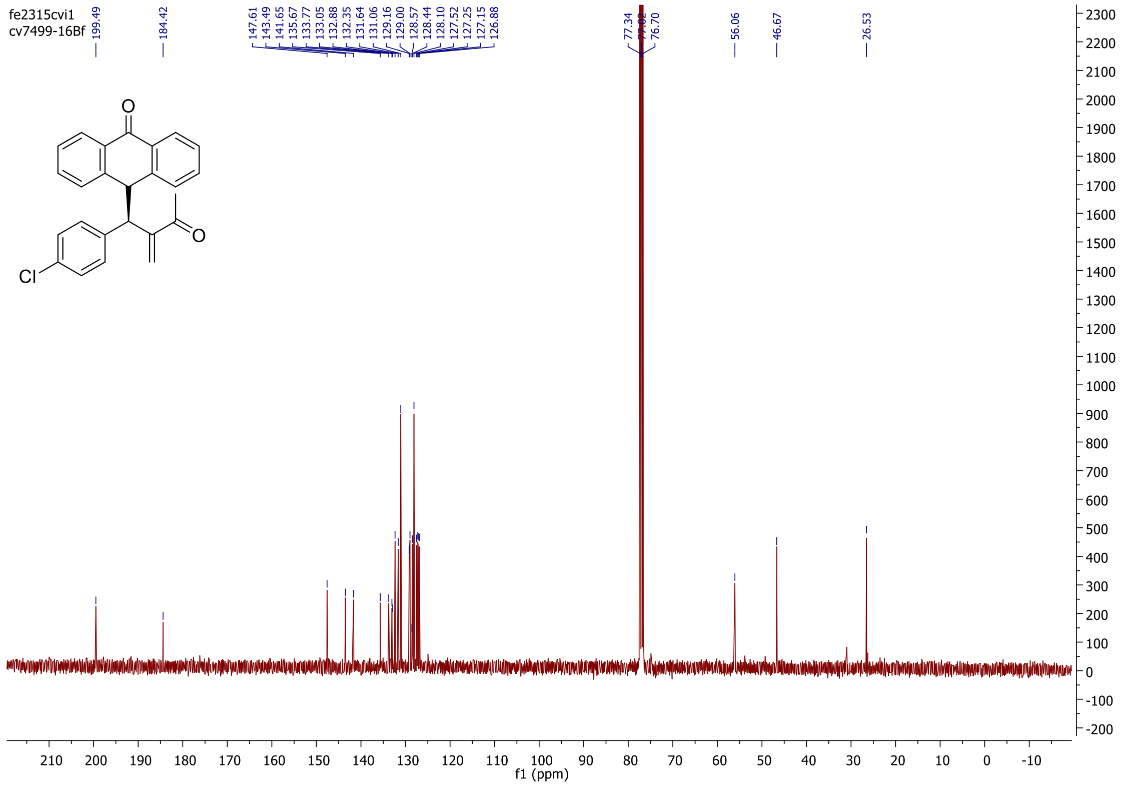

(R)-4-(2-methylene-3-oxo-1-(10-oxo-9,10-dihydroanthracen-9-yl)butyl)benzonitrile (4t)

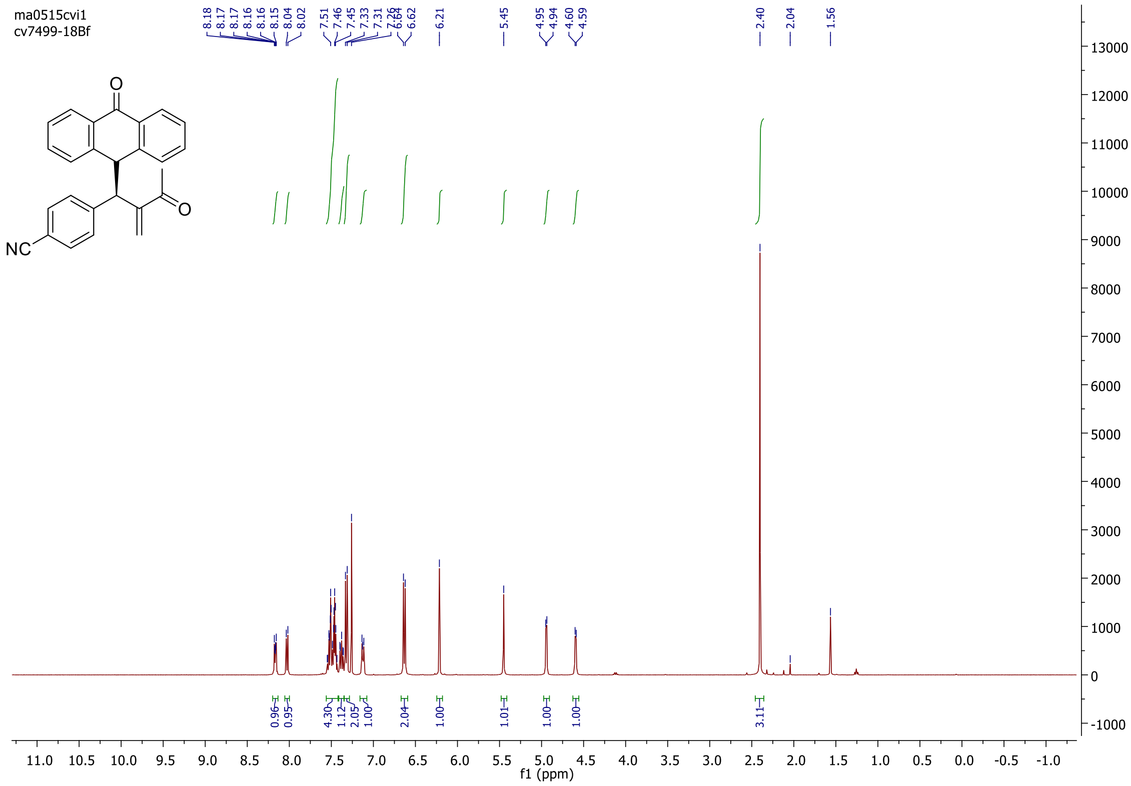

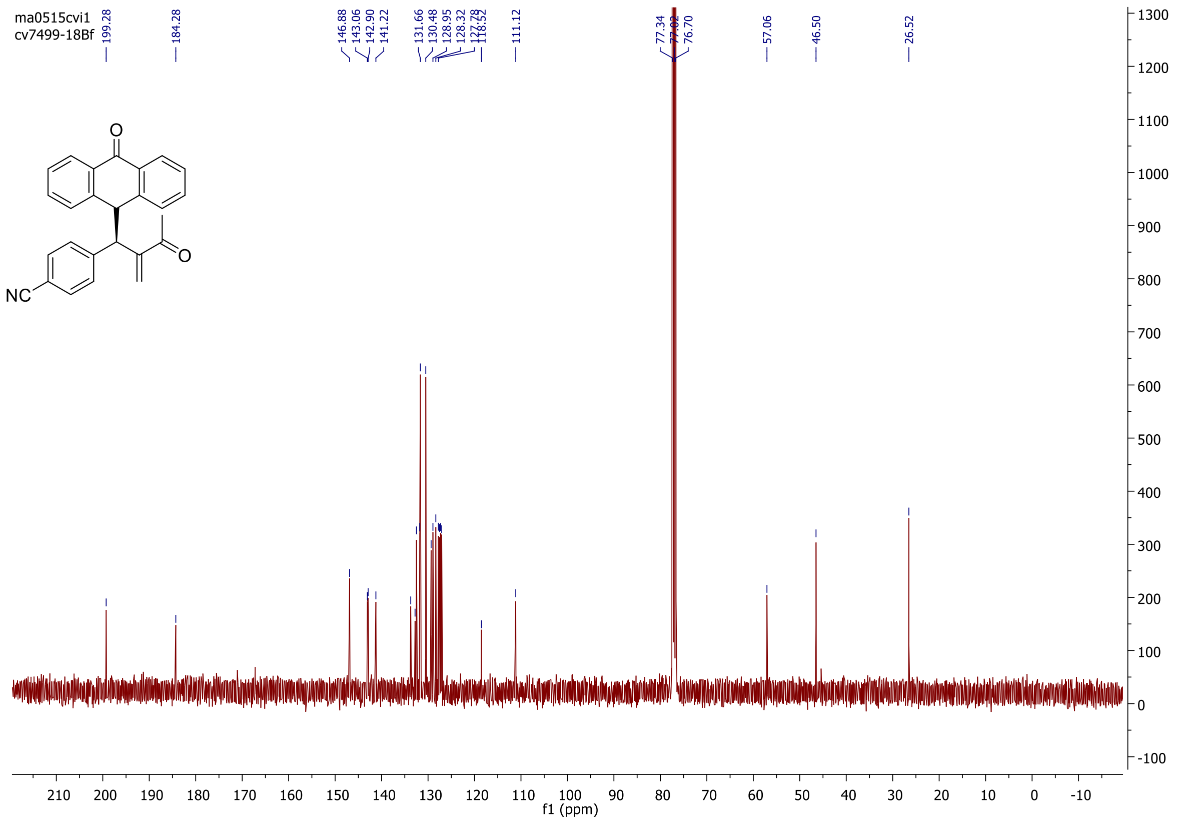

(R)-10-(2-methylene-3-oxo-1-phenylpentyl)anthracen-9(10H)-one (4u)

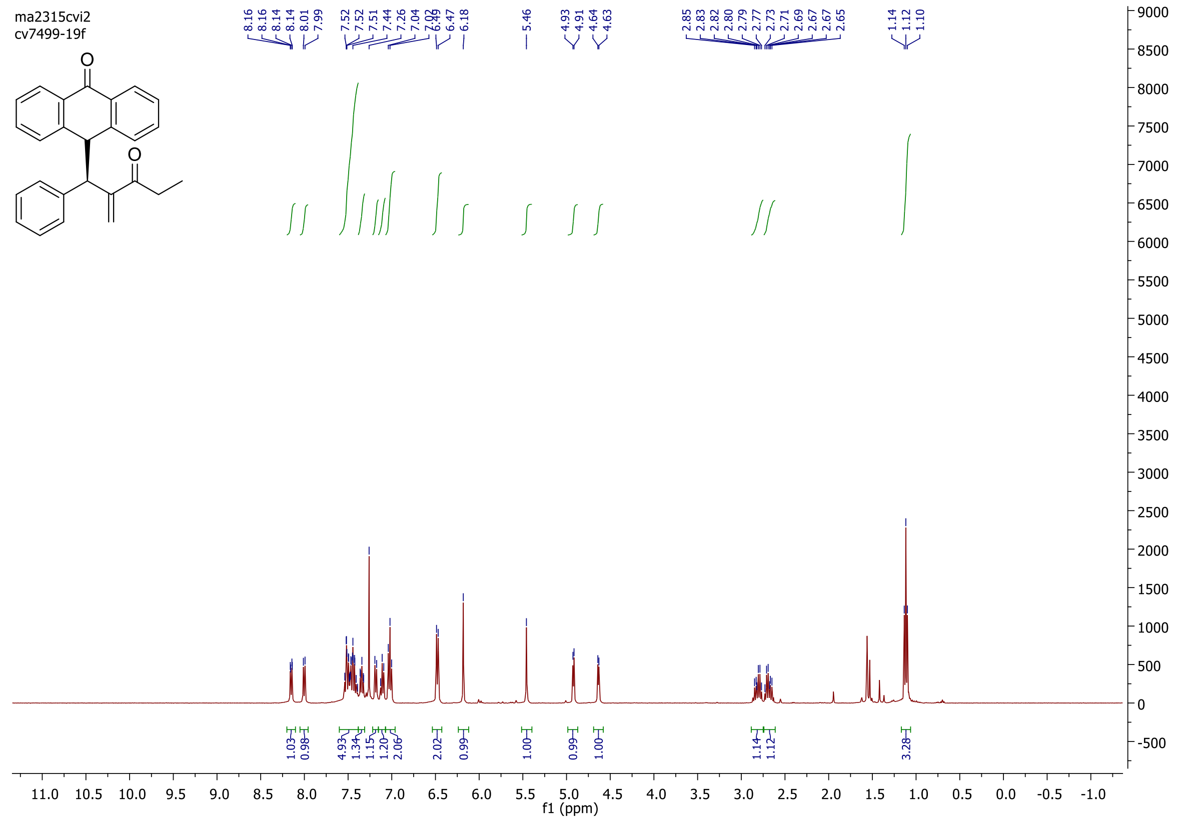

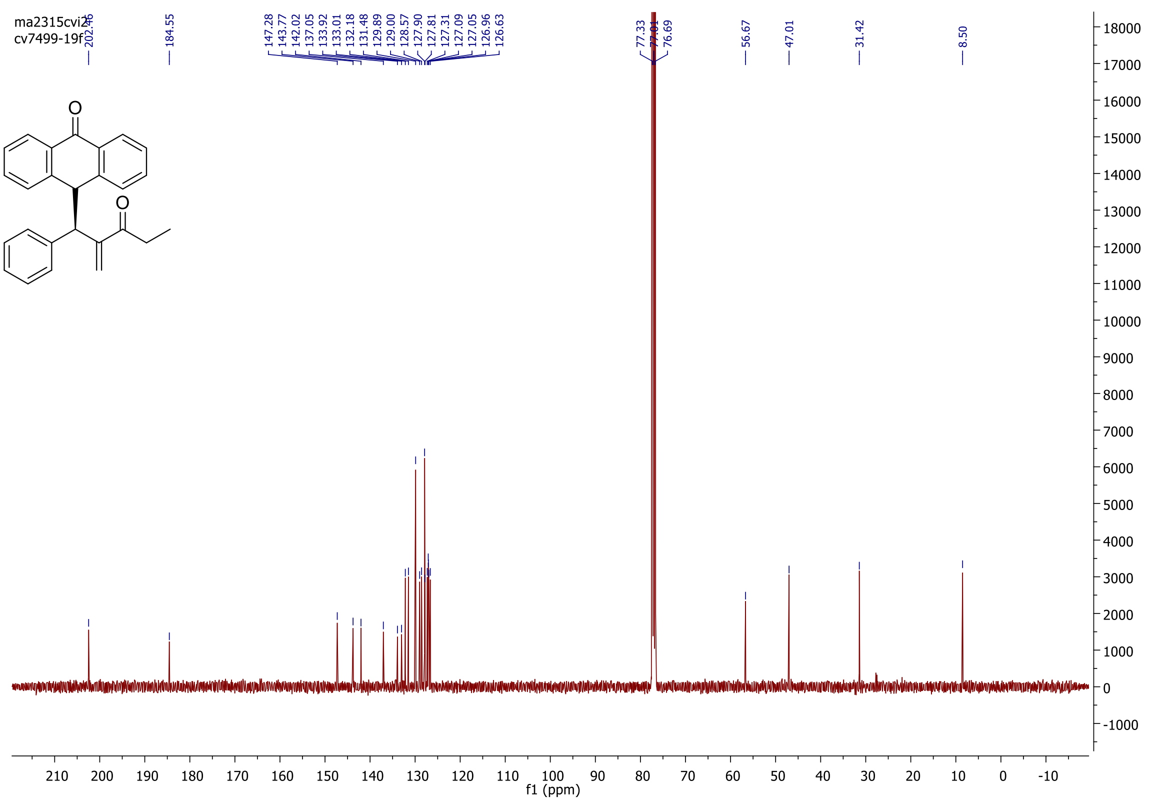
ma2315cvi2
cv7499-19f
202.96
184.55
147.28
143.77
142.02
137.05
133.92
133.01
132.18
131.48
129.89
129.00
128.57
127.90
127.81
127.31
127.09
127.05
126.96
126.63
77.33
77.01
76.69
56.67
47.01
31.42
8.50
f1 (ppm)

NMR Spectra for Hydrogenation

methyl (2S,3S)-3-(4-fluorophenyl)-2-methyl-3-(10-oxo-9,10-dihydroanthracen-9-yl)propanoate (5b)

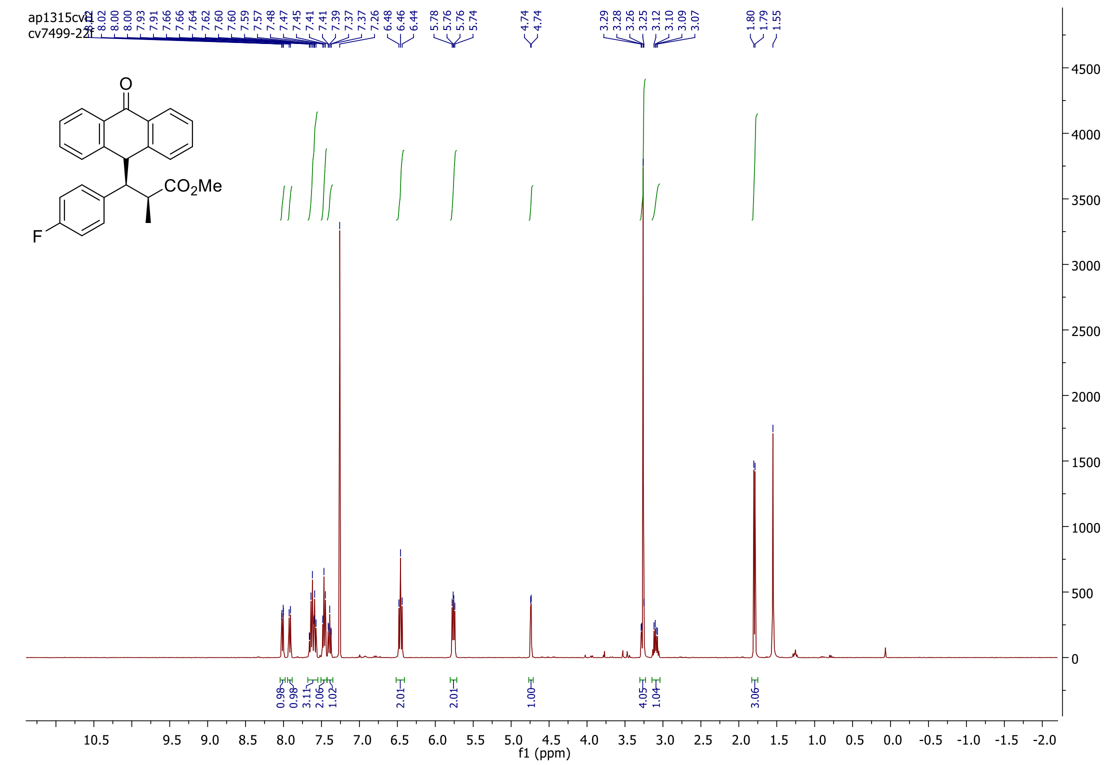

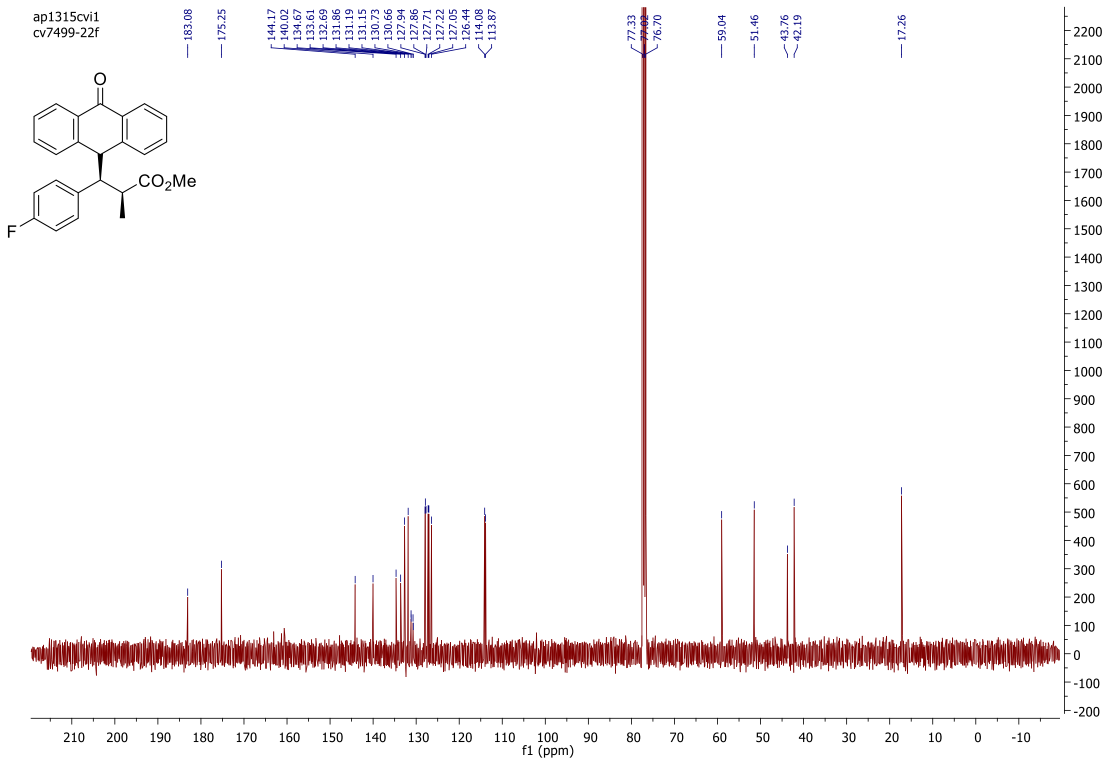

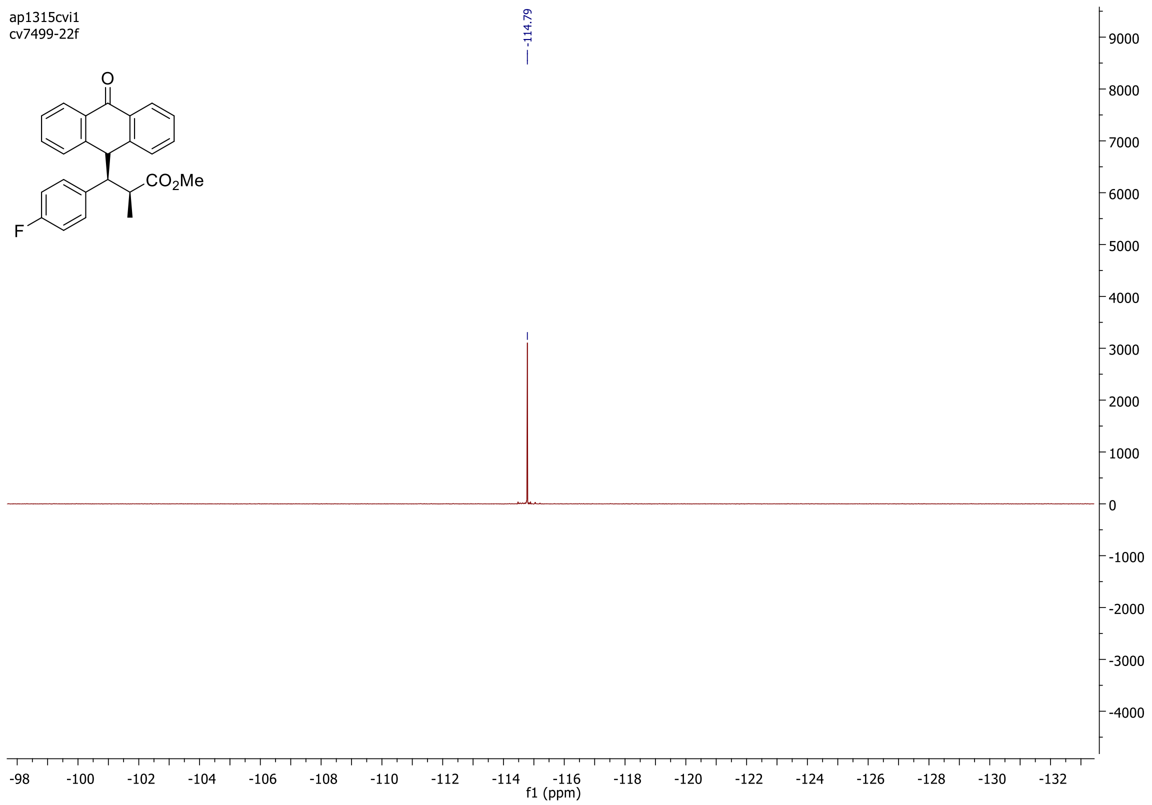

methyl (2S,3S)-3-(4-aminophenyl)-2-methyl-3-(10-oxo-9,10-dihydroanthracen-9-yl)propanoate (5c)

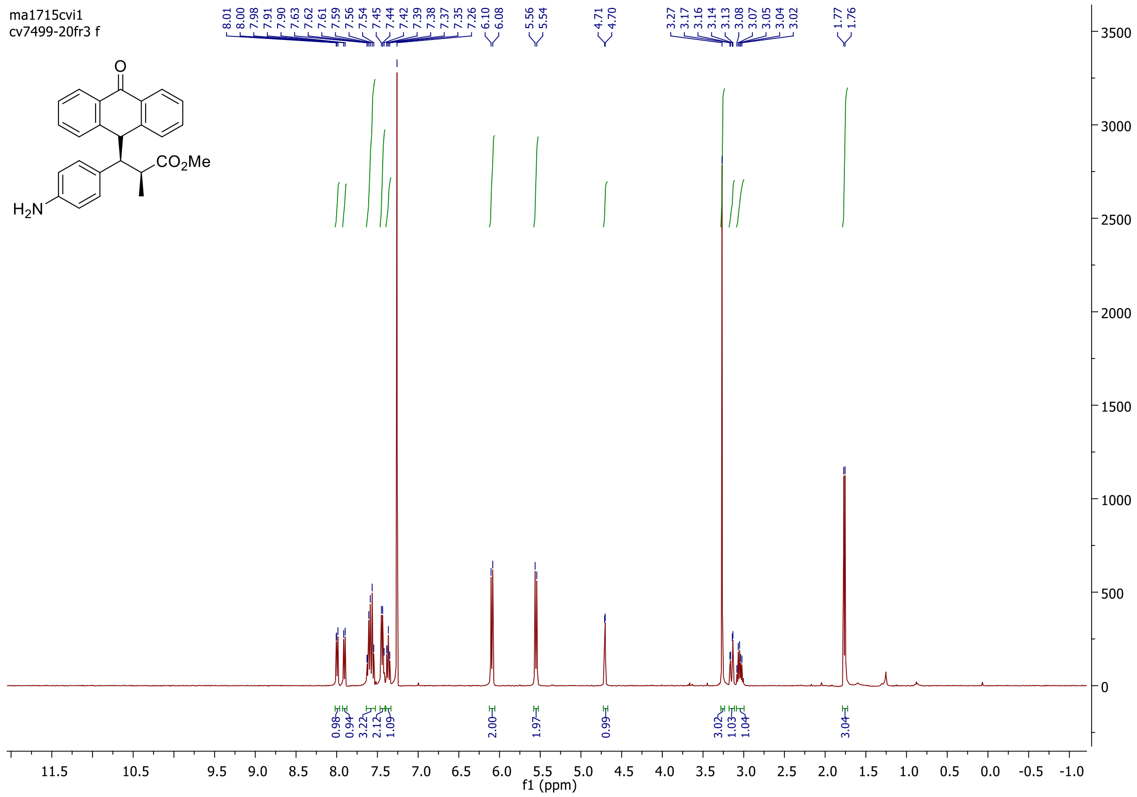

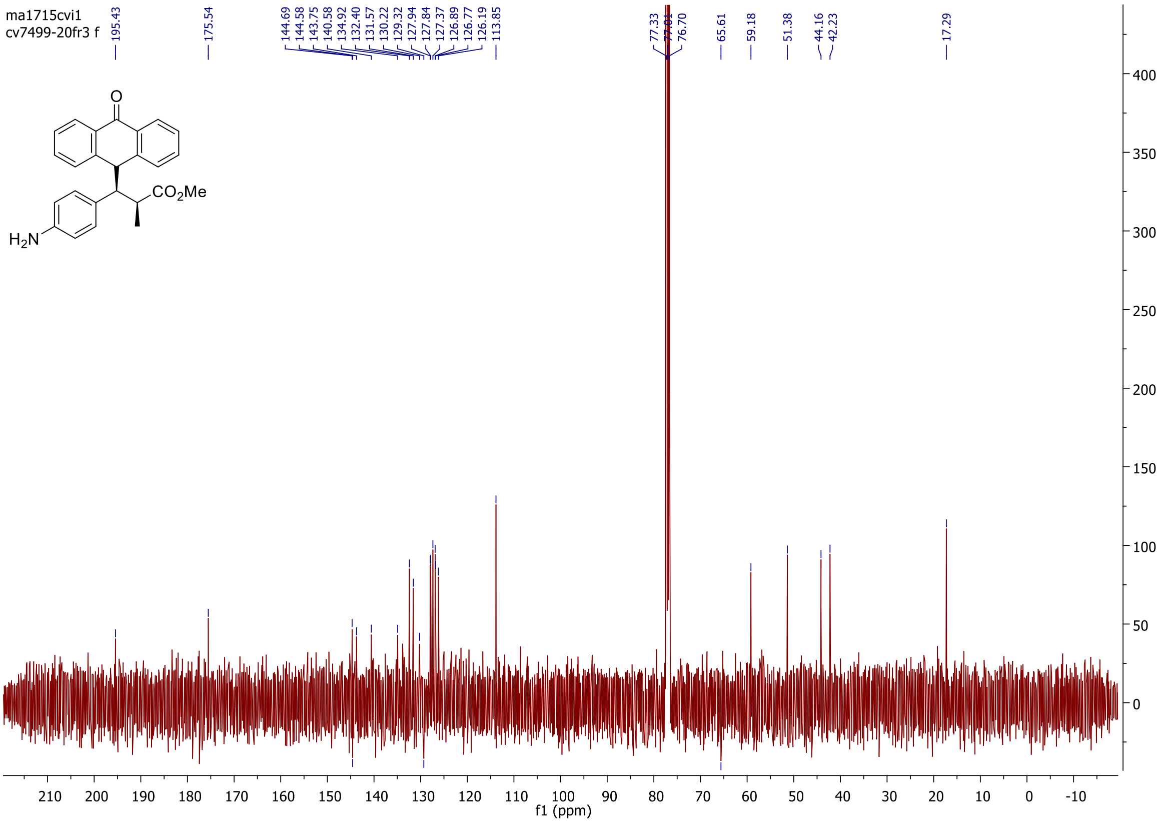

methyl (2S,3S)-2-methyl-3-(10-oxo-9,10-dihydroanthracen-9-yl)-3-(p-tolyl)propanoate (5d)

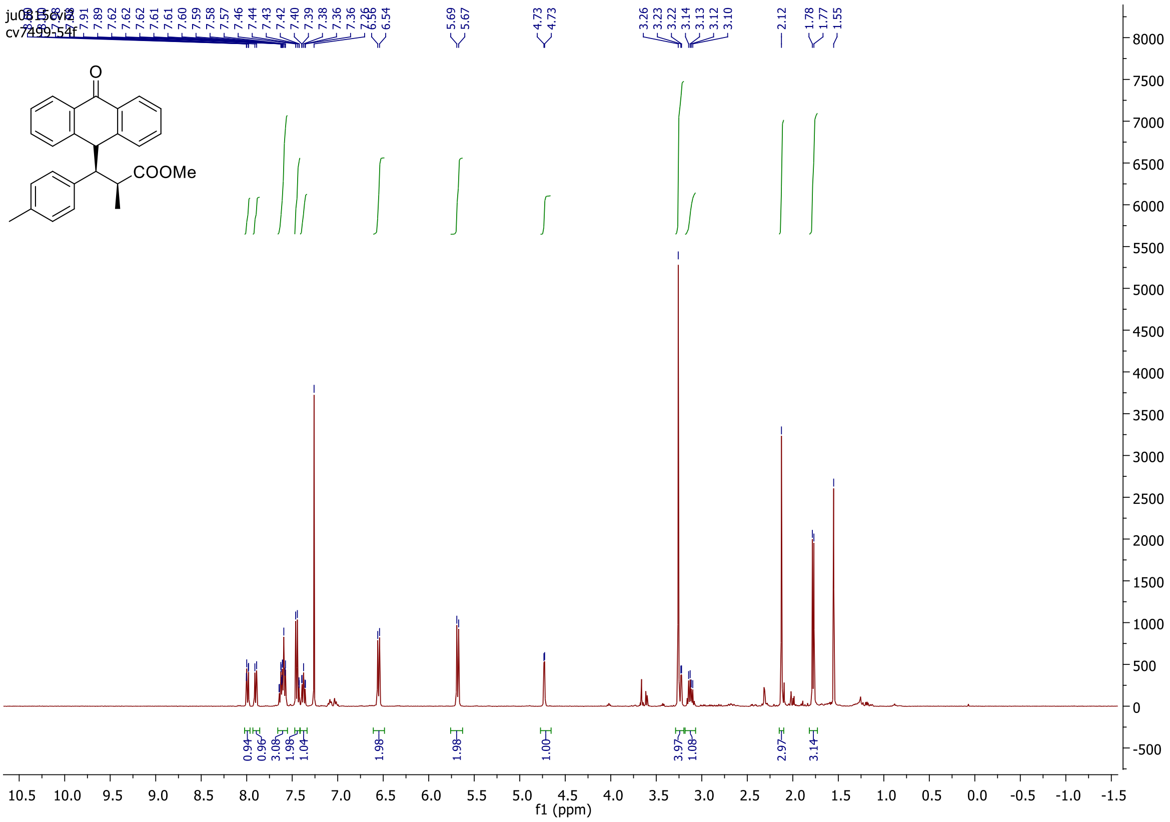

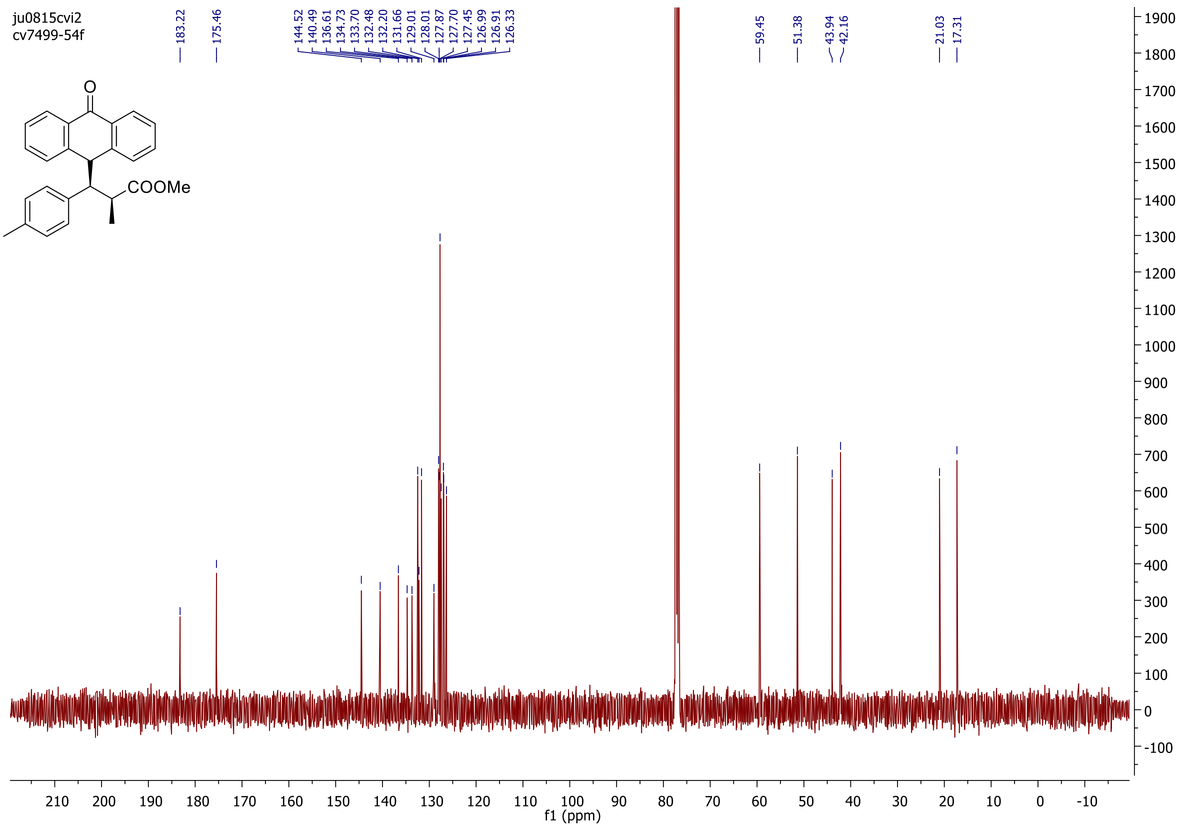

(2S,3S)-2-methyl-3-(10-oxo-9,10-dihydroanthracen-9-yl)-3-phenylpropanenitrile (5m)

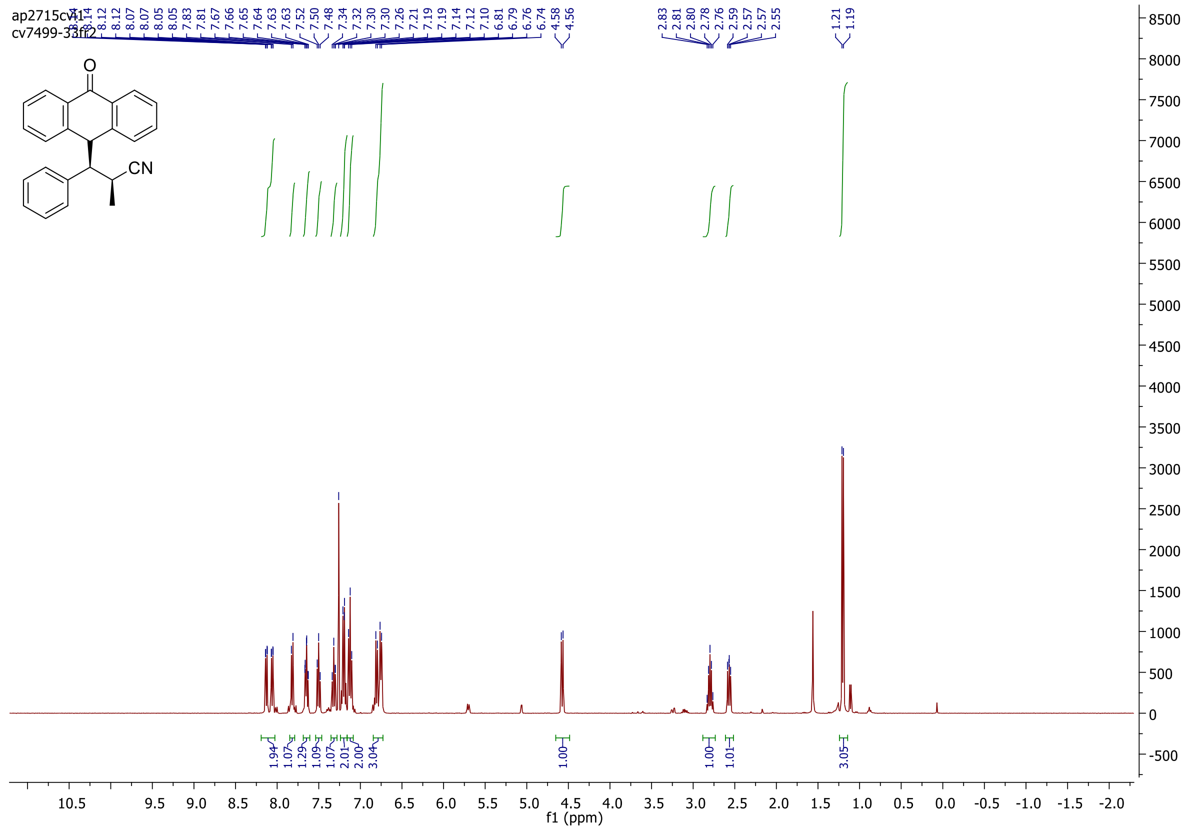

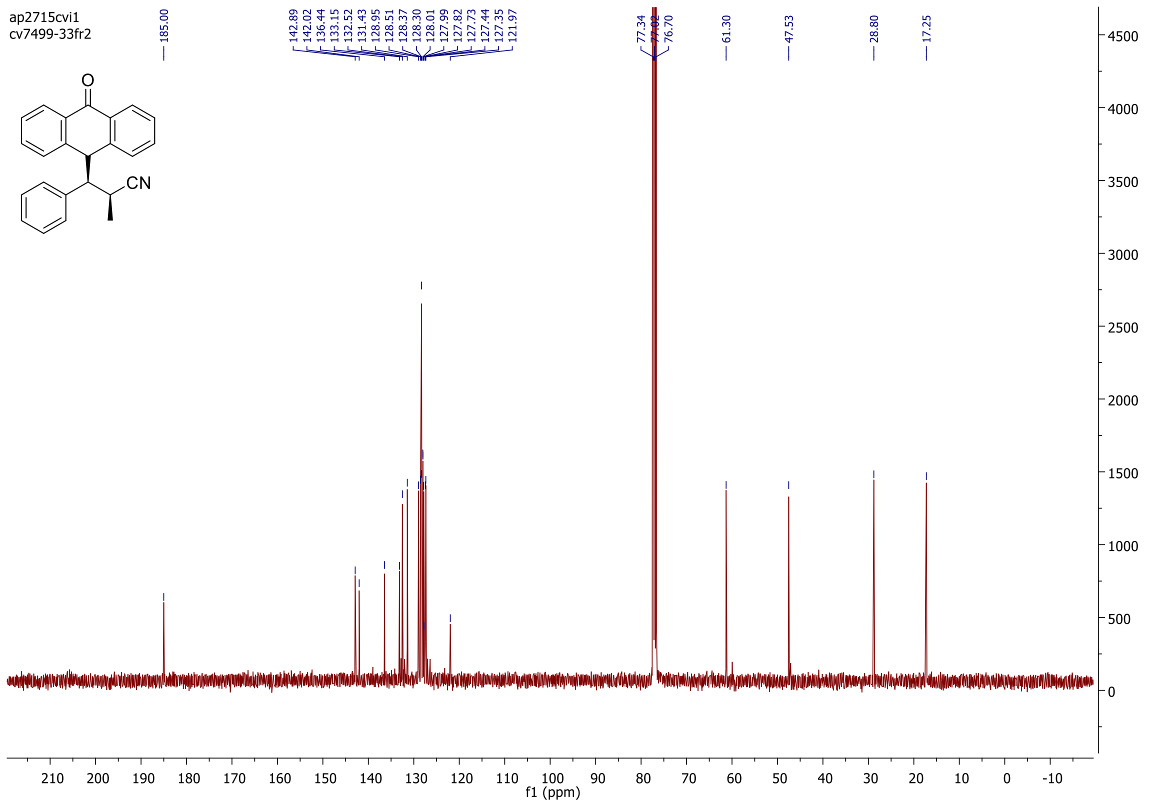

10-((1S,2S)-1-(4-bromophenyl)-2-methyl-3-oxobutyl)anthracen-9(10H)-one (5r)

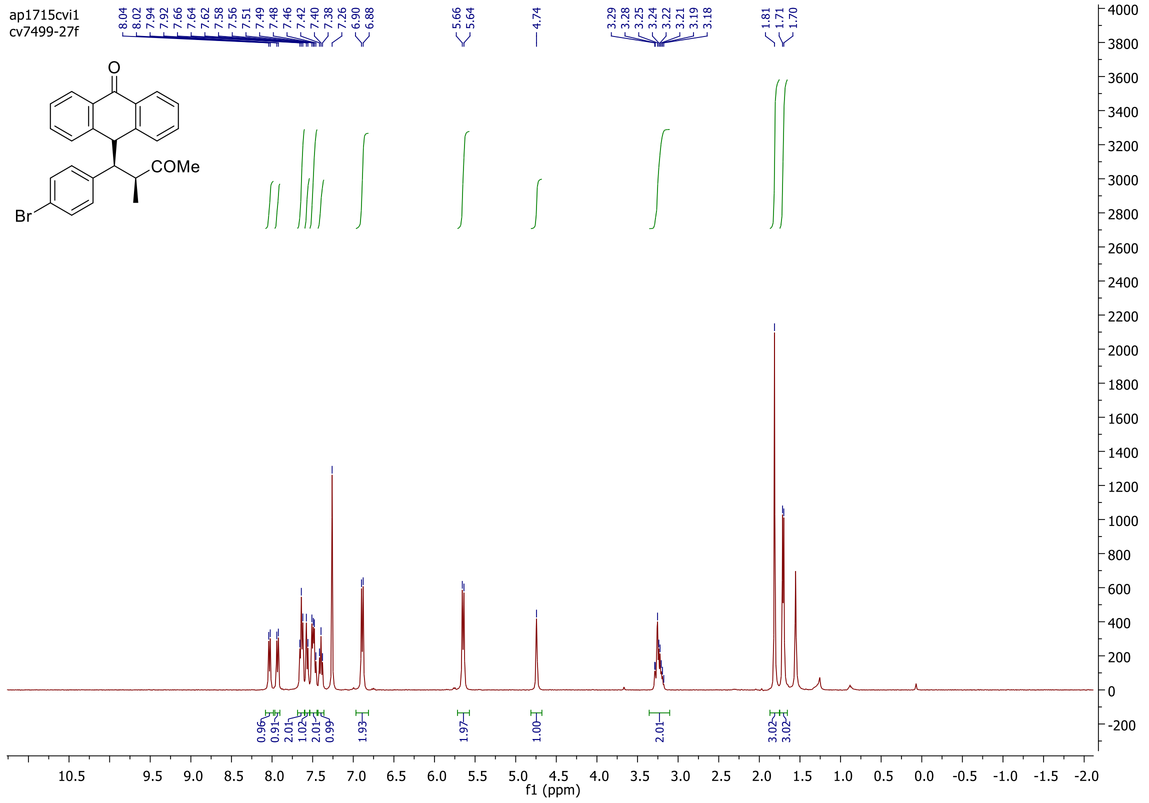

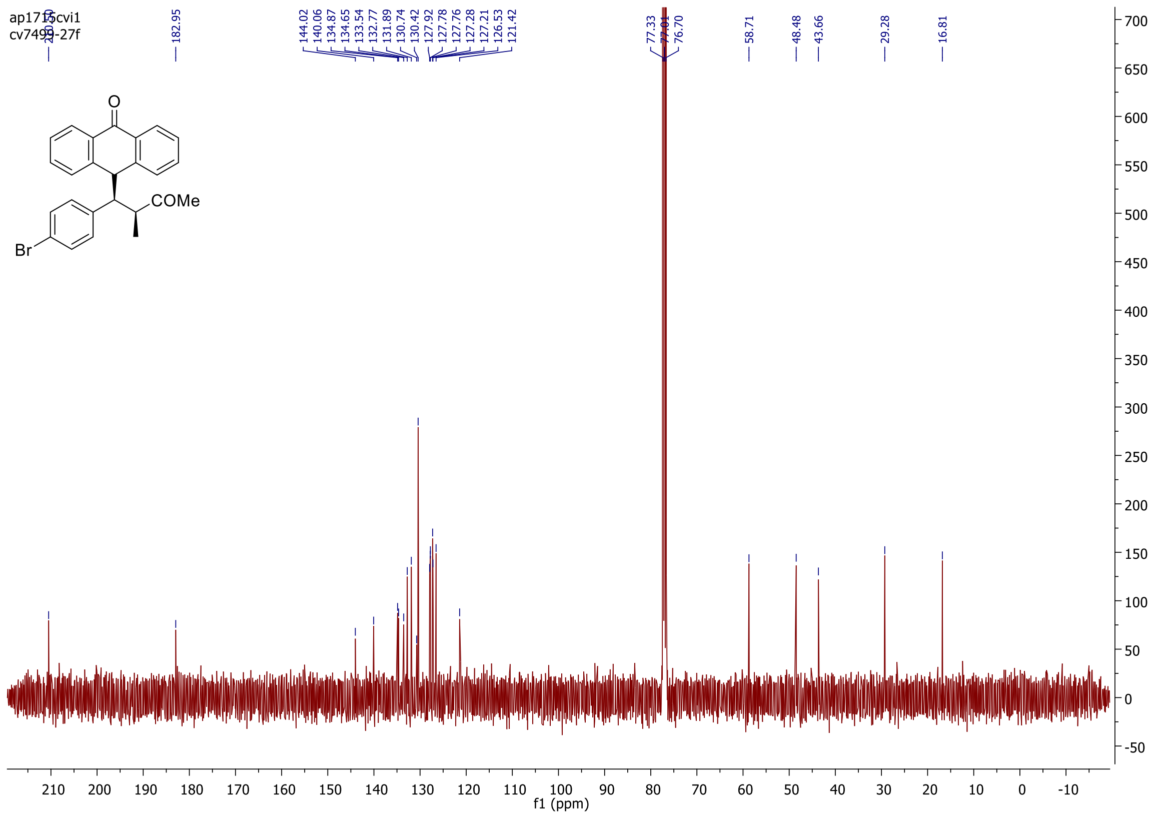

10-((1S,2S)-2-methyl-3-oxo-1-phenylpentyl)anthracen-9(10H)-one (5u)

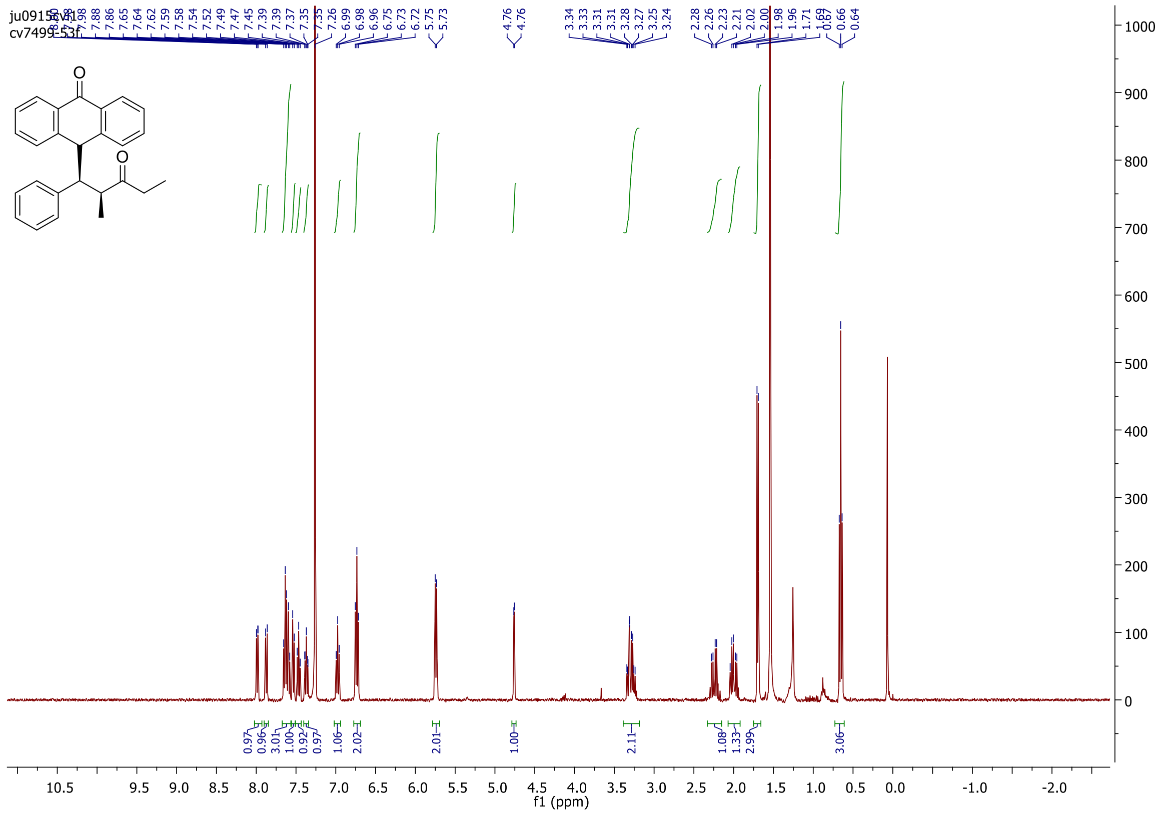

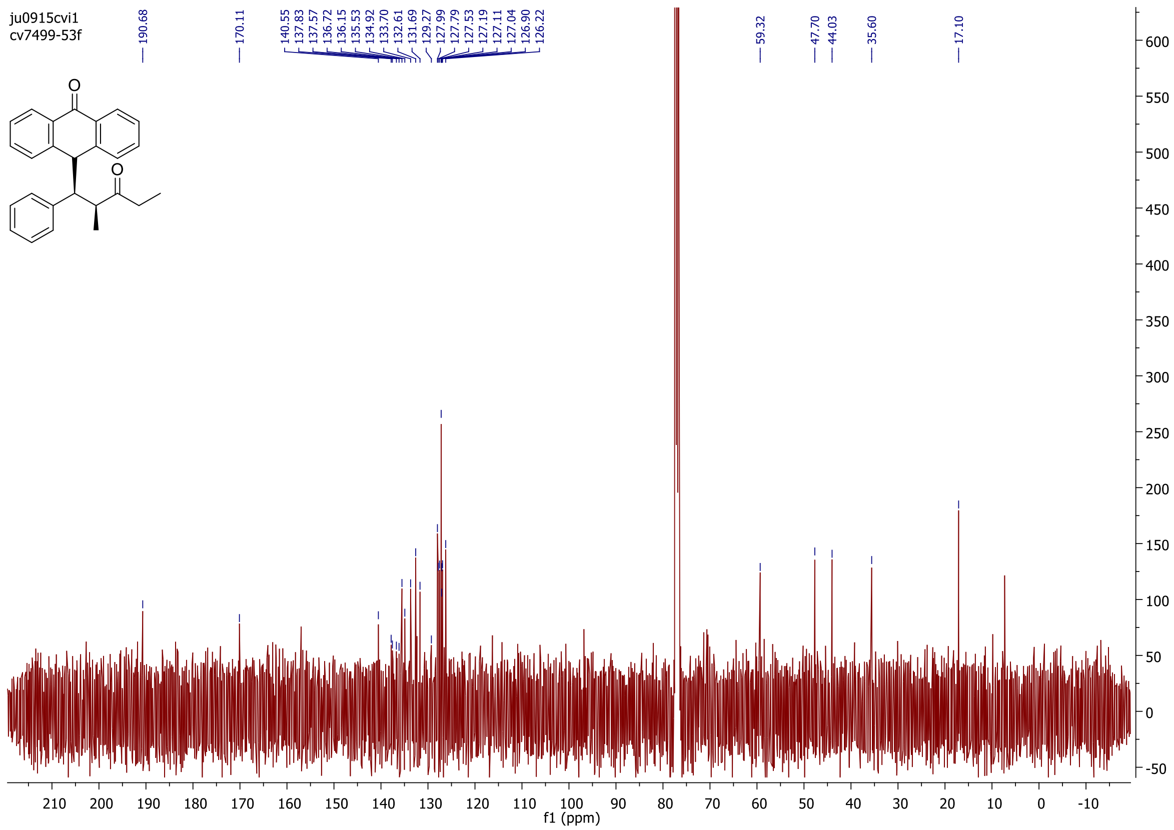
ju0915cvi1
cv7499-53f
190.68
170.11
140.55
137.83
137.57
136.72
136.15
135.53
134.92
133.70
132.61
131.69
129.27
127.99
127.79
127.53
127.19
127.11
127.04
126.90
126.22
59.32
47.70
44.03
35.60
17.10
f1 (ppm)

HPLC Chiral data

<u>methyl (R)-2-((10-oxo-9,10-dihydroanthracen-9-yl)(phenyl)methyl)acrylate</u> (4a)

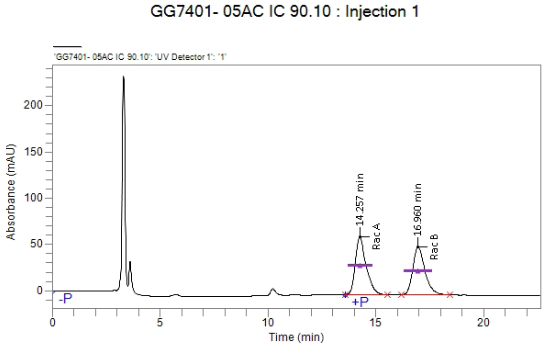

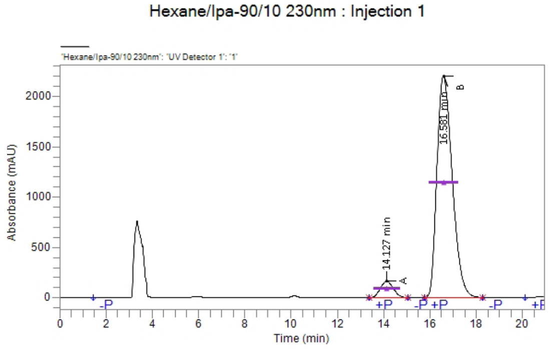

Time	Area	Area %
14.257	2,175,183.1	51.99
16.960	2,008,845.4	48.01
**Total**	4,184,028.6	100.00

Time	Area	Area %
14.127	6,436,097.4	5.95
16.581	101,665,240.9	94.05
**Total**	108,101,338.2	100.00

methyl (R)-2-((4-fluorophenyl)(10-oxo-9,10-dihydroanthracen-9-yl)methyl)acrylate (4b)

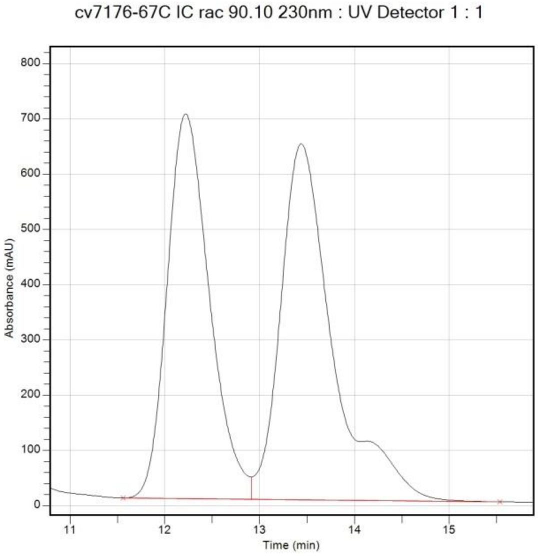

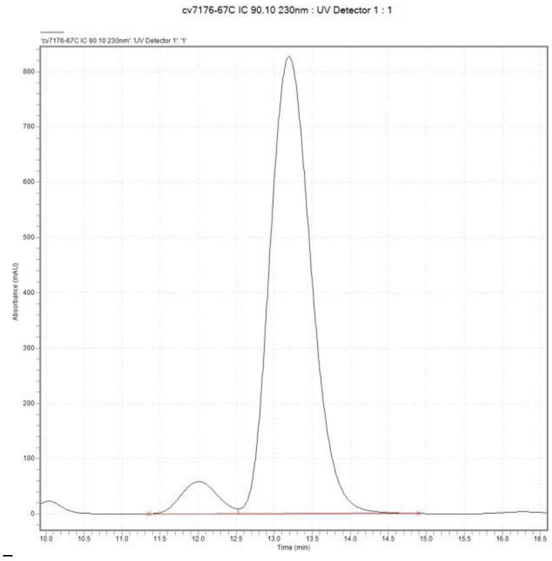

Time	Area	Area %
12.219	21,545,030.6	46.69
13.438	24,597,981.8	53.31
**Total**	46,143,012.4	100.00

Time	Area	Area %
12.010	1,945,432.6	5.91
13.194	30,964,687.1	94.09
**Total**	32,910,119.7	100.00

methyl (R)-2-((4-nitrophenyl)(10-oxo-9,10-dihydroanthracen-9-yl)methyl)acrylate (4c)

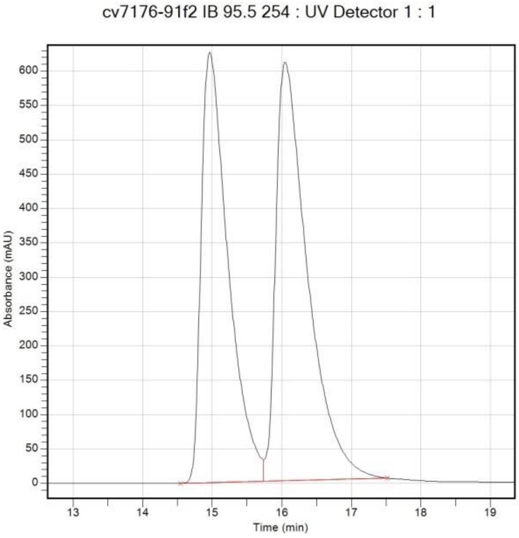

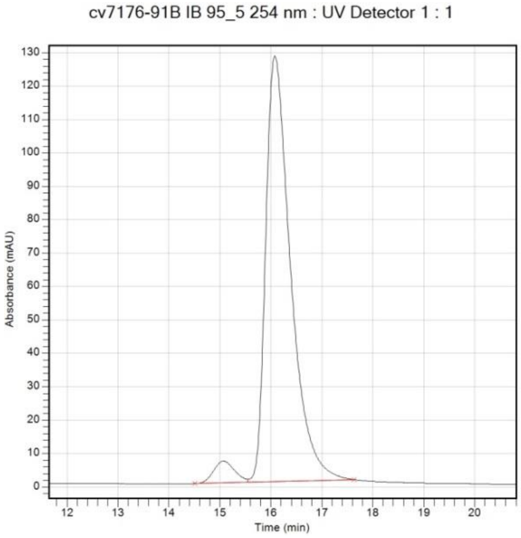

Time	Area	Area %
14.963	16,902,273.0	46.57
16.048	19,394,894.4	53.43
Total	36,297,167.5	100.00

Time	Area	Area %
15.068	188,908.1	4.19
16.076	4,322,708.4	95.81
Total	4,511,616.5	100.00

methyl (R)-2-((10-oxo-9,10-dihydroanthracen-9-yl)(p-tolyl)methyl)acrylate (4d)

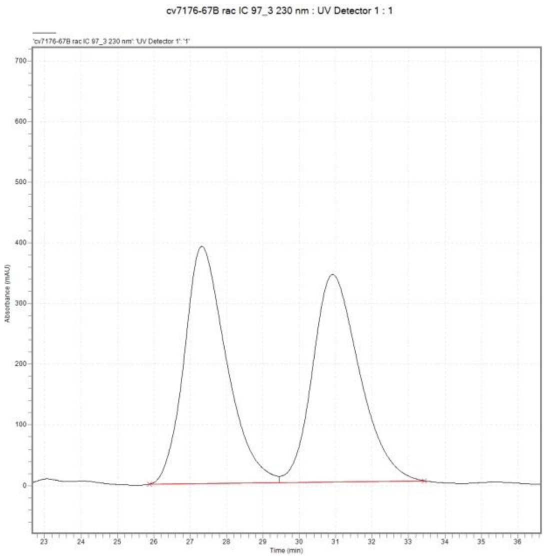

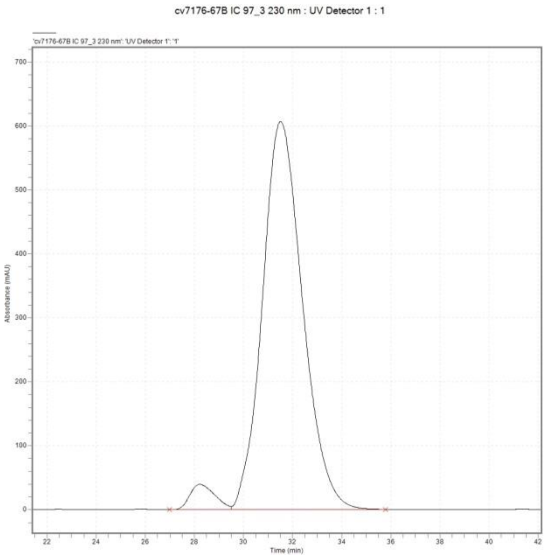

Time	Area	Area %
27.319	31,454,409.0	50.67
30.915	30,617,895.8	49.33
**Total**	62,072,304.8	100.00

Time	Area	Area %
28.219	2,796,359.7	3.91
31.510	68,794,592.0	96.09
**Total**	71,590,951.7	100.00

methyl (R)-2-((4-chlorophenyl)(10-oxo-9,10-dihydroanthracen-9-yl)methyl)acrylate (4e)

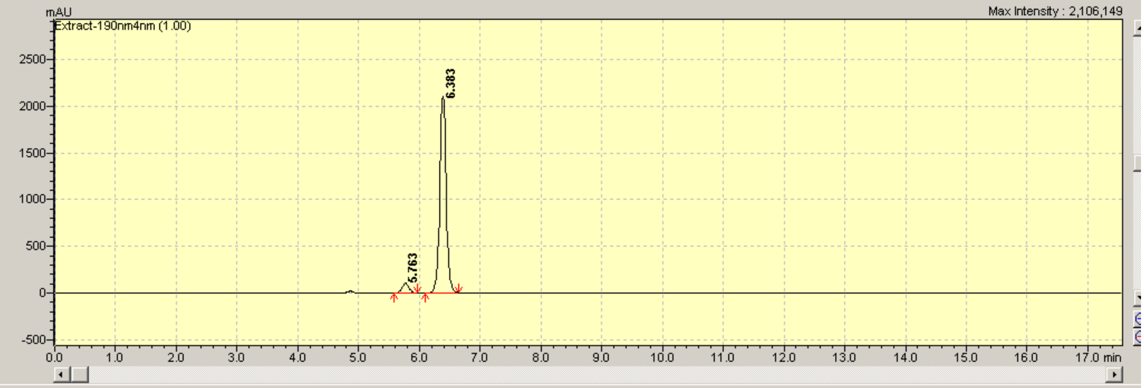

Peak Table

Peak#	ID#	Name	Ret. Time	Conc.	Area	Height	Similarity Index	Mark	Peak Start	Peak End	Area%
1			5.763	5.12934	857679	105734	0.000000		5.589	5.963	5.1293
2			6.383	94.87066	15863356	2103461	0.000000		6.101	6.645	94.8707

Extracted Chromatogram 190nm | Ch1 254nm

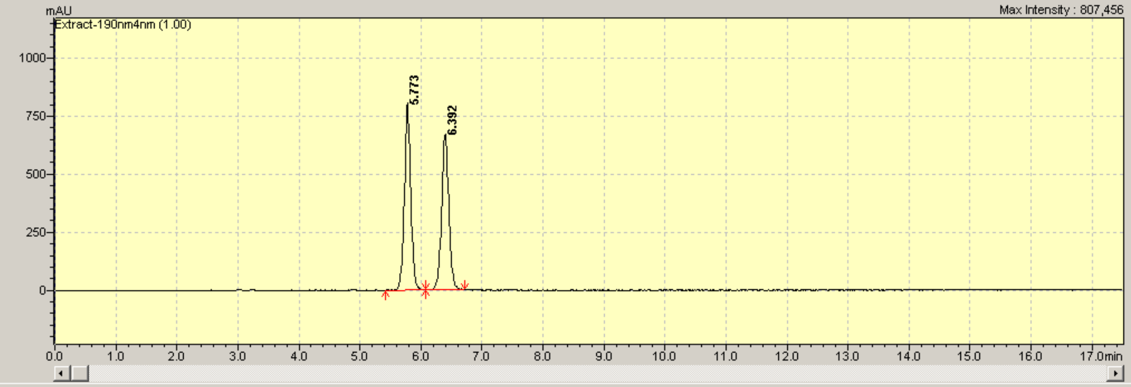

Peak Table

Peak#	ID#	Name	Ret. Time	Conc.	Area	Height	Similarity Index	Mark	Peak Start	Peak End	Area%
1			5.773	51.56986	5788182	805399	0.000000		5.419	6.069	51.569
2			6.392	48.43014	5435781	670505	0.000000		6.080	6.720	48.430

Extracted Chromatogram 190nm | Ch1 254nm

methyl (R)-2-((3-chlorophenyl)(10-oxo-9,10-dihydroanthracen-9-yl)methyl)acrylate (4f)

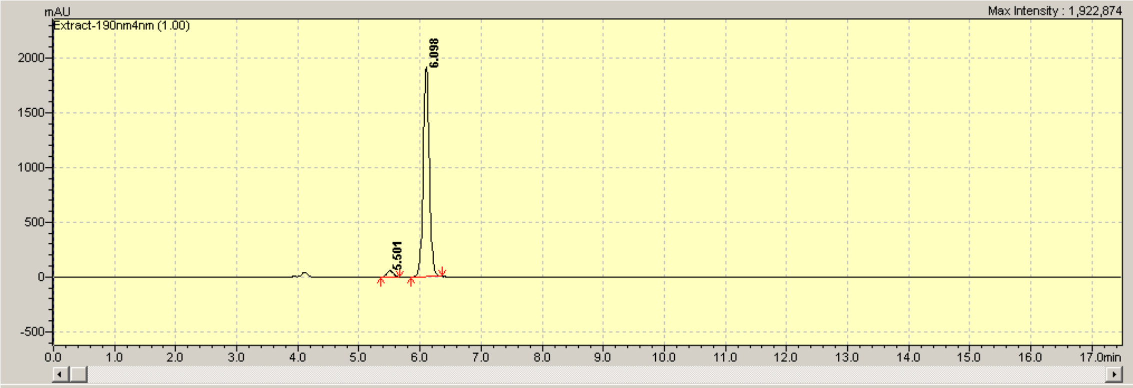

Peak Table

Peak#	ID#	Name	Ret. Time	Conc.	Area	Height	Similarity Index	Mark	Peak Start	Peak End	Area%
1			5.501	3.16568	440415	57275	0.000000		5.355	5.664	3.1657
2			6.098	96.83432	13471753	1921811	0.000000		5.845	6.357	96.8343

Extracted Chromatogram 190nm | Ch1 254nm

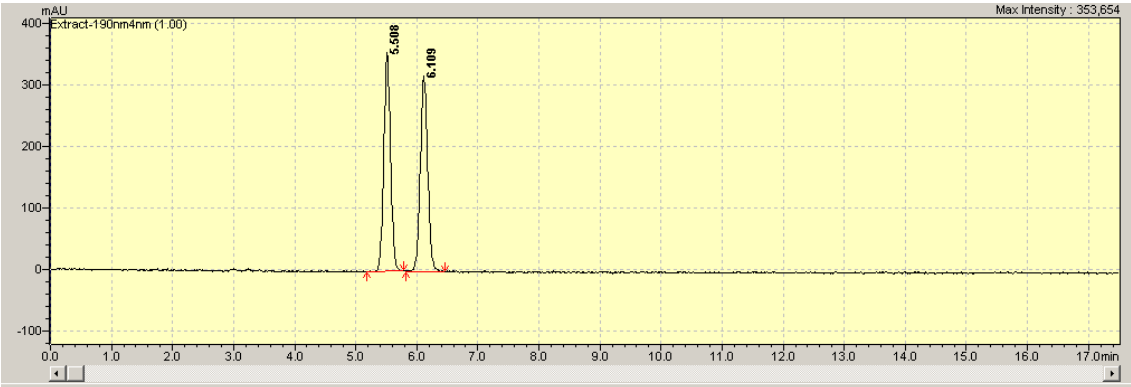

Peak Table

Peak#	ID#	Name	Ret. Time	Conc.	Area	Height	Similarity Index	Mark	Peak Start	Peak End	Area%
1			5.508	50.28861	2739312	356892	0.000000		5.184	5.781	50.2886
2			6.109	49.71139	2707870	318549	0.000000		5.824	6.464	49.7114

Extracted Chromatogram 190nm | Ch1 254nm

methyl (S)-2-((2-chlorophenyl)(10-oxo-9,10-dihydroanthracen-9-yl)methyl)acrylate (4g)

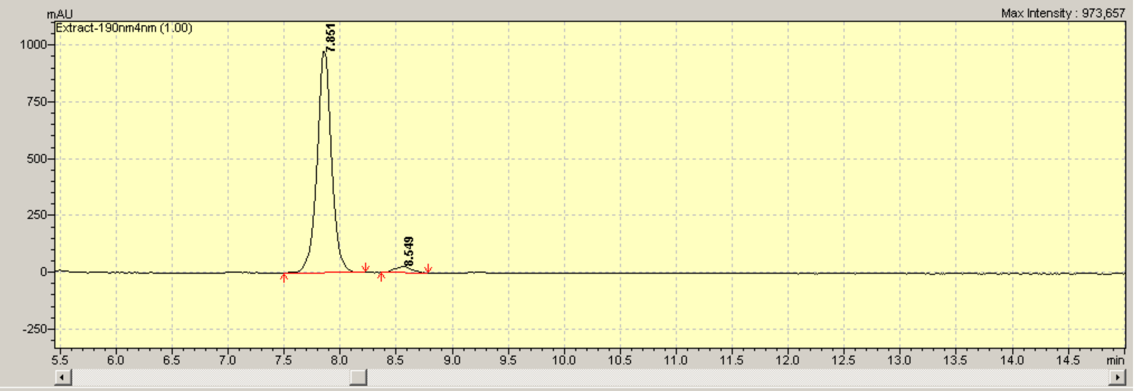

Peak Table

Peak#	ID#	Name	Ret. Time	Conc.	Area	Height	Similarity Index	Mark	Peak Start	Peak End	Area%
1			7.851	97.01453	8674611	974498	0.000000		7.499	8.224	97.0145
2			8.549	2.98547	266947	25658	0.000000		8.363	8.789	2.9855

Extracted Chromatogram 190nm / Ch1 254nm

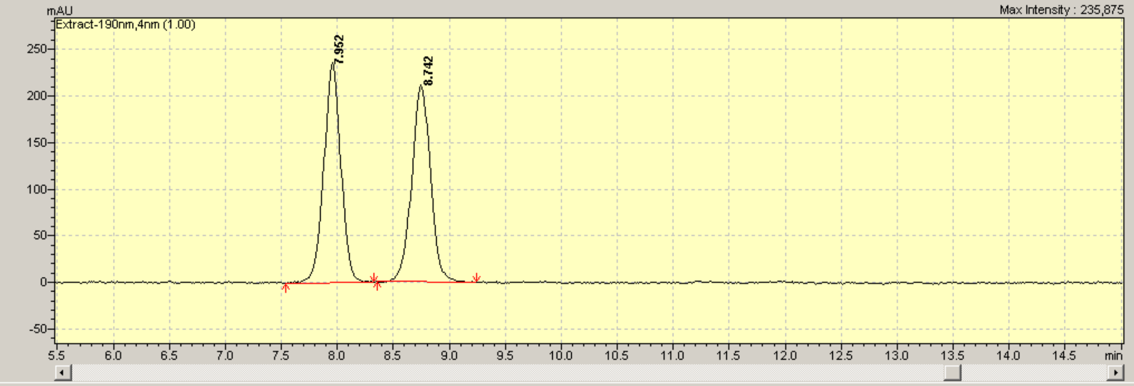

Peak Table

Peak#	ID#	Name	Ret. Time	Conc.	Area	Height	Similarity Index	Mark	Peak Start	Peak End	Area%
1			7.952	50.36321	2501024	236134	0.000000		7.541	8.331	50.3632
2			8.742	49.63679	2464950	211794	0.000000		8.352	9.248	49.6368

Extracted Chromatogram 190nm / Ch1 254nm

methyl (R)-2-((4-bromophenyl)(10-oxo-9,10-dihydroanthracen-9-yl)methyl)acrylate (4h)

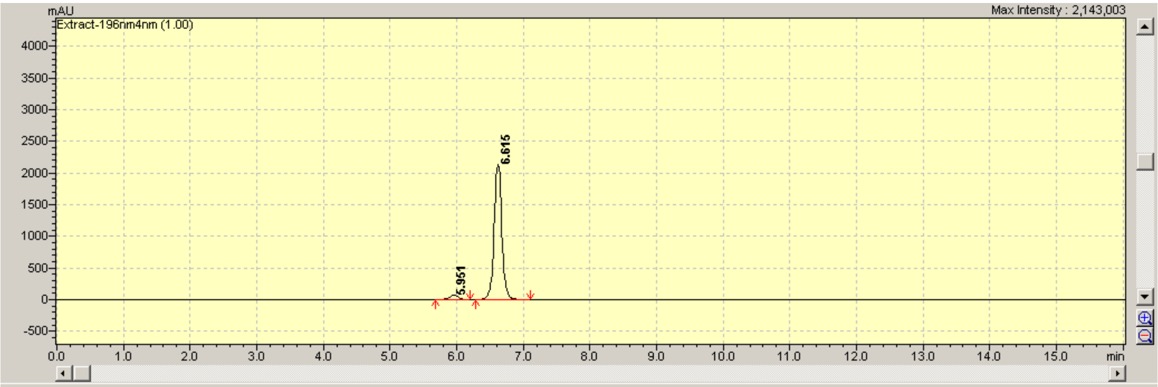

Peak Table

Peak#	ID#	Name	Ret. Time	Conc.	Area	Height	Similarity Index	Mark	Peak Start	Peak End	Area%
1			5.951	3.64584	649184	71998	0.000000		5.685	6.208	3.6458
2			6.615	96.35416	17156977	2136981	0.000000		6.283	7.104	96.3542

Extracted Chromatogram 196nm / Ch1 254nm

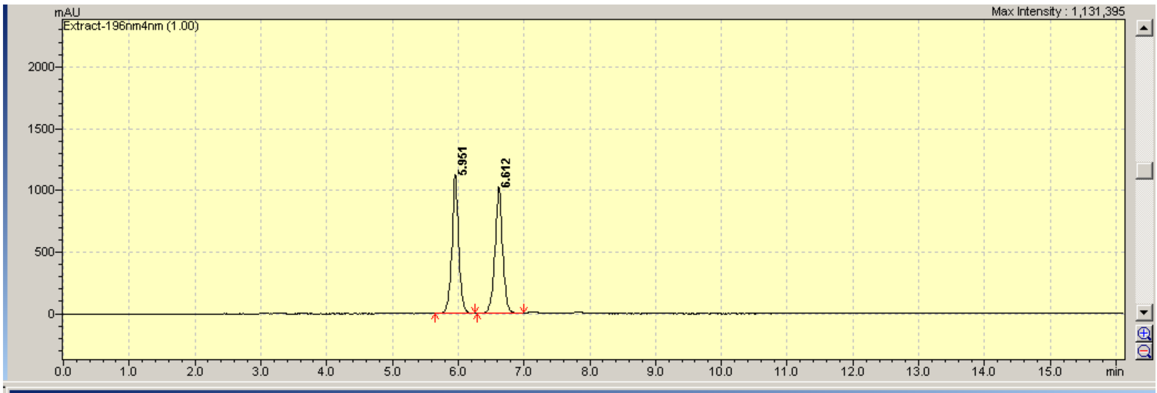

Peak Table

Peak#	ID#	Name	Ret. Time	Conc.	Area	Height	Similarity Index	Mark	Peak Start	Peak End	Area%
1			5.951	49.39948	8007205	1127821	0.000000		5.653	6.251	49.3995
2			6.612	50.60052	8201882	1020961	0.000000		6.293	6.997	50.6005

Extracted Chromatogram 196nm / Ch1 254nm

methyl (R)-2-((3-bromophenyl)(10-oxo-9,10-dihydroanthracen-9-yl)methyl)acrylate (4i)

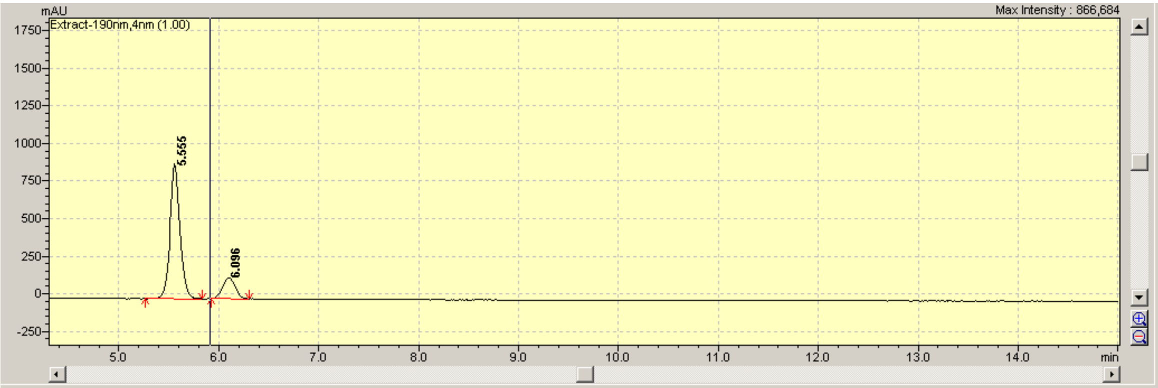

Peak Table

Peak#	ID#	Name	Ret. Time	Conc.	Area	Height	Similarity Index	Mark	Peak Start	Peak End	Area%
1			5.555	83.76693	6148370	898264	0.000000		5.259	5.835	83.7669
2			6.096	16.23307	1191483	140132	0.000000		5.920	6.304	16.2331

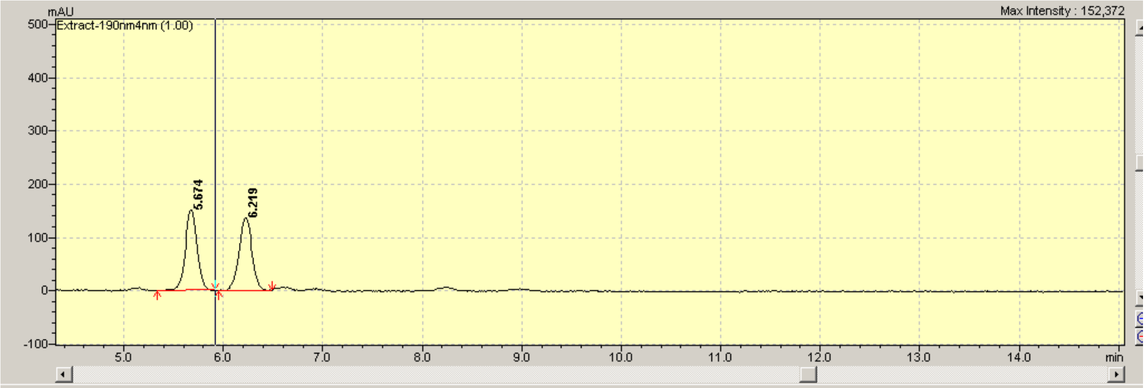

Peak Table

Peak#	ID#	Name	Ret. Time	Conc.	Area	Height	Similarity Index	Mark	Peak Start	Peak End	Area%
1			5.674	50.66090	1243980	151276	0.000000		5.333	5.920	50.6609
2			6.219	49.33910	1211524	136456	0.000000		5.952	6.485	49.3391

Extracted Chromatogram 190nm / Ch1 254nm

methyl (S)-2-((2-bromophenyl)(10-oxo-9,10-dihydroanthracen-9-yl)methyl)acrylate (4j)

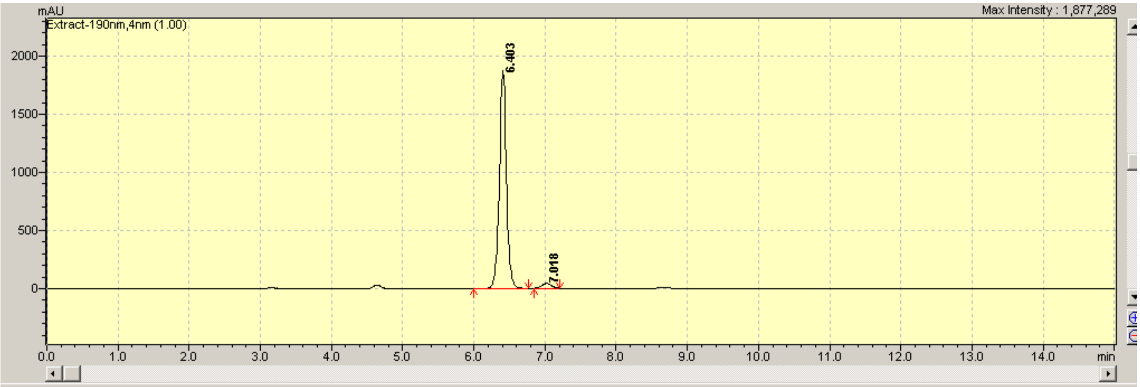

eak Table

Peak#	ID#	Name	Ret. Time	Conc.	Area	Height	Similarity Index	Mark	Peak Start	Peak End	Area%
1			6.403	97.05671	12980068	1875550	0.000000		6.005	6.773	97.0567
2			7.018	2.94329	393626	42347	0.000000		6.848	7.211	2.9433

Extracted Chromatogram 190nm | Ch1 254nm

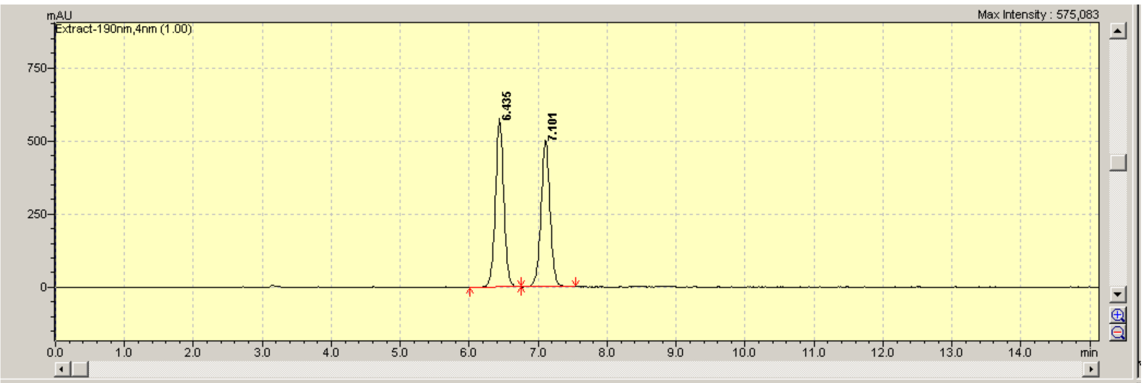

Peak Table

Peak#	ID#	Name	Ret. Time	Conc.	Area	Height	Similarity Index	Mark	Peak Start	Peak End	Area%
1			6.435	50.76713	4688139	573660	0.000000		6.016	6.752	50.7671
2			7.101	49.23287	4546456	501502	0.000000		6.752	7.541	49.2329

Extracted Chromatogram 190nm | Ch1 254nm

methyl (S)-2-((10-oxo-9,10-dihydroanthracen-9-yl)(thiophen-2-yl)methyl)acrylate (4k)

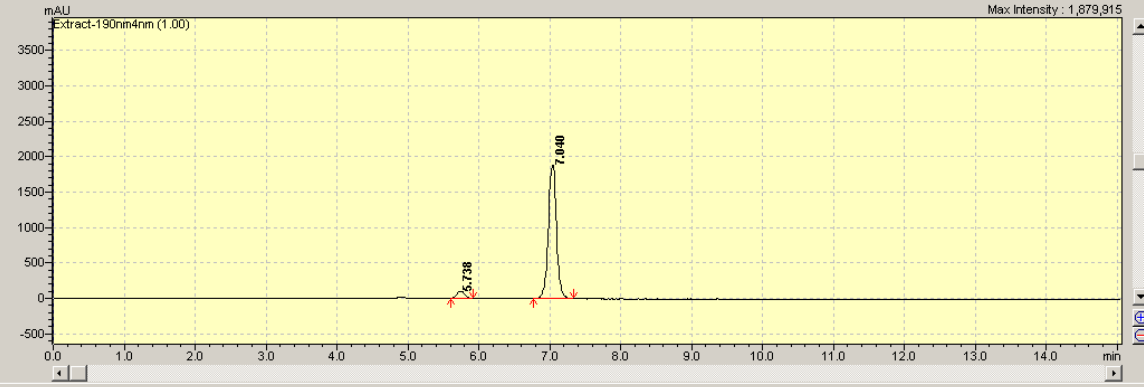

Peak Table

Peak#	ID#	Name	Ret. Time	Conc.	Area	Height	Similarity Index	Mark	Peak Start	Peak End	Area%
1			5.738	5.05356	788286	102499	0.000000		5.611	5.920	5.0536
2			7.040	94.94644	14810360	1882809	0.000000		6.763	7.339	94.9464

Extracted Chromatogram 190nm / Ch1 254nm

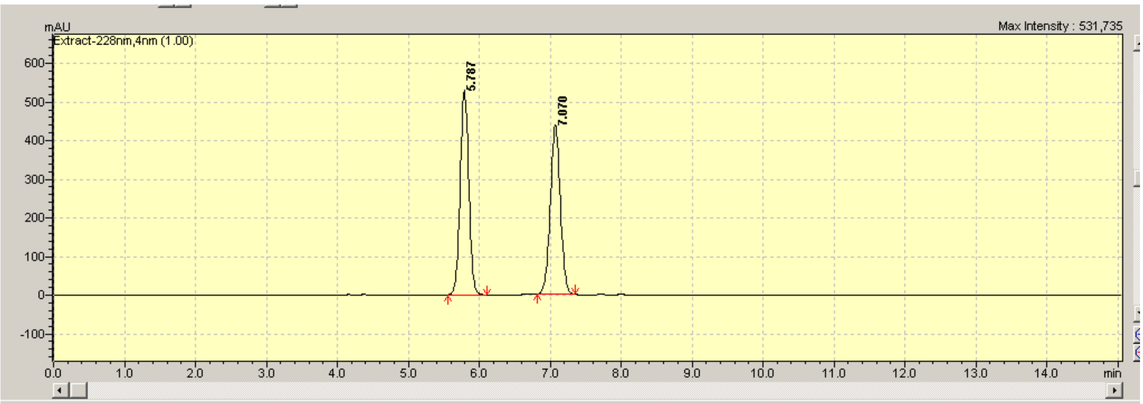

Peak Table

Peak#	ID#	Name	Ret. Time	Conc.	Area	Height	Similarity Index	Mark	Peak Start	Peak End	Area%
1			5.787	50.46503	4397367	531199	0.000000		5.557	6.112	50.4650
2			7.070	49.53497	4316324	437135	0.000000		6.827	7.349	49.5350

Extracted Chromatogram 228nm / Ch1 254nm

methyl (S)-2-methylene-3-(10-oxo-9,10-dihydroanthracen-9-yl)-5-phenylpentanoate (4l)

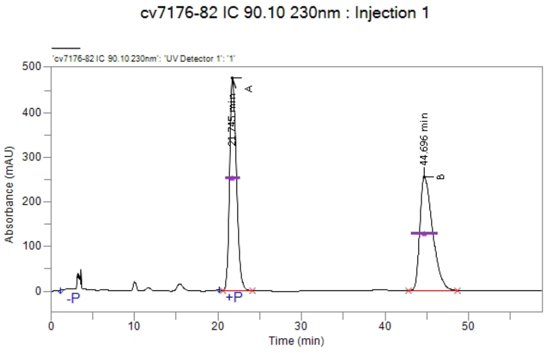

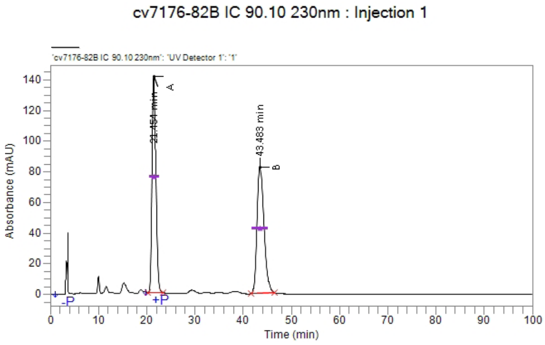

Time	Area	Area %
21.745	27,142,777.6	49.97
44.696	27,171,846.6	50.03
**Total**	54,314,624.3	100.00

Time	Area	Area %
21.454	7,770,143.8	48.24
43.483	8,336,226.7	51.76
**Total**	16,106,370.5	100.00

(R)-2-((10-oxo-9,10-dihydroanthracen-9-yl)(phenyl)methyl)acrylonitrile (4m)

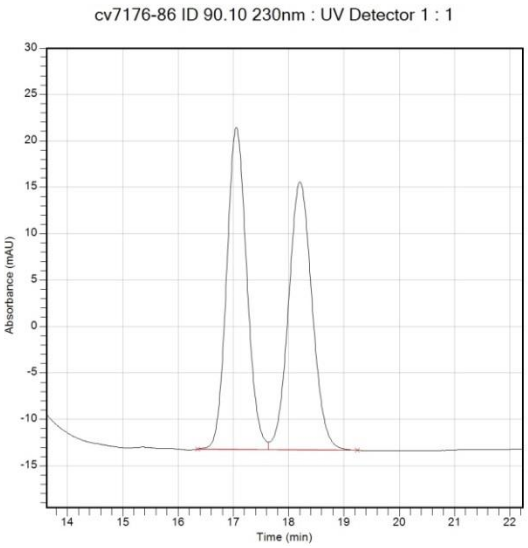

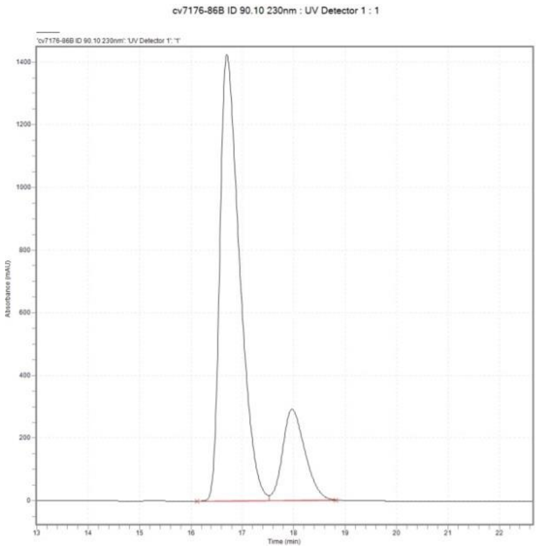

Time	Area	Area %
17.053	879,121.1	50.57
18.204	859,182.6	49.43
**Total**	1,738,303.8	100.00

Time	Area	Area %
16.699	38,039,234.4	81.24
17.967	8,784,525.4	18.76
**Total**	46,823,759.8	100.00

(R)-2-((10-oxo-9,10-dihydroanthracen-9-yl)(p-tolyl)methyl)acrylonitrile **(4n)**

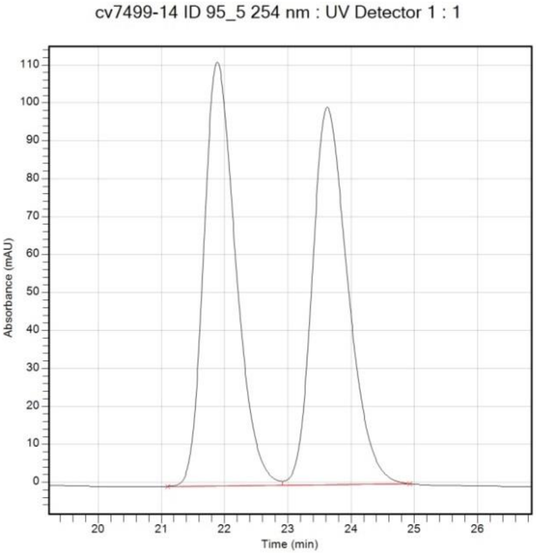

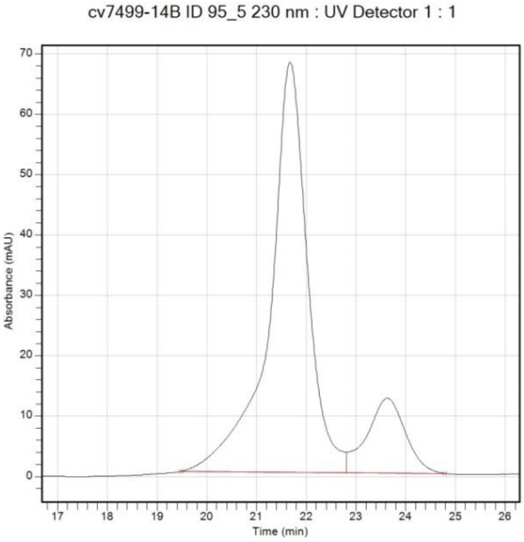

Time	Area	Area %
21.884	3,923,612.5	50.16
23.623	3,898,598.6	49.84
**Total**	7,822,211.1	100.00

Time	Area	Area %
21.673	3,585,171.7	84.38
23.630	663,853.2	15.62
**Total**	4,249,024.9	100.00

(R)-10-(2-methylene-3-oxo-1-phenylbutyl)anthracen-9(10H)-one (4o)

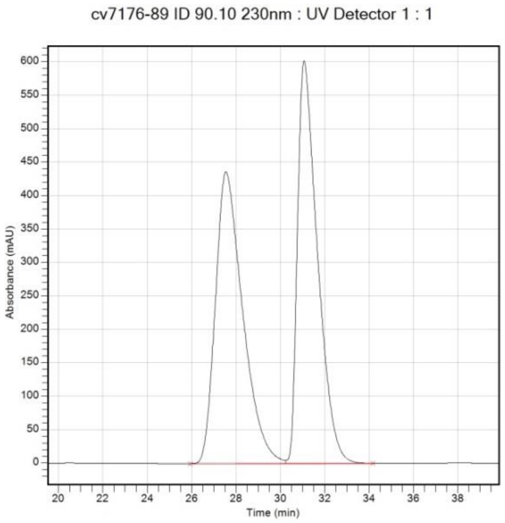

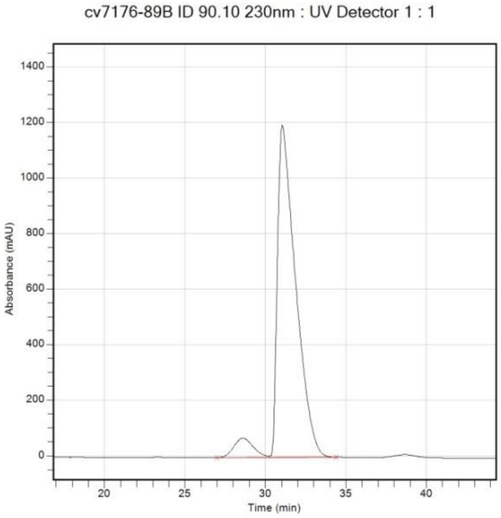

Time	Area	Area %
27.543	37,318,906.9	49.73
31.071	37,718,639.2	50.27
**Total**	75,037,546.1	100.00

Time	Area	Area %
28.600	5,922,313.2	5.96
31.046	93,528,206.1	94.04
**Total**	99,450,519.2	100.00

(R)-10-(2-methylene-3-oxo-1-(p-tolyl)butyl)anthracen-9(10H)-one (4p)

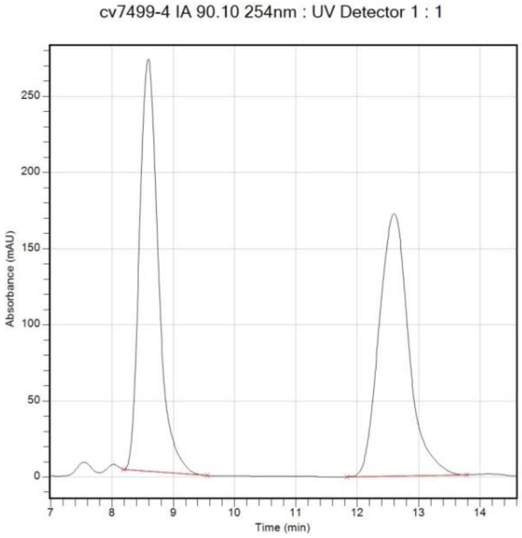

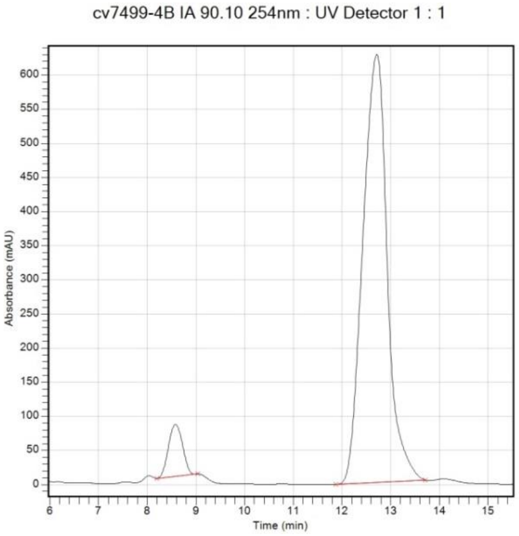

Time	Area	Area %
8.591	5,782,567.2	49.94
12.602	5,796,995.4	50.06
Total	11,579,562.5	100.00

Time	Area	Area %
8.579	1,536,193.8	6.58
12.714	21,814,617.6	93.42
Total	23,350,811.4	100.00

(R)-10-(2-methylene-1-(4-nitrophenyl)-3-oxobutyl)anthracen-9(10H)-one (4q)

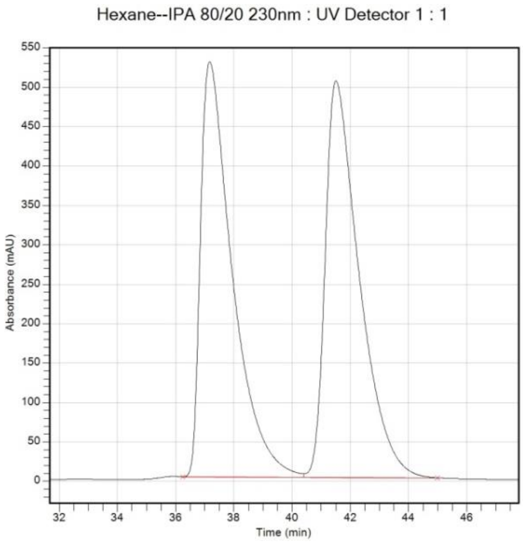

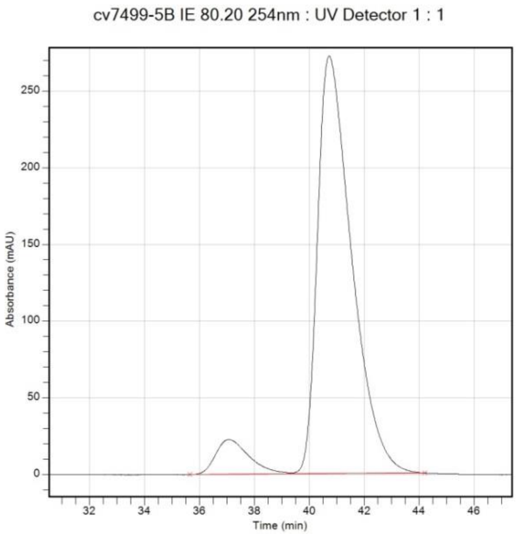

Time	Area	Area %
37.170	39,923,374.7	49.45
41.506	40,809,178.7	50.55
**Total**	80,732,553.4	100.00

Time	Area	Area %
37.071	1,934,368.4	7.58
40.725	23,571,697.8	92.42
**Total**	25,506,066.3	100.00

(R)-10-(1-(4-bromophenyl)-2-methylene-3-oxobutyl)anthracen-9(10H)-one (4r)

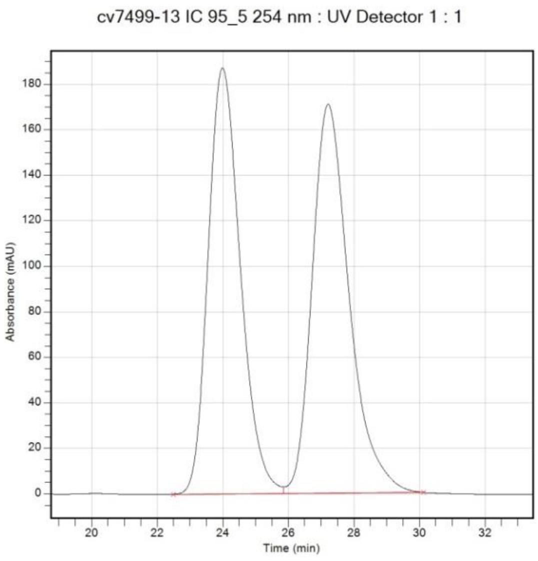

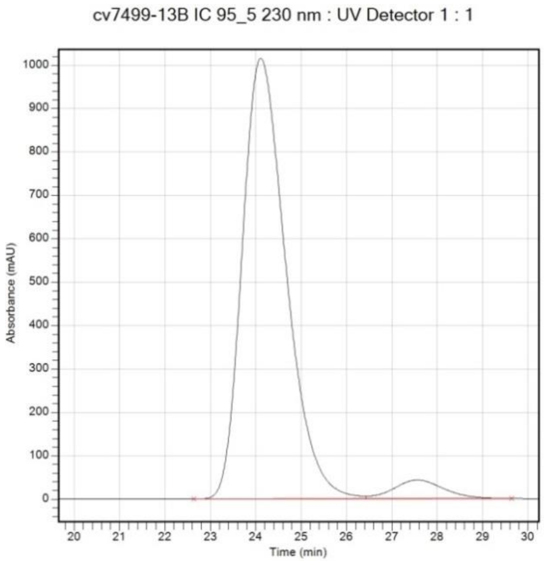

Time	Area	Area %
23.991	12,771,777.9	48.81
27.215	13,394,673.4	51.19
**Total**	26,166,451.4	100.00

Time	Area	Area %
24.110	67,785,904.0	95.94
27.558	2,868,594.8	4.06
**Total**	70,654,498.8	100.00

(R)-10-(1-(4-chlorophenyl)-2-methylene-3-oxobutyl)anthracen-9(10H)-one (4s)

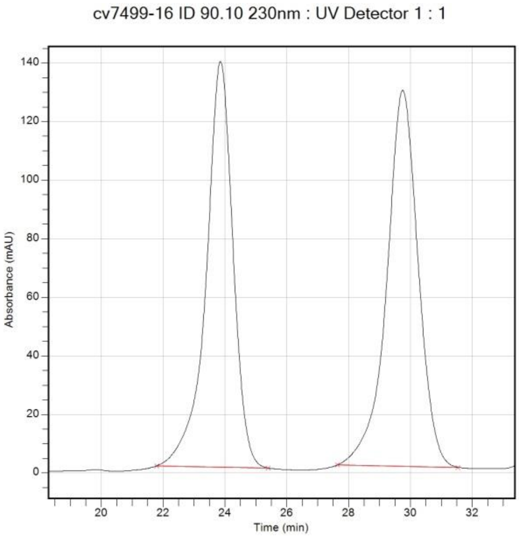

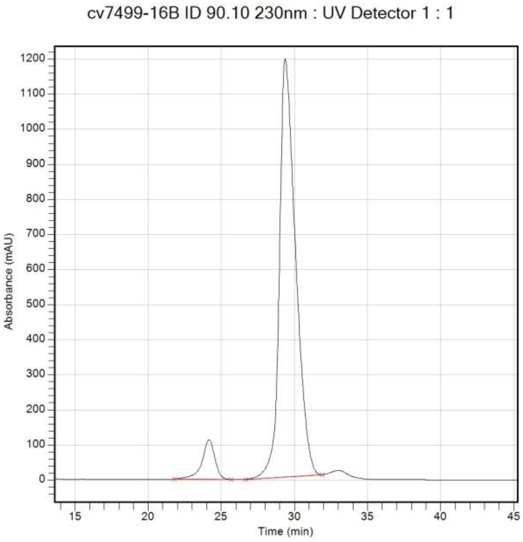

Time	Area	Area %
23.853	8,369,168.1	47.94
29.744	9,089,951.5	52.06
**Total**	17,459,119.6	100.00

Time	Area	Area %
24.159	4,487,619.5	4.90
29.365	87,134,970.0	95.10
**Total**	91,622,589.5	100.00

(R)-4-(2-methylene-3-oxo-1-(10-oxo-9,10-dihydroanthracen-9-yl)butyl)benzonitrile (4t)

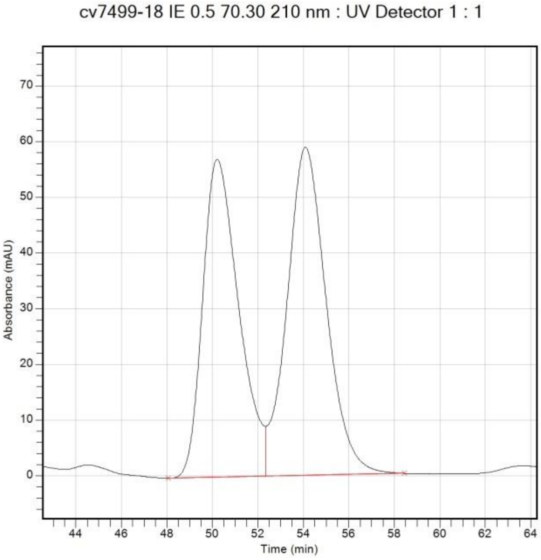

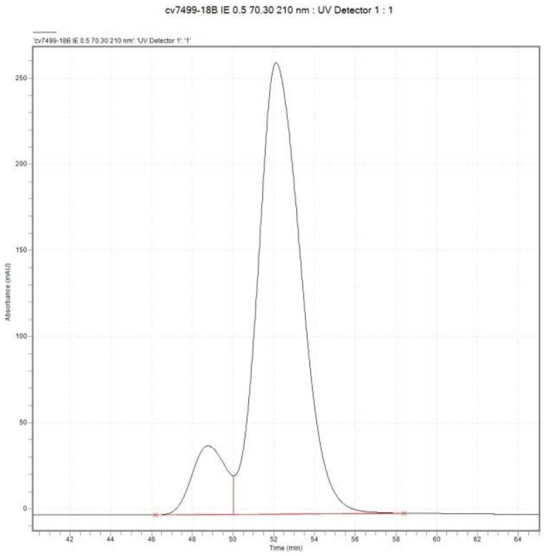

Time	Area	Area %
50.209	6,287,892.4	47.04
54.090	7,078,605.8	52.96
**Total**	13,366,498.2	100.00

Time	Area	Area %
48.775	4,597,341.1	10.75
52.112	38,151,943.6	89.25
**Total**	42,749,284.7	100.00

(R)-10-(2-methylene-3-oxo-1-phenylpentyl)anthracen-9(10H)-one (4u)

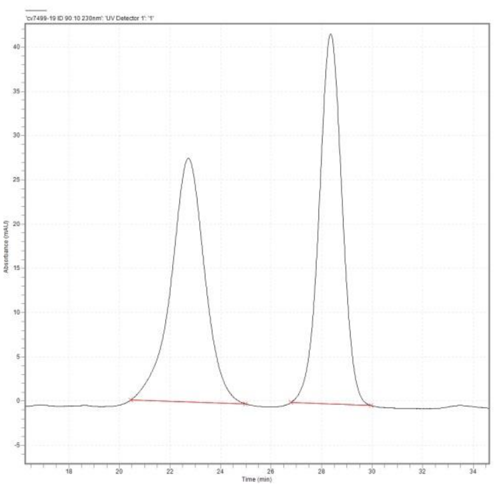

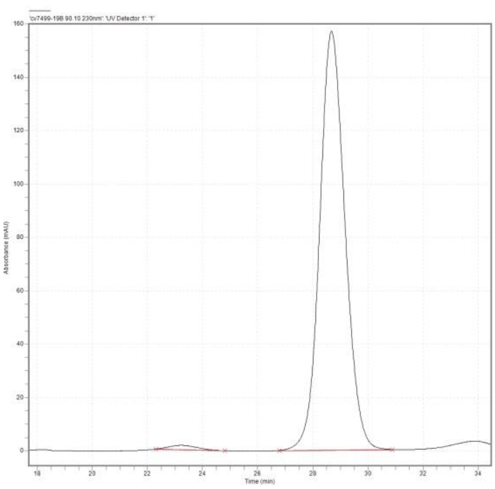

Time	Area	Area %
22.726	2,538,714.6	49.02
28.360	2,640,475.3	50.98
**Total**	5,179,189.9	100.00

Time	Area	Area %
23.243	119,310.6	1.18
28.679	9,994,167.7	98.82
**Total**	10,113,478.3	100.00

X-Ray data

methyl (R)-2-((4-fluorophenyl)(10-oxo-9,10-dihydroanthracen-9-yl)methyl)acrylate (4b)

Table 1 Crystal data and structure refinement for 2015vc001.

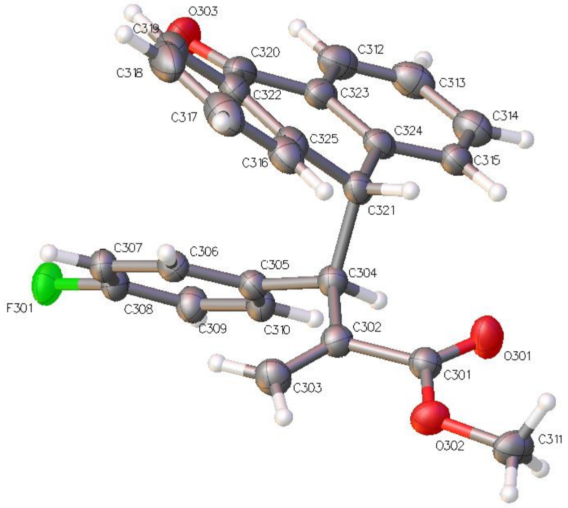

Identification code	2015vc001 (cv7499-67C)
Empirical formula	$C_{25}H_{19}FO_3$
Formula weight	386.40
Temperature/K	100(2)
Crystal system	orthorhombic
Space group	$P2_12_12_1$
a/Å	11.07781(8)
b/Å	12.29676(10)
c/Å	42.1557(3)
α/°	90
β/°	90
γ/°	90
Volume/Å^3	5742.50(8)
Z	12
ρ_{calc}g/cm^3	1.341
μ/mm^{-1}	0.767
F(000)	2424.0
Crystal size/mm^3	0.2 × 0.15 × 0.12
Radiation	CuKα (λ = 1.54184)
2Θ range for data collection/°	4.192 to 137.932
Index ranges	$-13 \le h \le 13$, $-14 \le k \le 14$, $-51 \le l \le 49$
Reflections collected	77409
Independent reflections	10586 [R_{int} = 0.0403, R_{sigma} = 0.0152]
Data/restraints/parameters	10586/0/788
Goodness-of-fit on F^2	1.076
Final R indexes [I>=2σ (I)]	R_1 = 0.0319, wR_2 = 0.0829
Final R indexes [all data]	R_1 = 0.0323, wR_2 = 0.0832
Largest diff. peak/hole / e Å^{-3}	0.20/-0.18
Flack parameter	0.00(3)

10-((1S,2S)-1-(4-bromophenyl)-2-methyl-3-oxobutyl)anthracen-9(10H)-one (5r)

Table 1 Crystal data and structure refinement for 2015VC003.

Identification code	2015VC003
Empirical formula	$C_{25}H_{21}BrO_2$
Formula weight	433.33
Temperature/K	100(2)
Crystal system	orthorhombic
Space group	$C222_1$
a/Å	14.1137(4)
b/Å	14.1141(5)
c/Å	39.2592(13)
α/°	90
β/°	90
γ/°	90
Volume/Å^3	7820.5(4)
Z	16
ρ_{calc}g/cm^3	1.472
μ/mm^{-1}	3.001
F(000)	3552.0
Crystal size/mm^3	0.156 × 0.115 × 0.1
Radiation	CuKα (λ = 1.54184)
2Θ range for data collection/°	4.502 to 137.968
Index ranges	$-17 \leq h \leq 16, -16 \leq k \leq 16, -47 \leq l \leq 47$
Reflections collected	57094
Independent reflections	7193 [R_{int} = 0.1076, R_{sigma} = 0.0386]
Data/restraints/parameters	7193/512/511
Goodness-of-fit on F^2	1.122
Final R indexes [I>=2σ (I)]	R_1 = 0.0663, wR_2 = 0.1794
Final R indexes [all data]	R_1 = 0.0702, wR_2 = 0.1868
Largest diff. peak/hole / e Å^{-3}	1.35/-2.32
Flack parameter	-0.036(10)

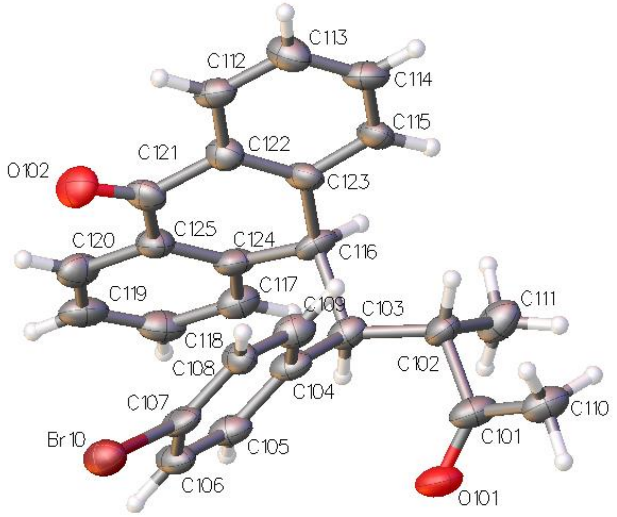

Supplement: Supplementary Information [file srep16886-s1.pdf]
